# Hyperadaptability Through Self-Organizing Behavioral Search

**DOI:** 10.3390/e28091013

**Published:** 2026-09-11

**Authors:** Alex Baranski, Jun Tani

**Affiliations:** Cognitive Neurorobotics Research Unit, Okinawa Institute of Science and Technology Graduate University, 1919-1 Tancha, Onna-son, Okinawa 904-0495, Japan; jun.tani@oist.jp

**Keywords:** behavioral search, behavioral adaptation, binary entropy, self-organization, complex adaptive systems, autonomous agents, problem solving, information theory

## Abstract

Artificially replicating the extraordinary adaptive potential of organisms remains difficult. Machine learning approaches based on big data pursue behavioral adaptation through generalization from training data, but often learn slowly and struggle with out-of-distribution situations. We propose that organisms may not adapt primarily through generalization alone but by rapidly eliminating infeasible solutions through online trial-and-error, effectively performing a search over the behavior space. If this search is complete, it is guaranteed to find an existing physically robust solution within a finite but unbounded time. For continuous behavioral domains that contain uncountably infinite behaviors, we introduce a mathematical framework for constructing a countably infinite dense subset of all behaviors using a mutable graph to segment behavior space, allowing any behavior to be progressively approximated arbitrarily well. Graph evolution is regulated by a heuristic feedback loop between outward growth and internal refinement; refinement is partially determined by a Bernoulli variance term related to binary entropy. Using this construction, we implement a proof-of-concept behavioral search algorithm and evaluate it on maze navigation and simple continuous control tasks. These preliminary results establish the practical feasibility of this approach in low-dimensional simulated environments while exposing unresolved limitations in terms of dimensional scaling and the incorporation of prior information.

## 1. Introduction

The world is always changing, and to survive, one must change with it. In particular, *adaptability* is the capacity to change *usefully* in response to new goals and circumstances. The advantage enjoyed by an organism with some degree of behavioral adaptability is clear: by being able to generate novel behaviors to achieve a goal, the organism’s ability to satisfy its wants and needs becomes partially invariant to the nature of those wants and needs, the environment it finds itself in, and even its own prior experience and insight. In a sense, the potential *degree* of behavioral novelty is a measure of the adaptability of an organism. To clarify the underlying mechanisms of behavioral adaptability, we propose to study its theoretical limit, which we call *hyperadaptability*: the guaranteed knowledge-invariant ability to generate a behavior to achieve any achievable goal, in any context, within finite time.

Of course, practical limitations on cognitive resources ensure that nothing is *truly* hyperadaptable; nevertheless, it provides a useful theoretical lens through which to view behavior and adaptation, in much the same way we say that physical computers are Turing-complete [1] even though Turing-completeness requires infinite memory, which no real computer has. In this paper, we study the problem of how ethogenesis must occur in order to achieve hyperadaptability, ultimately offering the following solution: the solution to every solvable problem exists in an *infinite space of all possible behaviors*. By searching this space *systematically*, but not necessarily *unintelligently*, an organism can *guarantee* that it finds *a* solution. However, this space, which we call the *real behaviors* in analogy to R, is *uncountably infinite* and *unbounded*, meaning that it *cannot be searched*. This apparent impasse is resolved by constructing *dense* and *countably infinite subsets* of the real behaviors; in this sense, one set is *dense* in another if every element of the latter is arbitrarily close to one element of the former. By virtue of their countability, any of these sets can be systematically *searched* by simply *enumerating* them, and since they are *dense* in the set of *all real behaviors*, an agent searching one of these sets will eventually get arbitrarily close to any desired “real” behavior. Because of this, in analogy to Q, we call them *rational*.

Enumerating a rational set of behaviors by trying each of them in turn until one solves an arbitrary problem is equivalent to *searching it for a solution*, which due to the countability of the set, is guaranteed to occur in finite time, thereby enabling *hyperadaptability*. As we will see, the enumeration suggested by the structure of the behavior space lends itself to a very natural interpretation in terms of cognitive heuristics for trial-and-error exploration. We formalize this *adaptation-as-search* perspective with a theory of *behavioral search* in which the *state* of a *cognitive graph* [2] serves as an *index structure* for a set of rational behaviors. We use this theory to design an algorithm that operates over the cognitive graph and then test it in simulated environments.

Search has long been an important problem-solving method in discrete and low-dimensional domains, but in many modern settings it has been displaced by methods based on local optimization of parameterized function approximators, especially deep neural networks. Fundamentally, the success of these systems is based on their ability to find patterns in their training data that generalize to new samples. However, successful generalization has proven to be enormously costly. In effect, the drive to generalize can be seen as an attempt to *avoid* adaptation, as successful generalization is effectively a form of “pre-adaptation” to novel problems. While effort to design learning algorithms that generalize better is surely worthwhile, our view is that generalization is, fundamentally, just epistemologically hard, and that perhaps at the current levels of effort directed towards generalization, the exchange rate between trying to generalize from training data and just performing structured online search at “test time”, i.e., adaptation, probably favors adaptation.

Our work overlaps in both concept and method with a variety of methods. Goal-conditioned reinforcement learning learns policies or value functions that can be directed towards different goals, with hindsight experience replay allowing failed attempts to be reused as successful experience for goals that were actually achieved [3,4]. Archive-based methods such as Go-Explore explicitly remember promising states and return to them before exploring further [5]. Intrinsically Motivated Goal Exploration Processes (IMGEPs) autonomously generate and order goals to acquire diverse skills [6], while novelty search and quality diversity methods maintain archives or populations of diverse solutions [7,8]. Sampling-based planners such as Rapidly-Exploring Random Trees (RRT) can incrementally explore a continuous space of configurations. These are all viable alternative approaches to exploration and behavioral adaptation. However, to the best of our knowledge, these methods do not altogether provide a means of constructing an enumerable family of continuous behaviors that (i) is dense in the full space of all behaviors, (ii) is driven by local rules to expand scope and increase precision via physical execution of behaviors, (iii) does not require an external reward or reset mechanism, (iv) can be dynamically re-ordered in an experience-dependent way without compromising the density requirement, and (v) is cognitively plausible. This is the specific gap that is addressed by the theoretical construction presented in this paper. The ARMS algorithm is a finite-memory proof-of-concept inspired by that construction, and the present experiments do not seek to establish ARMS as a generally superior alternative to these other methods.

### 1.1. Behavioral Search

Here, we adopt one principled formulation of behavioral search. Rather than treating the point of behavior as maximizing a reward function that implicitly defines a task, we view it as solving a *problem*, namely, being in one part of state space and wanting to be in another. State space is construed broadly, and a goal specifies the region that the agent wants to reach.

In our formulation, behavior has two aspects: an *aspirational* plan, and the actual trajectory generated by a low-level goal-directed controller attempting to execute that plan. Plans are sequences of waypoints or sub-goals, with a start-point and target-point forming a *1-plan*, while more complex plans are sequential compositions of 1-plans. A complex plan succeeds if all of its constituent 1-plans succeed, and otherwise fails. The agent searches the plan space by trying plans until one succeeds, without intrinsically requiring an externally supplied reset.

Enumerating a rational family of plans requires an *index structure* and a means of incrementing that index. Our choice of index structure is a *segraph*, of which the vertex fields segment the state space and the directed edges represent sets of possible 1-plans between fields. A path through the segraph represents a bundle of plans, from which the agent samples a particular sequence of waypoints to attempt.

Each edge records successful and failed traversals, allowing the agent to provisionally generalize from sampled 1-plans to other plans represented by the same edge. Failed regions of plan-space can later be probed at higher resolution by *refinement*, while *extension* adds vertices covering previously uncovered regions of state space. Under the ideal construction, balancing extension and refinement produces a countably infinite family of rational plans that is dense in the set of all plans. Exhaustively searching this family eventually approximates any required robust plan to sufficient precision.

The Adaptive Real-Time Metasearch over Segraphs (ARMS) algorithm is a finite-memory heuristic inspired by this construction. In particular, ARMS deletes under-used vertices once the graph reaches a fixed size (Appendix B.4). Because deletion is not containment-preserving, the assumptions underlying Proposition A1 and the corresponding completeness result do not apply to ARMS as implemented. ARMS should be understood as a proof-of-concept rather than as an implementation that inherits the true hyperadaptability guarantee.

### 1.2. What Is and Is Not Assumed

We have dropped several important assumptions that are common in DRL, but have been obliged to adopt some other assumptions which are *not* common in DRL and which DRL provides many suitable techniques for managing, which in the future could allow for a fruitful hybridization. The two major assumptions that we adopt are (i) that the state space of an agent is fully visible and (ii) that observations, actuators, and dynamics are all noiseless. In practice, these are severe constraints. While there are many powerful denoising methods [9,10,11], and Takens’ embedding theorem [12] tells us that given enough time-delayed *noiseless* observations of even a single scalar-measurement of a dynamical system, it is possible to reconstruct its state space up to diffeomorphism, solving these problems in combination for a forced system (that is, estimating latent state from noisy delayed observations of a non-autonomous dynamical system) is extremely difficult. Loosening these assumptions will be an important direction for future research.

In this work, we do *not* assume that there are any rich external rewards that can guide ethogenesis, nor that there will be externally provided resets of the environment. To us, these two assumptions are the least realistic in common DRL practice and represent the most serious block to the use of DRL “in the wild”. As a corollary, we are studying agents that exist, in a sense, only for a single problem, with no transfer of knowledge between problems, in order to model a lack of prior knowledge. Obviously, transfer of knowledge will *accelerate* problem-solving, but for theoretical reasons we are interested in investigating an information-impoverished scenario.

To be clear, though our algorithm does not *require* external resets to function, it is nevertheless compatible with their presence. Part of the empirical validation of our algorithm involves comparison against a standard reinforcement learning algorithm which requires environment resets during implementation to function properly. This comparison is conducted in a standard reinforcement learning environment which has resets. To allow a fair comparison and ensure that both algorithms experience identical conditions, our algorithm is also exposed to environmental resets; however, this reset is not “signaled” to the algorithm, and so appears from its “perspective” as an instantaneous and anomalous state change.

### 1.3. Contributions of This Paper

This paper details five primary contributions of our research to knowledge in the field of cybernetics and artificial intelligence:

1. We introduce a novel idealized limit for autonomous behavioral adaptability, which we call *hyperadaptability*. Hyperadaptability acts as the theoretical lens through which we view the challenge of designing a reliable and robust problem-solving agent.
2. We argue that hyperadaptability can and actually must be achieved by behavioral search, a complete search over the set of all possible plans for a behavior. While strictly impossible for the domain of continuous problems that we are focused on, we describe a subset of all plans, which we call the “rational plans”, that approximates the full set of all plans arbitrarily well; this subset *can* be searched to ensure hyperadaptability.
3. We develop a formalization of behavior that allows this subset to be constructed, for which we use a graph-based method inspired by the hippocampus. This formalization is designed to make it easy to add natural and intelligent biases that shape the search through the space of all plans without at the same time *compromising* the completeness of the search.
4. We design a simple finite-memory algorithm to execute a specific instantiation of this graph-based construction. This algorithm prioritizes plans that are empirically reliable, robust, and simple over plans that are empirically unreliable, fragile, and complex, retaining the latter to the extent permitted by practical memory constraints.
5. We test our algorithm in maze navigation and continuous control environments, and compare it against one task-relevant RL baseline in order to test the basic empirical feasibility of behavioral search.

The first three contributions constitute the primary theoretical emphasis of this paper. The ARMS algorithm and its limited empirical evaluation are secondary contributions intended to demonstrate that the theoretical framework can be operationalized.

### 1.4. Motivation

Controlling the order in which behaviors are tried is equivalent to choosing an enumeration of the set of behaviors. Rather than storing an infinitely long list, we want a structure that acts as an index into this set, which we call a *behavioral index structure* (BIX), together with a successor-like function that increments or modifies that index.

An effective BIX must have several properties. First, it must rapidly record which behaviors or sub-behaviors have succeeded or failed, allowing successful components to be reused and failed components to be temporarily avoided. Second, it must control the degree to which the outcome of one behavior is generalized to nearby behaviors. Because an agent without prior knowledge cannot know the appropriate degree of generalization, this generalization must be provisional; the agent should initially avoid sufficiently similar alternatives while retaining the ability to return and explore them at higher resolution if other possibilities are exhausted. Third, the BIX requires an explicit success criterion indicating when a behavior is “good enough” to solve the problem. Finally, it should exploit the fact that complex behaviors can be decomposed into sub-behaviors and recombined, meaning that experience with one component can constrain many complete behaviors at once.

A graph naturally provides these desired properties. Vertices can represent regions of state space, directed edges can represent goal-directed sub-behaviors between those regions, and paths can represent their composition into more complex behaviors. Edge fields determine the degree of provisional generalization, traversal counts record success and failure, and graph mutation changes both the scope and resolution of the indexed behaviors. These considerations motivate the segraph and its extension and refinement operations developed in the following sections.

### 1.5. Notational Conventions

Scalars are denoted by normal lower-case italic letters (*a*), vectors by bold lower-case italic letters (***a***), and matrices by bold upper-case letters (A). Ordered sequences (and conceptually adjacent objects) are denoted by lower-case Fraktur-font letters (a). Sets of such sequences are denoted by upper-case Fraktur-font letters (A). If a is a sequence or a is a vector, then a[j] and a[j] are the 0-based j^th^ index of a and a, respectively. For finite-length a, if j<0 then we let a[j]=a[|a|+j], so that a[−1] is the last item of a, a[−2] the second-to-last item of a, etc. When appropriate, we will sometimes extend this notation to subscripts, so we may write that a sequence $a=(a_{0},a_{1},a_{2},...,a_{-1})$, with $a_{-1}$ indicating the last element of the sequence. If we have a set of sets $A=\{A_{1},A_{2},...\}$, then we let 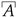 $={⋃}_{A^{\prime }\in A}A^{\prime }$. We use the same notation to refer to a union of elements (sets or otherwise) of a sequence.

## 2. Problems, Behaviors, and Solutions

Imagine that an agent A inhabits a Euclidean state space *S*, upon which it can act through an action space *A* via some deterministic dynamics. In this work, we do not consider the problem of *finding* nor *accessing knowledge about* such a state space, nor do we consider the problem of *noise*, instead granting the agent direct access to *S* and the immediate consequences of its actions on its state. A *problem* is when the agent *wants* to be at a specific state $s^{*}\in S$, but is currently in a different state $s\in S,s\neq s^{⋆}$. A *solution* is a *behavior* that takes the agent from s to $s^{⋆}$. In general, not all of state space may be physically accessible to our agent. We refer to the subset of *S* accessible from the agent’s starting state as $S_{0}\subseteq S$. While our agent may have a specific goal it wants to reach, a hyperadaptable agent should be able to eventually find the right behavior to reach *any* goal in $S_{0}$.

What exactly *is* a behavior? Ultimately, we will use the word to loosely refer to three different levels of abstraction:

1. A physically executed trajectory.
2. A plan that deterministically generates a physically executed trajectory.
3. A probability distribution over plans.

At first glance, the first sense would seem to be sufficient, but later we will want to speak of specific behaviors “failing” or “succeeding”, giving *behaviors* a teleological component that is lacking in a mere *physical trajectory*. Tautologically, a physical trajectory reaches the final point it reaches; it cannot “fail” or “succeed” to reach that point, as there is no intrinsic aspirational quality to such a trajectory: by definition, it reaches its final point. In order to grant behaviors a proper teleology, we restrict our consideration to behaviors that are generated by a stateful goal-directed low-level controller:

**Definition** **1** (Controller)**.** *A controller is a tuple $\left(K{,}^{+}x{,}^{-}x\right)$, where:*

- *K is a function $\left(a_{t},h_{t+1}\right)=K\left(s_{t},s^{⋆},o_{t},h_{t}\right)$ which maps a current state $s_{t}$, a target state $s^{⋆}$, any additional observations $o_{t}$, and a hidden state $h_{t}$ to an action $a_{t}$ and the controller’s next hidden state $h_{t+1}$.*
- *x+ is an indicator function that decides whether the controller has reached its target. If it has, then x+(ht)=1; otherwise, x+(ht)=0.*
- *x− is an indicator function that decides whether the controller should* give up* on trying to reach its target. If so, then x−(ht)=1; otherwise x−(ht)=0.*

*This definition can be suitably modified for continuous-time domains, which must be discretized anyways for computer implementation. For brevity, we generally refer to the whole controller $\left(K{,}^{+}x{,}^{-}x\right)$ simply as K, mentioning x+ and x− only when necessary.*

**Figure 1:**
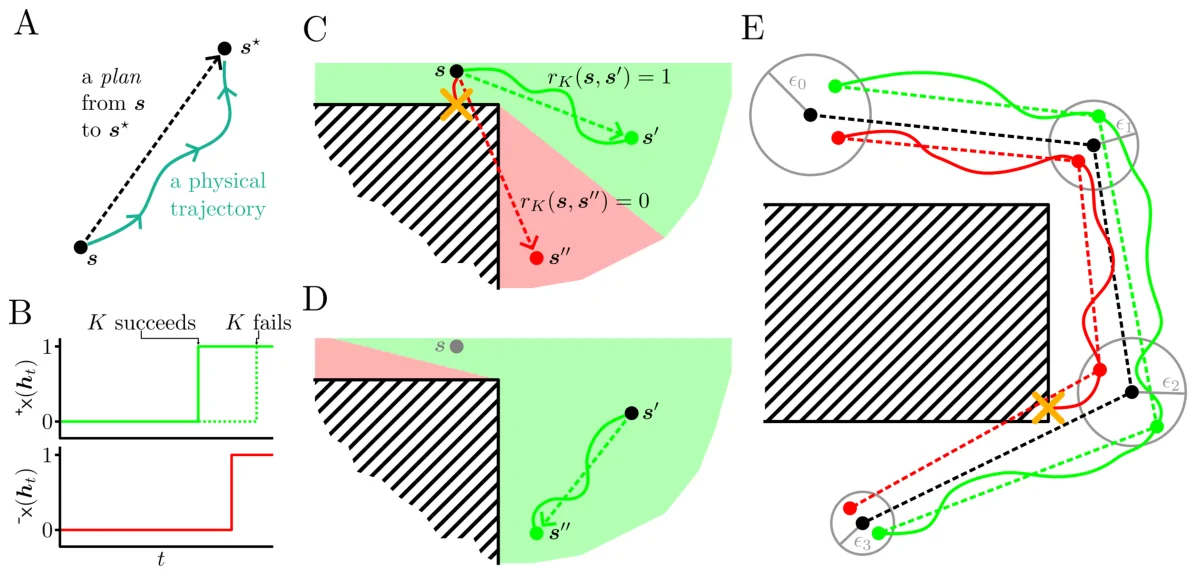
(**A**) A plan is a teleological object representing an intention to go from one state to another. Using a controller to follow a plan generates a physical trajectory. If the physical trajectory reaches the target, the plan *succeeds*; otherwise, it *fails*. (**B**) Termination is ultimately determined by the relative timing of the success and failure functions x+ and x−, so if x+ is 1 before x− is, the agent succeeds, otherwise the agent fails. (**C**) Some points, such as $s^{\prime }$, can be reached by the controller from s. Many points, such as $s^{\prime \prime }$, cannot. (**D**) Some points that cannot be reached in one step can be reached in multiple steps, i.e., if the agent first goes to $s^{\prime }$, then it can subsequently reach $s^{\prime \prime }$. (**E**) Complex plans (black dashed line) can be created by sequentially composing waypoints (black dots), generating more complex physical trajectories. For each plan, we can define a neighborhood of nearby plans, called its *ϵ-tube*. Alternative plans can be sampled from the ϵ-tube. Some may succeed (green behavior) and others may fail (red behavior); the more that succeed, the more robust the plan is.

The starting pair of target points $\left(s,s^{⋆}\right)$ corresponds to a very simple plan (Figure 1A). While both x+(ht)=0 and x−(ht)=0, the controller generates actions to reach the target, generating a physical trajectory or *execution*. As soon as x+(ht)=1 or x−(ht)=1, the execution is terminated and the plan either *succeeds* or *fails*, respectively (Figure 1B). x− is defined so that it will always eventually trigger, meaning that every execution ends at some finite time $T_{s,s^{⋆}}^{K}>0$, called the *termination time*. We denote the execution by K of the plan to reach $s^{⋆}$ from s as

(1) $$t_{K}^{\left(s,s^{⋆}\right)}:\left[0,T_{s,s^{⋆}}^{K}\right]\to S.$$

This is a “behavior” in the first sense. We define a *reachability function* $r_{K}\left(s,s^{⋆}\right)$ to indicate whether or not K reaches the goal $s^{⋆}$:

(2) rK(s,s⋆)=0,x−(hTs,s⋆K)=1,1,x−(hTs,s⋆K)=1.

See (Figure 1C) for an illustration. When $r_{K}\left(s,s^{⋆}\right)=0$, this implies that the controller K failed to go from s to $s^{⋆}$, while $r_{K}\left(s,s^{⋆}\right)=1$ implies it succeeded. Our method is comparable to (and formally, a specific manifestation of) the *options framework* [13].

We define ${\mathrm{con}}_{K}\left(s\right)=\left\{s^{⋆}\in S:r_{K}\left(s,s^{⋆}\right)=1\right\}$ as the set of points that K can reach from s, and define ${\mathrm{pre}}_{K}\left(s^{⋆}\right)=\left\{s\in S:r_{K}\left(s,s^{⋆}\right)=1\right\}$ as the set of points from which $s^{⋆}$ can be reached by K. For any sufficiently complex environment, the controller will not be able to reach most targets from most starting points, that is, $\exists s\in S_{0},S_{0}⊄{\mathrm{con}}_{K}\left(s\right)$ (Figure 1C). However, it may be the case that after reaching a point $s^{\prime }$ in ${\mathrm{con}}_{K}\left(s\right)$, *new* points then become reachable (Figure 1D); that is, it is possible that $\exists s^{\prime }\in {\mathrm{con}}_{K}\left(s\right)$ such that ${\mathrm{con}}_{K}\left(s^{\prime }\right)-{\mathrm{con}}_{K}\left(s\right)\neq ⌀$. If this is the case, then a point $s^{\prime }\in {\mathrm{con}}_{K}\left(s\right)\cap {\mathrm{pre}}_{K}\left(s^{\prime \prime }\right)$ can act as a *waypoint* between s and $s^{\prime \prime }$. We call a sequence of waypoints $s=(s,s_{1},s_{2},\ldots s_{n-1},s^{⋆})$ a *plan* from s to $s^{⋆}$ (Figure 1E). A plan is a behavior in the second sense.

We denote the composite execution generated by K following the plan s as $t_{K}^{s}$, which, like a “single-waypoint” execution, always terminates at some finite time $T_{s}^{K}$. We say that the plan succeeds if every sub-plan succeeds; however, if any of the sub-plans fails (a waypoint is not reached), then the entire plan fails and the execution terminates at a non-target point. We denote success in reaching the final target as $r_{K}\left(s\right)=1$ and failure as $r_{K}\left(s\right)=0$. We say that a plan s is *robust* if every “nearby” plan s succeeds. To formalize this, we first consider the set of length *n* plans with waypoints in *S*:

(3) $$S_{S}^{n}=\{{\left(s_{i}\right)}_{i=0}^{n}:s_{i}\in S\}.$$

We can imagine ascribing a “tolerance” or “nearness-threshold” $\epsilon_{i}$ to each waypoint $s_{i}$ in a plan s, defining the ϵ-neighborhood around $s_{i}$ as

(4) $$B_{\epsilon }\left(s\right)=\{s^{\prime }\in S:∥s-s^{\prime }∥\leq \epsilon \},$$

which is a hyperball with radius ϵ>0 centered on s. We can now introduce the *ϵ-tube*:

**Definition** **2** (ϵ-tube)**.** *Let $s={\left(s_{i}\right)}_{i=0}^{n}$ be a plan and $\epsilon ={\left(\epsilon_{i}\right)}_{i=0}^{n}$ a corresponding sequence of non-negative numbers. Then,*

$$N_{\epsilon }\left(s\right)=\left\{{\left(s_{i}^{\prime }\right)}_{i=0}^{n}\in S_{S}^{n}:\forall i\in \left[0.\;.n\right],s_{i}^{\prime }\in B_{\epsilon_{i}}\left(s_{i}\right)\right\}$$

*is the *ϵ-*tube* *around s, a bundle of “nearby” or “similar” plans (see Figure 1E for an example).* ϵ*-tubes will play a primarily epistemic role for our agent, allowing us to organize our agent’s assumptions about the navigability of the state space *S*. For now, we will use them to specify what behaviors are considered robust. Let*

(5) Nϵ+(s)={s′∈Nϵ(s):rK(s′)=1}

*be the set of all plans in the* ϵ*-tube of* s *which *succeed*. We extend the function* $r_{K}\left(s\right)$ *(the “reachability” of the plan) to its* ϵ*-tube. We refer to the *ϵ-reliability* of* s *as*

(6) rKϵ(s)=μ(Nϵ+(s))μ(Nϵ(s)),

*which is the fraction of successful plans in the* ϵ*-tube of* s*, with* μ(A) *being the measure of set *A*. It is helpful to consider different possible choices of* ϵ*. To this end, we construct* $E^{n}=\left\{{\left(\epsilon_{i}\right)}_{i=0}^{n}:\epsilon_{i}>0\right\}$ *as the set of possible* ϵ*-sequences of length *n*. This construction allows us to formally delineate between plans that are *robust* and those that are *not*:*

**Definition** **3** (Plan Robustness)**.** $$R_{K}\left(s\right)=\left\{\begin{array}{cc} 1, & \exists \epsilon \in E^{\ell (s)}\;s.t.\;r_{K}^{\epsilon }\left(s\right)=1,\\ 0, & ∄\epsilon \in E^{\ell (s)}\;s.t.\;r_{K}^{\epsilon }\left(s\right)=1. \end{array}\right.$$

*A plan s is * robust* if $R_{K}\left(s\right)=1$, otherwise it is *not.

Now, we let SSn+ be the set of all robust plans of length *n* and SS*+ the set of all robust plans of any finite length. Formally:

(7) SSn+={s∈SSn:RK(s)=1},

(8) SS*+=⋃n=1∞SS*+.

Then, slightly overloading the notation, we say that ${\mathrm{con}}_{K}^{*}\left(s\right)\subset S$ is the set of states that can be reached by K via some plan in SS*+. If $s_{0}$ is the initial state of the agent A, then $S_{K}={\mathrm{con}}_{K}^{*}\left(s_{0}\right)$ is the subset of *S* that A can reach. For the rest of this work, we assume that there are neither “Garden of Eden” nor “black hole” states (respectively, states or regions which, once left, can never be returned to and states or regions which, once entered, can never be left.) This means that $\forall s^{\prime }\in {\mathrm{con}}_{K}^{*}\left(s\right),{\mathrm{con}}_{K}^{*}\left(s^{\prime }\right)={\mathrm{con}}_{K}^{*}\left(s\right)$. Any state in $S_{K}$ is a potential problem that is physically solvable by our agent. An agent that is *hyperadaptable* can eventually solve any problem (reach any state) in $S_{K}$, meaning that it must be able to eventually find *a plan that reaches the goal*.

SSK*+⊂SSK* is the set of all possible robust plans with waypoints in $S_{K}$, with SSK*+(s,s′) the restriction of this set to only plans which start at s and end at $s^{\prime }$. Formally,

(9) SS*+(s,s′)={s∈SSK*+:s[0]=s,s[−1]=s′}.

**Figure 2:**
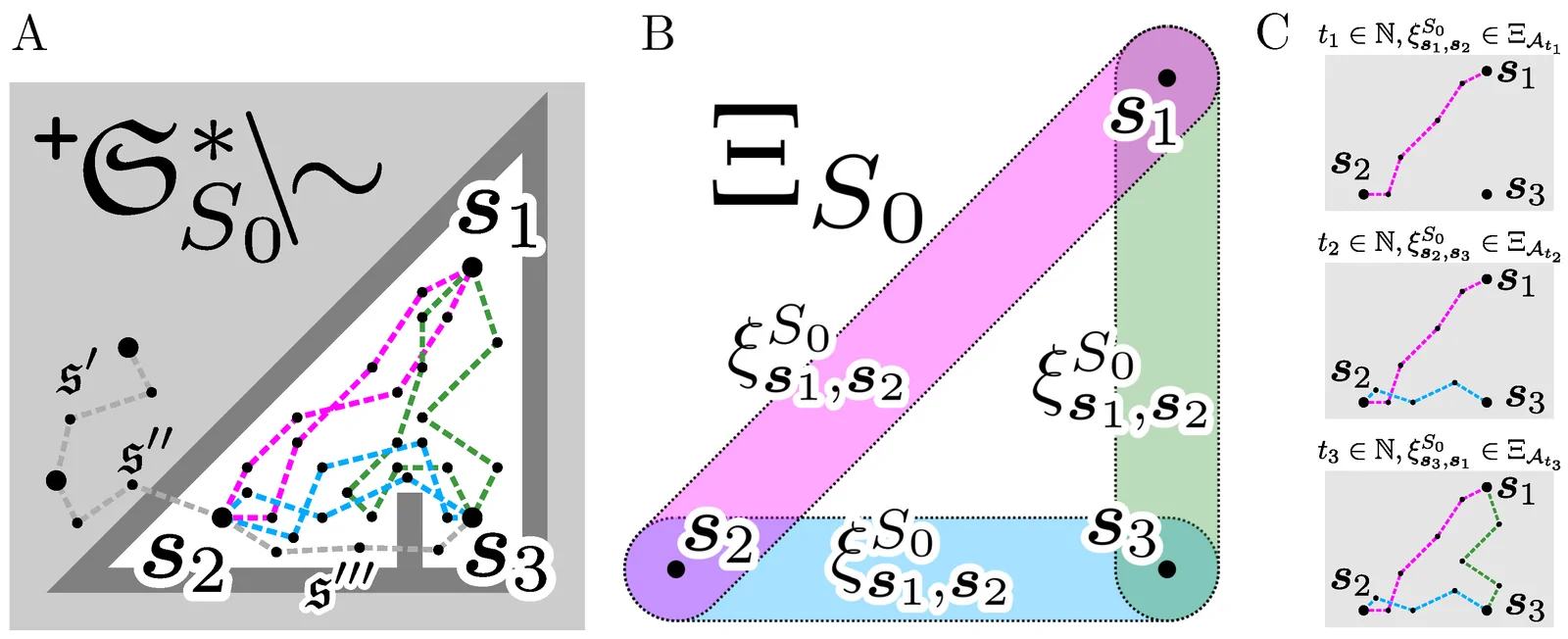
The set of all behaviors (plans) through state space is uncountably infinite, but there is an internal structure to this set which can be taken advantage of. (**A**) The set of interest for our agent is the set of all robust plans of any length with waypoints inside the reachable region of state space, SSK*+⊂SSK*⊂SS*, which excludes plans containing waypoints outside of $S_{K}$ (plans $s^{\prime }$ and $s^{\prime \prime }$) and any plan that fails or otherwise is not robust ($s^{\prime \prime }$ and $s^{\prime \prime \prime }$). All plans linking the same two points form an equivalence class (plans in the same equivalence class have the same color). The equivalence relation partitions the set of robust plans through $S_{K}$, SSK*+∖∼. (**B**) This partition is equivalent to the set of solvable problems $\Xi_{S_{K}}$ in the reachable state space; each achievement class ξ or set of equivalent behaviors corresponds to a solvable problem. (**C**) An agent is hyperadaptable if, for every solvable problem in $\Xi_{S_{K}}$, the agent finds a solution to that problem within finite time.

By definition, every plan in SSK*+(s,s′) is a viable way to go from s to $s^{\prime }$, so from the perspective of solving a problem (going from one point to another), every plan in SSK*+(s,s′) is equivalent (Figure 2A). We say that s∼s′⇔s,s′∈SSK*+(s,s′), and refer to the equivalence class SSK*+(s,s′) as an *achievement class*, denoted $\xi_{s,s^{\prime }}^{S_{K}}$. This construction is valid in this context because we are *specifically* not focusing on the *utility* of behaviors, such as time or energy spent or other factors, but *only* whether the behavior actually solves the problem. The set of all achievement classes partitions SSK*+, yielding a quotient set (Figure 2B):

(10) ΞSK=SSK*+/∼={ξs,s′SK:s,s′∈SK}

which effectively represents the existence of solutions to problems inside of $S_{K}$, called the *capability set* of $S_{K}$. If A is an agent and SA*+ is the set of behaviors that can be generated by A, then ΞSKA=SA*+/∼ represents the *capability* of A. Note that we have not yet described *how* A generates behaviors.

### 2.1. The Behavioral Search Problem

Let an agent A occupy a state $s_{0}\in S=R^{d}$ and possess a goal-directed controller K equipped with appropriate criteria for goal achievement success and failure. Given a target $s^{⋆}\in S_{K}$, the definition $S_{K}={\mathrm{con}}_{K}^{*}\left(s_{0}\right)$ implies that at least one robust plan from $s_{0}$ to $s^{⋆}$ exists. The behavioral search problem is to discover and physically execute, using K, a finite sequence of finite plans $s^{\left(1\right)},s^{\left(2\right)},\ldots ,s^{\left(\Omega \right)}$ for some unknown $\Omega \in N_{1}$, where every attempt terminates in finite time, each next plan begins from the state at which the preceding plan terminated, and the final plan $s^{\left(\Omega \right)}$ successfully terminates at $s^{⋆}$. No reward function, transition model, or external reset operation is assumed. Ideally, for any pair $\left(s_{0},s^{⋆}\right)\in {\left(S_{K}\right)}^{2}$, the method should construct such a sequence and solve the problem. ARMS is a finite-memory heuristic inspired by this ideal.

### 2.2. Notation for Plans

To simplify further exposition, it will help to introduce some notation and terminology related to plans.
We have already used s[i] to indicate the *i*^th^ element of s, where s[0] is the starting point of a plan, s[1] is the first waypoint in the sequence, and s[−1] is the final waypoint or “goal” of the plan.
If we have a plan $s={\left(s_{i}\right)}_{i=0}^{n}$, then we will say that ℓ(s)=n, so that the “goal” of a plan is s[ℓ(s)]. Notice that |s|=ℓ(s)+1, because |s| is the number of *states* in s, whereas ℓ(s) corresponds to the number of state-to-state “transitions” represented by s. These correspond to two different notions of the “length” of a plan, which this notation disambiguates.
If we have a plan s, and s is one of the states in the sequence, we will say that s is in s, written “s∈s”. This notation is slightly abusive, as it implicitly casts s as a sort of set, which it is not.
If s∈s, and s[i]=s, we will say that ${\mathrm{idx}}_{s}\left(s\right)=i$ to indicate the index of s inside the plan s.
If one behavior $s^{\prime }$ is contained by another plan s, we will say that $s^{\prime }$ is a “sub-behavior” of s, written $s^{\prime }⋐s$, and that s “contains” $s^{\prime }$, written $s⋑s^{\prime }$. Formally:
  (11) $$s^{\prime }⋐s\Leftrightarrow \exists k\in N\;\mathrm{such}\;\mathrm{that}\;\forall i\in \left[0.\;.\ell \left(s^{\prime }\right)\right],s^{\prime }\left[i\right]=s\left[i+k\right]\Leftrightarrow s⋑s^{\prime }$$
If two behaviors $s^{\prime }$ and $s^{\prime \prime }$ are sub-behaviors of s, and $s^{\prime \prime }$ happens “before” $s^{\prime }$ inside of s, we say that $s^{\prime \prime }$ is a pre-condition of $s^{\prime }$ within s, which we write as $s^{\prime \prime }\underset{s}{↣}s^{\prime }$. Formally:
  (12) $$s^{\prime \prime }\underset{s}{↣}s^{\prime }\Leftrightarrow \left(s^{\prime }⋐s\right)\wedge \left(s^{\prime \prime }⋐s\right)\wedge \left(\forall s^{\prime \prime }\in s^{\prime \prime },\;\forall s^{\prime }\in s^{\prime },\;{\mathrm{idx}}_{s}\left(s^{\prime \prime }\right)\leq {\mathrm{idx}}_{s}\left(s^{\prime }\right)\right).$$

## 3. Hyperadaptability and Behavioral Search

Before, we provided an informal definition of a hyperadaptable agent as one that can always eventually solve any solvable problem. We can now provide a formal definition of *hyperadaptability*.

**Definition** **4** (Hyperadaptability)**.** *Let $A_{t}$ be an agent at time t∈N.*

$$A\;\mathrm{is}\;\mathrm{hyperadaptable}\Leftrightarrow \forall \xi_{s,s^{\prime }}^{S_{K}}\in \Xi_{S_{K}},\exists t\in N\;s.t.\;\xi_{s,s^{\prime }}^{S_{K}}\in \Xi_{S_{K}}^{A_{t}}$$

*In other words, an agent is hyperadaptable if and only if, for any achievement class, there is some finite time at which that class is part of the agent’s capability (Figure 2C).*

You may notice that this definition makes no mention of any priors (crystal intelligence) or inference ability (fluid intelligence), as indeed hyperadaptability is independent of these notions. Fluid intelligence might allow a hyperadaptable agent to make more complex inferences about which behaviors could work, and prior knowledge might be able to narrow down the set of possible behaviors from the start, but hyperadaptability cannot depend on either of these concepts. In fact, because of this, an agent can only be hyperadaptable if it can perform an *exhaustive search* of $S_{S_{K}}^{*}$, as otherwise it would be possible for it to miss a necessary behavior. We immediately face a stark problem: $S_{S_{K}}^{*}$ is uncountably infinite (a property it inherits from *S*), meaning that it cannot be enumerated and that it is mathematically *impossible* to exhaustively search.

The apparent impasse is reduced by three observations. First, we can construct a countably infinite set of behaviors that is dense in $S_{S_{K}}^{*}$ and *can* be exhaustively searched. Second, the agent only needs to find one representative of each achievement class, rather than every plan solving the same problem. Third, plans share sub-plans, allowing the outcome of one attempted transition to constrain many other plans containing the same transition.

**Figure 3:**
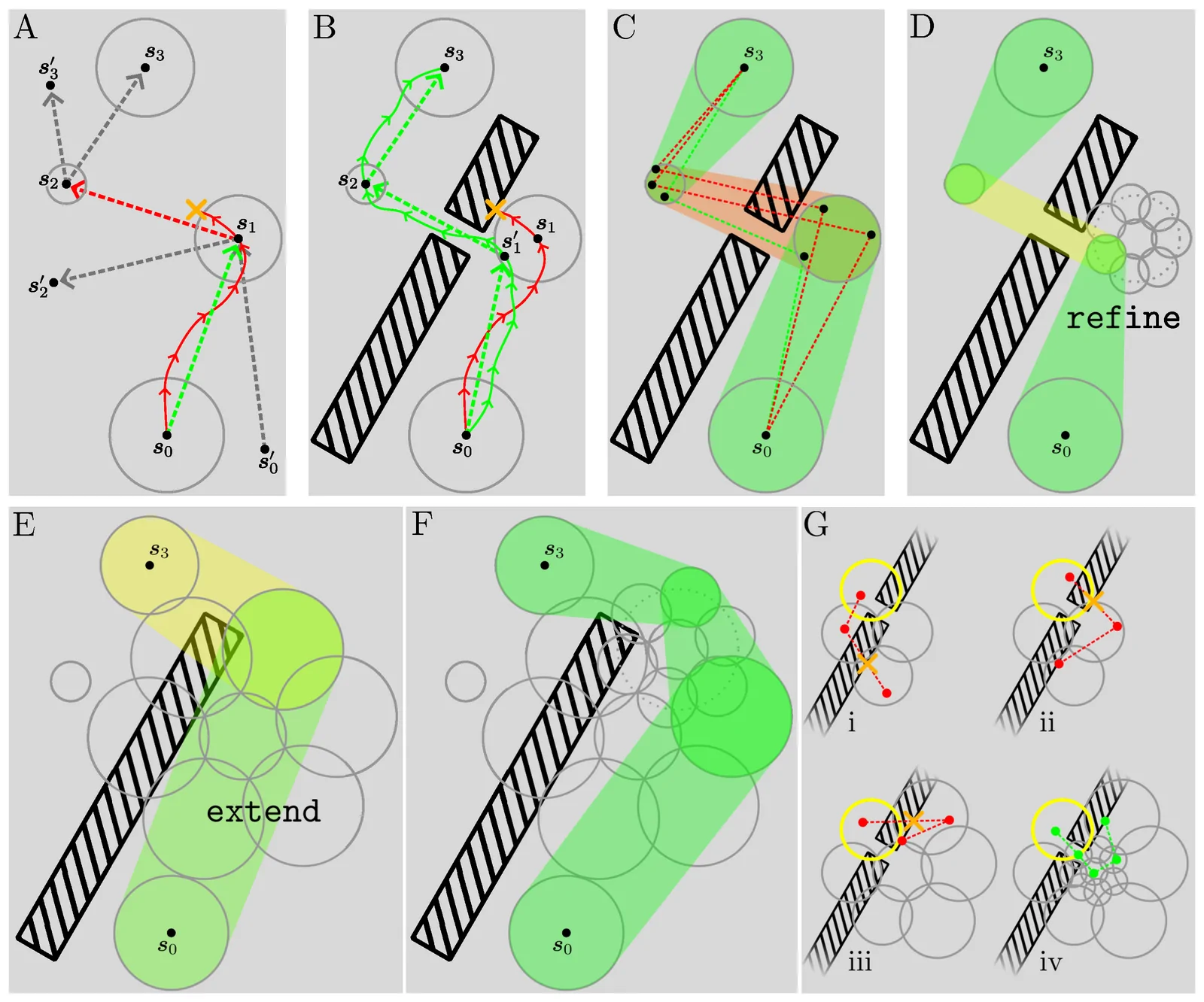
If a plan fails, where it fails and succeeds contains rich information about other plans. (**A**) If the plan $s=(s_{0},s_{1},s_{2},s_{3})$ fails between $s_{1}$ and $s_{2}$, then we know that any other plan that contains $\left(s_{1},s_{2}\right)$, such as $\left(s_{0}^{\prime },s_{1},s_{2},s_{3}^{\prime }\right)$, will *also* fail at $\left(s_{1},s_{2}\right)$. Conversely, any plan containing $\left(s_{0},s_{1}\right)$ will at least not fail at $\left(s_{0},s_{1}\right)$, such as $\left(s_{0},s_{1},s_{2}^{\prime }\right)$. (**B**) Just because $\left(s_{1},s_{2}\right)$ failed does not mean that other plans inside its ϵ-tube will also fail: it could be that $\left(s_{1},s_{2}\right)$ was a “near miss”. (**C**) If there is a solution in s’s ϵ-tube $⟦b_{s}⟧$, then randomly sampling plans can eventually find a solution. (**D**) A better way is to replace the single ϵ-tube with several higher-resolution ϵ-tubes by refining one of the waypoint’s hyperballs. (**E**) However, if a valid solution is not inside the original ϵ-tube, then the agent can add qualitatively new waypoints through extension. (**F**) Extension followed by refinement can create arbitrarily complex plans. (**G**) Ideally, the agent should attempt simpler and coarser plans before more complex and fine-grained plans (i → ii → iii → iv).

Suppose that $s=(s_{0},s_{1},s_{2},s_{3})$, with sub-plans $s_{1}=\left(s_{0},s_{1}\right)$, $s_{2}=\left(s_{1},s_{2}\right)$, and $s_{3}=\left(s_{2},s_{3}\right)$, and that $s_{1}$ succeeds while $s_{2}$ fails (Figure 3A). Under the deterministic conditions assumed here, every plan containing $s_{2}$ will fail at $s_{2}$ or before it, while a plan containing $s_{1}$ will at least not fail at $s_{1}$. The agent can deprioritize all plans containing the failed sub-plan. This does not justify permanently excluding nearby plans inside an ϵ-tube, since a slightly different transition may succeed (Figure 3B,C). Generalization from failure must remain provisional, with a mechanism for returning to the same region of the plan space at higher resolution.

To make these neighborhoods explicit, we replace waypoints with *wayregions*. Each waypoint $s_{i}$ is associated with a hyperball $b_{i}=B_{\epsilon_{i}}\left(s_{i}\right)$ and a plan $s=(s_{0},\ldots ,s_{-1})$ is associated with the sequence $b_{s}=\left(b_{0},\ldots ,b_{-1}\right)$. We write $s_{b}$ for the sequence of hyperball centers and $\epsilon_{b}$ for their radii. The corresponding bundle of plans is

(13) $$⟦b_{s}⟧=\underset{b_{i}\in b_{s}}{✕}b_{i}=\left\{{\left(s_{i}^{\prime }\right)}_{i=0}^{\ell (s)}:s_{i}^{\prime }\in b_{i}\right\}=N_{\epsilon_{b_{s}}}\left(s_{b_{s}}\right).$$

If this bundle contains a robust plan, random sampling could eventually find it, but a systematic search requires a means of increasing the resolution of the represented plans. We accomplish this by *refining* hyperballs.

**Definition** **5** (Refinement)**.** *The refinement function refine takes in two arguments, a hyperball b and a scaling parameter a∈(0,1), and returns a set of new “denser” hyperballs:*

$$\mathrm{refine}\left(b,a\right)=\left\{b^{\left(1\right)},b^{\left(2\right)},...b^{\left(k\right)}\right\}$$

*with the following properties:*

*1.*  *$s\left(b^{\left(1\right)}\right)=s\left(b\right)$,*
*2.*  *$\forall b^{\prime }\in \mathrm{refine}\left(b,a\right),\epsilon \left(b^{\prime }\right)=a\;\cdot \;\epsilon \left(b\right)$,*
*3.*  *b⊂⋃refine(b,a).*

Refinement replaces one hyperball with a set of smaller hyperballs covering the same region (Figure 3D). For example, refining $b_{2}$ in $b_{s}=\left(b_{0},b_{1},b_{2},b_{3}\right)$ produces

$$B=\{\left(b_{0},b_{1},b_{2}^{\prime },b_{3}\right):b_{2}^{\prime }\in \mathrm{refine}\left(b_{2},a\right)\}$$

with

$$⟦b_{s}⟧\subseteq \underset{b^{\prime }\in B}{⋃}⟦b^{\prime }⟧.$$

Thus, refinement increases the resolution of the represented plans without excluding the original bundle. We define the reliability of a path as $r_{K}\left(b\right)=r_{K}^{\epsilon_{b}}\left(s_{b}\right)$. Robust paths with the same initial and terminal fields represent solutions to the same achievement class. If a bundle contains a robust plan, then repeated balanced refinement eventually produces a sufficiently precise path representing the same solvable problem, as shown formally in Appendix A.

Refinement alone is insufficient, however, because it cannot represent a plan with necessary waypoints lying outside the existing hyperballs. Searching increasingly fine variations inside the wrong region would never reach such a plan. The agent must also add hyperballs covering new regions of state space, thereby *extending* the scope of its behaviors.

**Definition** **6** (Extension)**.** *The extension function extend takes in three arguments, a hyperball b and two scaling parameters a>1 and c>1, and returns a new set of hyperballs covering “new” regions of S:*

$$\mathrm{extend}\left(b,a,c\right)=\left\{b^{\left(1\right)},b^{\left(2\right)},...b^{\left(k\right)}\right\}$$

*with the following properties:*

*1.*  *$\forall b^{\prime }\in \mathrm{extend}\left(b,a,c\right),\epsilon \left(b^{\prime }\right)=a\;\cdot \;\epsilon \left(b\right)$,*
*2.*  *$B_{c\;\cdot \;\epsilon (b)}\left(s\left(b\right)\right)\subset b\cup \left(⋃\mathrm{extend}(b,a,c)\right)$.*

Extension adds hyperballs covering new regions of state space, while refinement increases resolution within regions already covered (Figure 3E,F). We model the choice to apply one mutation function or the other with the *polymutator function* $\overset{⋄}{m}$, which when applied repeatedly to a finite set $B_{0}$ produces a sequence

$$B_{i}={\overset{⋄}{m}}^{i}\left(B_{0}\right),$$

where each $B_{i}$ remains finite. We call $\overset{⋄}{m}$ *fair* if neither extension nor refinement is permanently neglected, and *balanced* if every hyperball produced in the sequence is eventually selected for both extension and refinement.

Let $S_{i}=S\left(B_{i}\right)$ be the center points of the hyperballs at stage *i*. The family of plans generated over the complete sequence is

(14) $$S_{\overset{⋄}{m}}^{*}=\underset{i\in N}{⋃}S_{S_{i}}^{*}.$$

Fair extension increases the scope of this family, while balanced refinement increases its precision. Together, these operations give $S_{\overset{⋄}{m}}^{*}$ its central property, namely, that it is a rational set of behaviors.

**Theorem** **1** (The Polymutator Behavior Set is Rational)**.** If $\overset{⋄}{m}$ is a fair and balanced polymutator function and if $S_{\overset{⋄}{m}}^{*}={⋃}_{i=0}^{\infty }S_{S({\overset{⋄}{m}}^{i}\left(B_{0}\right))}^{*}$, then:

$$\begin{array}{ccccc} 1. & \left|\;S_{\overset{⋄}{m}}^{*}\right| & ={ℵ}_{0} & (S_{\overset{⋄}{m}}^{*}\;\mathrm{is}\;\mathrm{countably}\;\mathrm{infinite}) & (\mathrm{by}\;\mathrm{Lem}.\;\mathrm{A14}\;)\\ 2. & \bar{S_{\overset{⋄}{m}}^{*}} & =S_{S}^{*} & \left(S_{\overset{⋄}{m}}^{*}\;\mathrm{is}\;\mathrm{dense}\;\mathrm{in}\;S_{S}^{*}\;\right) & (\mathrm{By}\;\mathrm{Lem}.\;\mathrm{A15}\;) \end{array}$$

making $S_{\overset{⋄}{m}}^{*}$ a set of rational behaviors.

The detailed proof is given in Appendix A; here, we provide only the central intuition. Fairness ensures that both extension and refinement continue, while balance ensures that every hyperball is eventually selected for both operations. Consequently, extension eventually covers the waypoints of any finite plan, and refinement subsequently reaches any required precision. Formally,
  (15) $$\forall s\in S_{S}^{*},\;\forall \epsilon >0\;;\exists s^{\prime }\in S_{S_{\psi (B_{0},s,\epsilon )}}^{*}:s\in N_{\epsilon }\left(s^{\prime }\right).$$
  Thus, every plan can be approximated arbitrarily closely by a member of the countable family $S_{\overset{⋄}{m}}^{*}$.

The absence of external resets nevertheless constrains the order of this search: every attempted plan must begin where the agent currently is. Moreover, the agent only needs one robust representative of each achievement class, and can defer paths containing a failed sub-path while exploring other alternatives (Figure 3G). These considerations determine how the rational family must be organized and searched in practice.

The construction replaces the unmanageable trajectory space (with cardinality $2^{c}$) and the still-uncountable set of finite plans (with cardinality c) with the rational family $S_{\overset{⋄}{m}}^{*}$, which is countably infinite but dense in $S_{S}^{*}$. Countability makes systematic enumeration possible, while density ensures that any required robust plan can be approximated arbitrarily closely. The remaining question is how to organize this enumeration in practical terms.

## 4. Segraphs

A segraph records outcome information at a resolution between individual trajectories and whole plans. Each vertex represents a hyperball in state space, while each directed edge represents the bundle of 1-plans between two vertex fields together with evidence about its traversability. Therefore, it supplies the Behavioral Index Structure (BIX) described in Section 3: a pointer into behavior space that supports incrementation through that space. The following definition is intended to be sufficient rather than minimal.

**Definition** **7** (Segraph)**.** *A *segraph* is a tuple G=(S,V,E,Z,Λ) with:*

- *S: The state space approximated by the segraph.*
- *V: A finite set of vertices. Each $v_{i}=\left(v_{i},F_{i}\right)$ has an identifier $v_{i}$ and a field $F_{i}\subset S$, with measure $M_{i}=M\left(v_{i}\right)=\mu \left(F_{i}\right)$.*
- *E: A set of directed edges. Each $e_{i,j}=\left(\left(v_{i},v_{j}\right),\left(n_{s},n_{f}\right),n_{\Upsilon },\iota \right)$ connects $v_{i}$ to $v_{j}$ and represents the field $c_{i,j}=F_{i}\times F_{j}\subset S^{2}≅S_{S}^{1}$, where $\left(n_{s},n_{f}\right)\in N^{2}$ are successful and failed traversal counts, $n_{\Upsilon }\in N$ is a “failed to reach” count, and ι∈{−1,0,1} is manual inhibition. The edge-field measure is $M_{i,j}=M\left(e_{i,j}\right)=M_{i}M_{j}$.*
- *$Z=(s,v,e^{⋆},v^{⋆})$: The agent’s current state s, current vertex v, goal edge $e^{⋆}$, and goal vertex $v^{⋆}$.*
- *$\Lambda =(\alpha^{\prime },\ldots )$: Algorithm-specific state regulating which edges may be used for path construction, including the affordance threshold $\alpha^{\prime }\in R^{+}$.*

**Figure 4:**
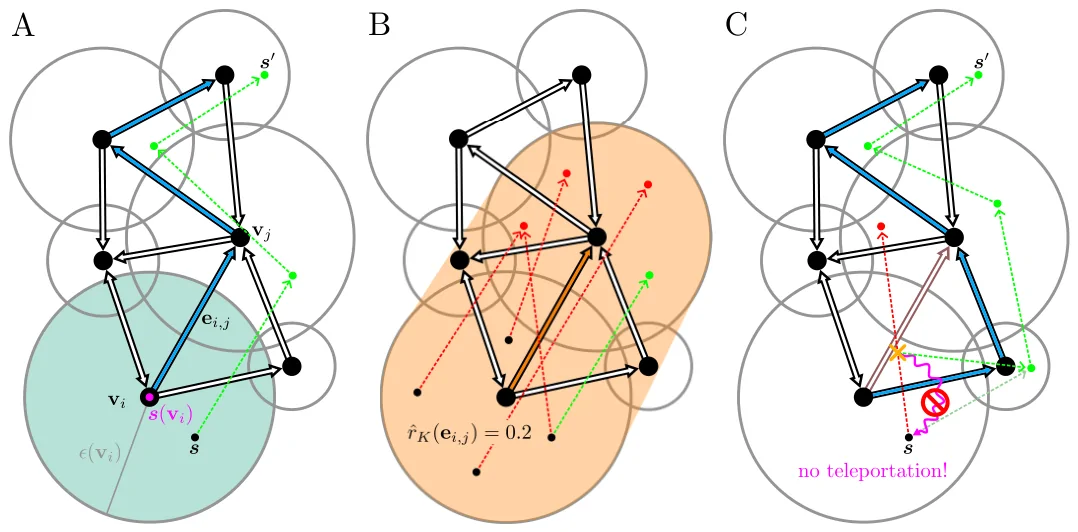
(**A**) A *segraph* is a directed graph with vertices that segment the state space. Each vertex’s segment of state space, or *field*, is a hyperball centered at $s(v_{i})$ with radius $\epsilon (v_{i})$. An edge represents a connection between two regions of state space. In order to help the agent reach a distant goal such as $s^{\prime }$, the segraph will find a path (sequence of vertices highlighted in blue) between the vertex the agent is currently in and a vertex containing the goal. Then, the agent will sample waypoints from the fields of the vertices along the path, generating a plan from its current state to the target state (green dots connected by dashed line). (**B**) Experience trying to cross the edges of the segraph allows the segraph to estimate the reliability of each of its edges. (**C**) Unreliable edges (such as the one marked in orange) can be *inhibited*, prevented from use in pathfinding, which can allow for potentially better plans to be tried (example alternative path highlighted in orange). The new plan that is generated has to start where the last (failed) plan left the agent, as there are no external resets.

*The edge values above support estimates of reliability, affordance, epistemic value, and refinement stress, defined below. The segraph’s coverage is $S\left(G\right)={⋃}_{v_{i}\in V}F_{i}$. A schematic illustration of a segraph and some of its properties is shown in Figure 4.*

The operations refine and extend apply directly to segraph vertices, giving $G^{\prime }=\mathrm{refine}\left(G,v\right)$ or $G^{\prime }=\mathrm{extend}\left(G,v\right)$. Two pragmatic additions are required. A redundancy constraint prevents the addition of a vertex for which the field is already closely represented, while a linking function link adds edges because the segraph is not assumed to be fully connected.

The segraph consequently has two coupled roles: it supplies paths to distant goals, and it regulates its own mutation using the outcomes of attempted paths. Repeated use enumerates paths over the current segraph, while repeated mutation changes which paths are available. Therefore, path outcomes guide segraph enumeration, and the resulting segraph guides path enumeration.

Adaptive use and mutation of the segraph both depend on remembering edge outcomes. The agent estimates the true reliability $r_{K}\left(e_{i,j}\right)$ from successful and failed traversal counts as

(16) $${\tilde{r}}_{K}\left(e_{i,j}\right)=\frac{n_{s}\left(e_{i,j}\right)+{\tilde{n}}_{s}}{n_{s}\left(e_{i,j}\right)+{\tilde{n}}_{s}+n_{f}\left(e_{i,j}\right)+{\tilde{n}}_{f}}=\frac{n_{s}\left(e_{i,j}\right)+{\tilde{n}}_{s}}{n\left(e_{i,j}\right)+{\tilde{n}}_{s}+{\tilde{n}}_{f}},$$

where $n\left(e\right)=n_{s}\left(e\right)+n_{f}\left(e\right)$ and $\left({\tilde{n}}_{s},{\tilde{n}}_{f}\right)$ are pseudo-counts encoding the initial reliability estimate. These counts subsequently influence segraph mutation, goal selection, and path selection.

### 4.1. Enumerating Paths

Because each plan must begin where the agent currently is, the principal choices available over a fixed segraph are the target and the path to that target. We model goal selection with a distribution g over V. A path $p=(v_{0},v_{1},\ldots ,v^{⋆})$ contains no repeated vertices and must begin at a vertex for which the field contains the current state. Let $P_{G}^{*}=P_{\left(V,E\right)}^{*}$ be the paths over G and let $P_{\left(V,E\right)}^{v\to v^{⋆}}$ model the selection among paths from v to $v^{⋆}$. This finite path set is a discretized proxy for $S_{S(G)}^{*}$ and may be enumerated in any order, although efficient problem solving requires a useful order.

Only one robust path is needed for each ordered pair of vertices. Let $P_{G}^{*}\left(v,v^{\prime }\right)=\left\{p\in P_{G}^{*}:p\left[0\right]=v,\;p\left[-1\right]=v^{\prime }\right\}$ be the paths connecting v to $v^{\prime }$ and let PG*+(v,v′)={p∈PG*(v,v′):rK(p)=1} be the robust paths among them. As in Section 2, the robust paths joining the same endpoints form an achievement class $\xi_{v,v^{\prime }}^{G}$, with $\Xi_{V}^{G}=\left\{\xi_{v,v^{\prime }}^{G}:v,v^{\prime }\in V\right\}$ as the set that must be enumerated. Since the agent does not know which paths are robust, it searches $P_{G}^{*}$ non-exhaustively by inhibiting different sets of edges.

### 4.2. Try and Try Again: Failure-Driven Enumeration

The most basic response to failure is to not repeat the failed behavior or sub-behavior. We implement this by inhibiting any edge that the agent fails to traverse. Suppose the agent begins at v, selects goal $v^{⋆}$, and attempts path p. We express the outcome as

(17) $$\mathrm{try}\left(G,p\right)=\left(s^{\prime },v^{\prime },E^{\times },E^{✓}\right),$$

where $s^{\prime }$ and $v^{\prime }$ are the resulting state and vertex, while $E^{\times }$ and $E^{✓}$ are the edges that failed and succeeded during the attempt. On success, $v^{\prime }=v^{⋆}$ and $E^{\times }=⌀$; otherwise, $v^{\prime }\neq v^{⋆}$ and $E^{\times }\neq ⌀$.

The objective is to enumerate achievement classes rather than every path. If p fails, then $r_{K}\left(p\right)<1$ and it cannot represent $\xi_{v,v^{⋆}}^{G}$. Moreover, under the deterministic and noise-free assumptions used here, no path containing a failed edge can be robust. Such paths can be provisionally excluded from enumeration over the current segraph.

We partition E into inhibited edges $E^{↓}$ and active edges $E^{+}$. Initially, $E^{+}=E$ and $E^{↓}=⌀$. After each call to try, $E^{↓}\leftarrow E^{↓}\cup E^{\times }$ and $E^{+}\leftarrow E^{+}-E^{\times }$. The agent then samples from $P_{\left(V,E^{+}\right)}^{v\to v^{⋆}}$, so the next attempt begins from its new location while excluding the failed transition.

### 4.3. The Curse of Locality

**Figure 5:**
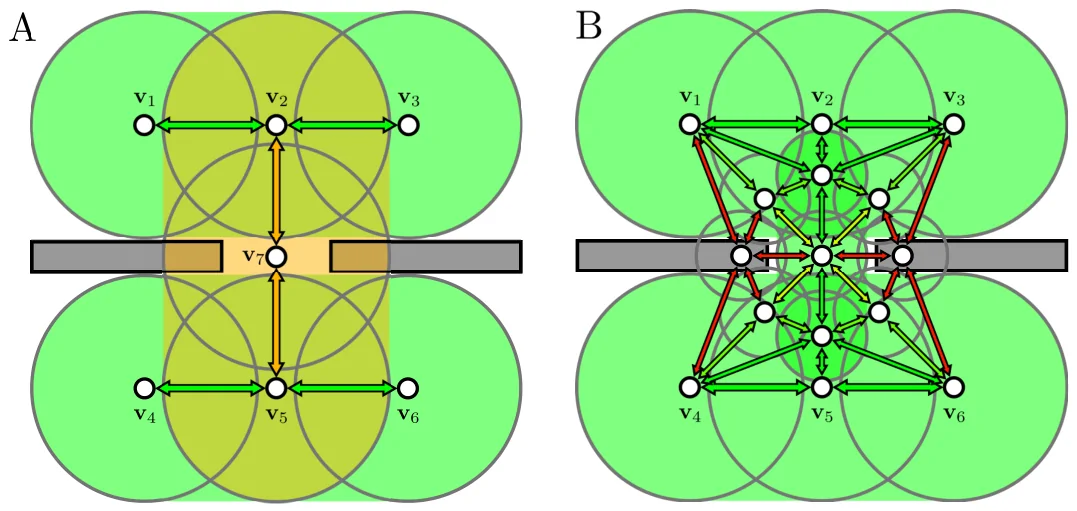
(**A**) For the shown segraph G, in the event that the unreliable orange edges fail and are inhibited, the elements of $\Xi_{V}^{G}$ will not be connected to each other. This means there is no guarantee that our agent can actually enumerate $\Xi_{V}^{G}$, complicating the agent’s task of trying every behavior. (**B**) However, through systematic refinement, it can be possible to construct a new segraph $G^{\prime }$ which connects the elements of G, allowing the agent to enumerate $\Xi_{V}^{G}$ using a *different* segraph.

Failure-driven inhibition can disconnect the segraph before its achievement classes have been enumerated. In Figure 5A, every edge within $V^{\prime }=\left\{v_{1},v_{2},v_{3}\right\}$ and within $V^{\prime \prime }=\left\{v_{4},v_{5},v_{6}\right\}$ is robust, but the connecting edges through $v_{7}$ are not. Thus, the achievement set contains classes internal to $V^{\prime }$ and $V^{\prime \prime }$, but none joining the two sets. An attempted path from $v_{1}$ to $v_{4}$ could fail at $e_{2,7}$ or $e_{7,5}$; inhibiting that edge disconnects the current segraph and prevents the agent from enumerating both groups of achievement classes from its present location.

This does not prevent the achievement from becoming accessible through a later segraph. Let $S_{G}^{*}=\{s\in p:p\in P_{G}^{*}\}$ be the set of plans sampled from paths over G. We say that $G^{\prime }$ *contains* G, written $G^{\prime }⋗G$, when $S_{G}^{*}\subset S_{G^{\prime }}^{*}$. Each ideal mutation mutation∈{extend,refine,link} is containment-preserving, so mutation(G)⋗G. By transitivity, a mutation sequence from $G_{i}$ to $G_{i+k}$ gives $\Xi_{S_{K}}^{G_{i}}\subset \Xi_{S_{K}}^{G_{i+k}}$. Consequently, achievement classes that cannot be enumerated while $G_{i}$ is disconnected will remain represented within a later segraph with new vertices and edges that make them accessible (Figure 5B).

Mutation must occur where additional structure is needed. Since mutation occurs around the agent’s current location, balanced construction depends partly on controlling where the agent travels. Segraph mutation determines which paths are available, while path selection determines where subsequent mutation can occur; consequently, the two mechanisms regulate each other.

## 5. Segraph Self-Organization

In this section, we lay out one possible way of arranging the self-organized usage and mutation of a segraph. The particular mechanisms were guided by intuition and experimentation, and should be considered illustrative rather than definitive. The objective is to regulate path selection and mutation so as to increase $\mu (\Xi_{S_{K}}^{A})$, that is, the measure of the set of points in $S_{K}$ that the agent can go to and from, while retaining the balanced extension and refinement required by the theoretical construction.

The ability of the agent to go from one point to another corresponds to an *affordance* [14]. A path p represents a bundle of plans, with $⟦p⟧={✕}_{v\in p}b(v)$, and its affordance can be expressed as $\alpha \left(p\right)=r_{K}\left(p\right)\;\cdot \;M\left(p\left[0\right]\right)\;\cdot \;M\left(p\left[-1\right]\right)$. Therefore, given a distribution over paths, the affordance of the agent is $\alpha \left(A\right)=\sum_{p\in P_{G}}P\left(p\right)\alpha \left(p\right)$. The following subsections consider how segraph mutation affects this quantity and how corresponding estimates can be used to order paths and regulate mutation.

### 5.1. Refinement

Although refinement acts on a vertex, the evidence indicating when refinement may be useful is stored on an edge, because the objective is to increase the resolution of the corresponding bundle of 1-plans. We use *refining an edge* to mean refining one of its vertices. Let

$$q\left(e\right)=\sum_{p\in P_{G}\left(e\right)}P\left(p\right)\alpha \left(p\right)$$

be the estimated global utility associated with paths passing through e. If refinement replaces e with child edges $\left\{e_{1}^{\prime },\ldots ,e_{k}^{\prime }\right\}$, then $q_{\mathrm{ref}}\left(e\right)=\sum_{i=1}^{k}q\left(e_{i}^{\prime }\right)$ and $\Delta q\left(e\right)=q_{\mathrm{ref}}\left(e\right)-q\left(e\right)$. Under the simplifying assumptions detailed in Appendix B.3, this becomes

(18) $$\Delta q\left(e\right)=c\;\cdot \;N_{p}\left(e\right)r_{K}\left(e\right)\left(1-r_{K}\left(e\right)\right),$$

where $N_{p}\left(e\right)$ is the weighted number of paths passing through the edge. This expression favors refinement of edges that are both frequently used and of intermediate reliability. Under the simplified derivation, unused, completely reliable, and completely unreliable edges receive zero score.

The agent does not know Δq(e) and instead uses the empirical stress score

(19) $$\Delta \tilde{q}\left(e\right)=c\;\cdot \;n\left(e\right){{\tilde{r}}_{K}}^{\prime }\left(e\right)\left(1-{{\tilde{r}}_{K}}^{\prime }\left(e\right)\right),$$

where *c* is an unknown environment-dependent constant, n(e) estimates usage, and ${{\tilde{r}}_{K}}^{\prime }\left(e\right)$ estimates reliability without pseudo-counts. Because traversal success is binary, it can be treated as a Bernoulli random variable with empirical success probability ${\hat{p}}_{e}={{\tilde{r}}_{K}}^{\prime }\left(e\right)$. Its binary Shannon entropy is

$$H\left(e\right)=-{\hat{p}}_{e}\log{\hat{p}}_{e}-\left(1-{\hat{p}}_{e}\right)\log\left(1-{\hat{p}}_{e}\right).$$

Both H(e) and the Bernoulli variance ${\hat{p}}_{e}\left(1-{\hat{p}}_{e}\right)$ are maximized at ${\hat{p}}_{e}=1/2$ and minimized at 0 and 1. Thus, the stress score prioritizes high-usage edges with high outcome uncertainty, although it is not an exact calculation of the expected information gain.

Because ${\hat{p}}_{e}=n_{s}\left(e\right)/n\left(e\right)$, the empirical score for n(e)>0 can equivalently be written as

(20) $$\Delta \tilde{q}\left(e\right)=c\;\cdot \;n_{f}\left(e\right){\hat{p}}_{e}.$$

Refinement is evaluated following a failed traversal, so $n_{f}\left(e\right)$ represents accumulated pressure to refine, while ${\hat{p}}_{e}$ discounts edges that have never demonstrated successful traversal. This can be interpreted as a rough proxy for failure pressure multiplied by continued path usage probability. Nevertheless, its actual utility depends on the agent’s location, global segraph topology, path selection probabilities, affordance thresholding, and the properties of the child edges produced by refinement.

### 5.2. Ordering Paths by Affordance to Regulate Refinement

Directly ordering paths by their contribution to α(A) is intractable, so we instead use the affordance of individual edges,

$$\alpha \left(e\right)=r_{K}\left(e\right)M\left(e\right),\;\tilde{\alpha }\left(e\right)={\tilde{r}}_{K}\left(e\right)M\left(e\right).$$

Thresholding edges rather than complete paths is an approximation, but biases pathfinding toward regions of the plan space that are large and empirically traversable, increasing the probability that the agent encounters edges for which refinement may be useful.

We introduce a dynamic affordance threshold $\alpha^{\prime }$ and partition the edges into

$$E^{<}\left(\alpha^{\prime }\right)=\left\{e\in E:\tilde{\alpha }\left(e\right)<\alpha^{\prime }\right\},\;E^{\geq }\left(\alpha^{\prime }\right)=\left\{e\in E:\tilde{\alpha }\left(e\right)\geq \alpha^{\prime }\right\}.$$

Combining threshold inhibition with the failed edges $E^{↓}$ gives

$$E^{-}=E^{<}\left(\alpha^{\prime }\right)\cup E^{↓},\;E^{+}=E^{\geq }\left(\alpha^{\prime }\right)-E^{↓}.$$

Pathfinding is restricted to $E^{+}$. Starting with a high threshold prioritizes higher-affordance edges; if no path can be found, lowering $\alpha^{\prime }$ progressively reintroduces alternatives.

High affordance is not identical to high reliability, since the edge measure also contributes to the score. The primary purpose of the threshold is not simply to find reliable paths but to regulate where refinement occurs. Since mutation happens where the agent travels, thresholding preferentially exposes edges that are either large and reliable (useful for navigation) or large and imperfectly reliable (plausible candidates for refinement). However, lowering $\alpha^{\prime }$ cannot restore a path when inhibition of a failed edge has already disconnected the active graph, requiring an additional feedback mechanism.

### 5.3. Usage and Mutation Feedback-Loop

Affordance thresholding and failure-driven inhibition must both be reversible, otherwise a failed edge could remain permanently unavailable and never be revisited or refined, while an edge reintroduced by lowering $\alpha^{\prime }$ could never again be excluded. ARMS lowers $\alpha^{\prime }$ and clears $E^{↓}$ whenever no path can be found, resetting failure-driven inhibition. Conversely, every traversal failure raises $\alpha^{\prime }$, temporarily discouraging other low-affordance edges.

This feedback makes the search adaptive, but also means that ARMS does not perform a literal exhaustive enumeration of the paths over any fixed segraph. Raising the threshold can inhibit an untried reliable edge, while clearing inhibition permits a previously failed edge to be tried again. Refinement partly counteracts this masking effect. Suppose that a robust solution requires an edge with affordance $\alpha_{\min}$, but other imperfect edges with affordance greater than $\alpha_{\min}$ repeatedly fail and raise the threshold above it. Repeated experience on an imperfect masking edge eventually triggers refinement. Unless its child edges are sufficiently reliable to remain useful without repeatedly raising the threshold, they will have smaller fields and lower affordance. Under the simplifying assumptions used here, continued refinement causes masking child edges to either fall below $\alpha_{\min}$ or become reliably traversable.

Consequently, a later refined segraph may expose paths representing achievement classes that were inaccessible in an earlier segraph. This provides the intended coupling between path enumeration and segraph mutation: path outcomes determine where mutation occurs, while mutation changes which paths can subsequently be attempted. The argument motivates the feedback mechanism but does not establish a completeness guarantee for finite-memory ARMS, for which the threshold adaptation and refinement schedule remain heuristic.

### 5.4. Extension and Linking

The ideal balanced construction eventually extends and refines every relevant vertex. ARMS approximates this requirement by extending a vertex after it has been successfully visited. Some unvisited vertices may be refined before being extended, but if one of their child vertices is subsequently reached, then that child can itself be extended. This concentrates graph growth around regions demonstrated to be accessible, although it does not strictly guarantee balanced extension.

Linking is likewise heuristic. Vertices with overlapping fields are linked automatically, and when a transition from v toward $v^{\prime }$ passes through another vertex $v^{\prime \prime }$, ARMS adds an edge from v to $v^{\prime \prime }$. This increases connectivity using locally collected experience without fully connecting the segraph.

### 5.5. Ordering by Epistemic Value

To direct exploration toward large and under-sampled regions of plan space, we assign each edge the size-normalized count-based epistemic score

(21) $$\Upsilon \left(e\right)=\frac{M(e)}{n\left(e\right)+n_{\Upsilon }\left(e\right)+1},$$

where $n\left(e\right)=n_{s}\left(e\right)+n_{f}\left(e\right)$ and $n_{\Upsilon }\left(e\right)$ adjusts the score independently of actual traversal counts. Large edges represent more potential 1-plans, while less-traversed edges have less certain reliability estimates. In this way, ARMS selects a goal edge $e^{⋆}$ rather than merely a goal vertex.

Let $P_{G}^{*}v,e^{⋆}$ be the set of paths beginning at v and ending by crossing $e^{⋆}$. If $e^{⋆}=\left(v^{\prime },v^{⋆}\right)$, then

$$P_{G}^{*}v,e^{⋆}=\left\{p+v^{⋆}:p\in P_{G}^{*}v,v^{\prime }\right\}.$$

We model goal selection with a distribution $g_{\Upsilon }$ that increases with Υ(e). Attempting to traverse an edge increments n(e) and lowers its epistemic score, reducing the probability of immediate reselection. Under suitable ergodic assumptions, this discourages permanent neglect of an edge, although attempting to visit every edge is not equivalent to trying every path.

Likewise, let $P_{G}^{v\to e^{⋆}}$ be a distribution over paths to the selected goal edge. Uniform sampling from this distribution would provide a trivial eventual enumeration of the finite path set, whereas ARMS additionally biases path selection using affordance and simplicity.

### 5.6. Ordering by Simplicity

All things being equal, shorter paths are preferable. Let $k_{\min}$ be the minimum length for which an active path from v to e exists. We restrict the path distribution so that

(22) $$P_{\left(V,E^{+}\right)}^{v\to e}\left(p\right)=\left\{\begin{array}{cc} 0 & \mathrm{if}\;|p|>k_{\min},\\ p(p) & \mathrm{otherwise},\;\mathrm{with}\;p(p)>0. \end{array}\right.$$

If a shortest path succeeds, then a representative of $\xi_{v,e}^{G}$ has been found and longer alternatives are unnecessary. If the shortest paths fail, failure-driven inhibition removes their failed edges from the active graph. Once no path of length $k_{\min}$ remains, the minimum available length increases automatically. Selecting shortest paths together with failure-driven inhibition orders attempted paths by length without requiring the agent to store an explicit enumeration.

## 6. Adaptive Real-Time Metasearch over Segraphs

### 6.1. Algorithm

ARMS is a finite real-time heuristic realization of the feedback loop developed above: traversal experience changes both path selection and segraph mutation, so that search over paths drives and is driven by search over segraphs. Rather than requiring a fixed task goal, ARMS selects exploratory goal edges so that the resulting segraph can later support goals specified on demand. This coupled search gives the Adaptive Real-Time Metasearch over Segraphs (ARMS) its name.

For ARMS, $\Lambda =(\alpha^{\prime },\alpha_{0}^{\prime },\Delta^{+},\Delta^{-},\kappa )$ contains the current and initial affordance thresholds, the rates at which the threshold is raised and lowered, and the number of consecutive pathfinding failures. An edge is available when its estimated affordance exceeds $\alpha^{\prime }$, unless ι(e) forces it to be inhibited or disinhibited. Raising $\alpha^{\prime }$ excludes lower-affordance edges, while lowering it reintroduces them. Following a failed traversal, $p_{\mathrm{persist}}$ controls whether the agent continues to pursue the current goal.

At the beginning of each search cycle, ARMS clears manual inhibition, selects the edge with the highest epistemic score as its goal, forces that edge to be disinhibited, and searches for a path through it. The agent samples a target point from each successive vertex field and passes it to K. A successful traversal increments the edge’s success count, updates the current vertex, and extends the vertex reached. A failed traversal increments the failure count, locates the agent in the segraph, and inhibits the failed edge. Sufficient stress additionally triggers refinement; the affordance threshold is raised, after which the agent either searches for another path to the same goal or selects a new goal.

**Figure 6:**
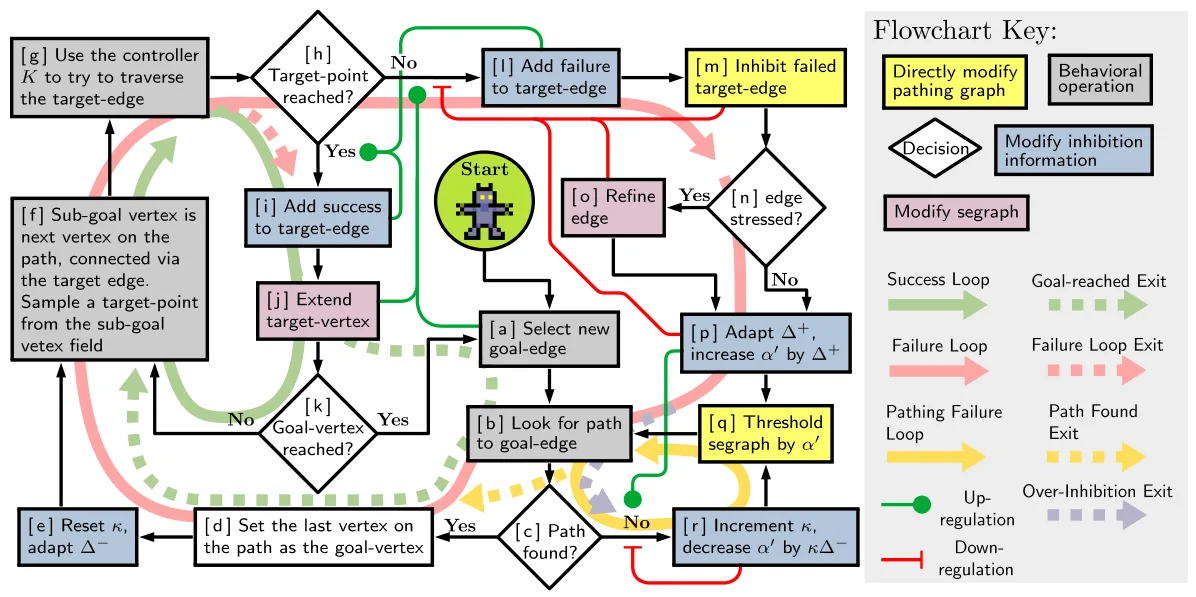
The search algorithm can be decomposed into three major interconnected loops. The first is the “success” loop (solid green arrow [f, g, h, i, j, k]). Staying on this loop results in reaching the selected goal, triggering the selection of a new goal and ideally leading back to the success loop (dashed green arrow). However, selection of ambitious goals and mismatch between $G^{⋆}$ and the environment will usually lead to failed edge traversals, triggering the “failure” loop (solid red arrow, [f, g, h, l, m, n, o (optionally), p, q, b, c, d, e]). Both the success and failure loops update the reliability estimates of edges, allowing the agent to more easily distinguish good from bad edges, which over time promotes the success loop. Repeated failure raises $\alpha^{\prime }$ and can result in a pathing failure (over-inhibition exit). Each pathing failure lowers $\alpha^{\prime }$, so the “pathing failure” loop (yellow arrow) is self-inhibiting and will eventually lead back to the success or failure loops.

If no path can be found, ARMS increments the goal edge’s failed-to-reach count, clears manual inhibition, and lowers $\alpha^{\prime }$ until another path becomes available. These success, traversal failure, and pathfinding failure loops are shown in Figure 6; the precise threshold adaptation functions are given in Appendix B.2, and Algorithm 1 gives the complete procedure.
**Algorithm 1** Adaptive Real-Time Metasearch over Segraphs  1:Z←(s,v,·,·) # Initialize agent state with undefined goals  2:$\Lambda \leftarrow (\alpha^{\prime },\alpha_{0}^{\prime },\Delta^{+},\Delta^{-},\kappa )$ # Initialize inhibition-regulation information  3:G←(S,V,E,Z,Λ)  # Initialize segraph  4:**while** true **do**  5:      ∀e∈E,ι(e)←0 # Clear all manual inhibition  6:      $e^{⋆}\leftarrow {\mathrm{argmax}}_{e\in E}\Upsilon \left(e\right)$ # Select a new goal edge  7:      $\iota (e^{⋆})\leftarrow 1$ # Force the goal edge to be disinhibited  8:      $v^{⋆}\leftarrow \mathrm{dst}\left(e^{⋆}\right)$ # Set the goal vertex as the destination vertex of the goal edge  9:      p←⌀ # No path has yet been found10:      r←0 # The goal-edge traversal has not yet succeeded11:      **while** $\neg (v=v^{⋆}\wedge r=1)$ **do** # Continue until the goal edge is traversed successfully12:            **while** p=⌀ **do** # Continue until a path is found13:                   $E^{+}\leftarrow \left\{e\in E:\left(\tilde{\alpha }\left(e\right)\geq \alpha^{\prime }\vee \iota \left(e\right)=1\right)\wedge \iota \left(e\right)\neq -1\right\}$ # Active edges14:                   $p\leftarrow P_{\;⊚}\;\left(v,e^{⋆},\left(V,E^{+}\right)\right)$ # Look for a path to the goal edge15:                   **if** p=⌀ **then** # No path was found16:                          $n_{\Upsilon }\left(e^{⋆}\right)\leftarrow n_{\Upsilon }\left(e^{⋆}\right)+1$ # Penalize the unreachable goal edge17:                          $e^{-}\leftarrow {\mathrm{argmax}}_{e\in E\setminus E^{+}}\tilde{\alpha }\left(e\right)$ # Find the highest-affordance inhibited edge18:                          ∀e∈E,ι(e)←0 # Clear all manual inhibition19:                          $\Lambda \leftarrow f_{3}^{\Lambda }\left(\Lambda ,e^{-}\right)$ # Increment κ and lower $\alpha^{\prime }$20:            $\Lambda \leftarrow f_{2}^{\Lambda }\left(\Lambda \right)$ # Reset κ and adapt $\Delta^{-}$21:            **while** p≠⌀ **do** # Traverse the remaining path22:                   $v^{▹}\leftarrow p.\mathrm{pop}\_\mathrm{front}\left(\right)$ # Get the next subgoal vertex23:                   $e^{▹}\leftarrow \mathrm{edge}\left(v,v^{▹}\right)$ # Retrieve the corresponding segraph edge24:                   $s^{▹}∼U\;\left(F(v^{▹})\right)$ # Sample a target point from the subgoal field25:                   $\left(s,r\right)\leftarrow \left(t_{K}^{\left(s,s^{▹}\right)}\left(T_{s,s^{▹}}^{K}\right),\;r_{K}\left(s,s^{▹}\right)\right)$ # Use the controller to attempt the traversal26:                   **if** r=1 **then** # The target point was reached27:                          $n_{s}\left(e^{▹}\right)\leftarrow n_{s}\left(e^{▹}\right)+1$ # Record the successful traversal28:                          $v\leftarrow v^{▹}$ # Update the agent’s current vertex29:                          G←extend(G,v) # Extend the reached vertex30:                   **else** # The controller failed to reach the target point31:                          $n_{f}\left(e^{▹}\right)\leftarrow n_{f}\left(e^{▹}\right)+1$ # Record the failed traversal32:                          v←locate(s,V) # Recalculate the agent’s current vertex33:                          $\iota (e^{▹})\leftarrow -1$ # Manually inhibit the failed edge34:                          **if** $\Delta \tilde{q}\left(e^{▹}\right)>1$ **then** # The failed edge is stressed35:                                  $G\leftarrow \mathrm{refine}(G,e^{▹})$ # Refine the failed edge36:                          $\Lambda \leftarrow f_{1}^{\Lambda }\left(\Lambda ,e^{▹}\right)$ # Adapt $\Delta^{+}$ and raise $\alpha^{\prime }$
37:                          p←⌀ # Discard the invalidated path38:                          u∼U([0,1])39:                          **if** $u>p_{\mathrm{persist}}$ **then**40:                                  **break to Line 5** # Abandon the current goal41:                          **break** # Look for another path to the goal edge

The theoretical construction establishes that a countably infinite dense family of plans can be enumerated, but does not prescribe a unique practical enumeration or prove that a particular finite algorithm performs it; therefore, ARMS should be judged as one heuristic approximation to the functional requirements of the construction. It is neither a unique solution nor an implementation that inherits the hyperadaptability guarantee.

### 6.2. ARMS Design Motivation

The low-level controller’s inability to reach every target directly motivates decomposing $S^{2}$ into overlapping edge fields that can be sequentially composed. The segraph makes these regions indexable: vertex fields represent regions of *S*, edge fields represent regions of $S^{2}$, and paths specify bundles of plans that the controller can attempt. The ideal construction requires extension to expand coverage and refinement so as to increase resolution under a fair and balanced schedule that does not permanently neglect any region needed for a robust solution.

ARMS approximates these requirements using several coupled heuristics. Extension follows successful visitation, concentrating graph growth around regions that the agent has demonstrated it can reach. The stress score concentrates refinement on used edges with uncertain outcomes, rather than indiscriminately refining perfectly reliable or completely unsuccessful edges. The epistemic score favors large under-sampled goal edges, while shortest-path selection favors simpler plans and the affordance threshold favors edges that are large and empirically reliable. Because the threshold can fall after pathfinding failure, lower-affordance alternatives can subsequently be reconsidered. Together, these mechanisms distribute search and representational capacity across the plan space without explicitly storing an enumeration.

None of these choices is forced by the theoretical construction. Other mechanisms could perform the same functional roles, and ARMS is not claimed to be a unique or optimal realization of behavioral search. Moreover, its finite memory and heuristic mutation schedule mean that it does not inherit the theoretical guarantee of hyperadaptability. The intended role of each mechanism and the empirical evidence provided for it are discussed in the Discussion. An operation-level complexity analysis appears in Appendix D, and statistical validation and runtimes are provided in Appendix C.

## 7. Results

Initial development of the ARMS algorithm occurred in 2D mazes, since this allowed for easy visual inspection and intuitive understanding. We began by testing the ability of the ARMS algorithm in its naive, astute, and misled variants to learn to navigate around a fixed set of regular rectangular and irregular polygonal mazes. To measure the agent’s ability in a way that was irrespective of maze size, we calculate the agent’s segraphs’ *reliability* R(G), which is a distance-weighted average of the reliability of the graph for navigating between pairs of points sampled from the state space, allowing the agent to re-path if necessary to reflect the operation of the ARMS algorithm (see Appendix B.7). Here, R(G)=0 means complete unreliability, while R(G)=1 means total reliability. R(G) can be thought of as a variant of the affordance of the graph α(G) normalized to distance and environment size. We also checked the resilience of ARMS to shifts in the environment, its sensitivity to the initial field size guess, and its ability to handle higher-dimensional state spaces. Finally, we tested ARMS in a dynamic environment with rewards. The rationale and limitations of the maze, MountainCar, and computational cost metrics are summarized in Table A1.

These environments and the RL baseline were not selected in order to demonstrate the superiority of the ARMS algorithm or the behavioral search paradigm; instead, these experiments are meant solely to demonstrate the basic feasibility of behavioral search as imperfectly and approximately manifested in the ARMS algorithm, with individual environments testing distinct properties rather than constituting a comprehensive benchmark. In particular, comparison against a single RL method should not be treated as a comparison against goal-conditioned, archive-based, intrinsically motivated goal exploration, novelty search, or sampling-based methods.

The experiments also have different roles in evaluating the internal mechanisms of ARMS. The Naive, Astute, and Misled conditions manipulate the information used to bias vertex admission during extension; the initial field scale and MountainCar parameter experiments are sensitivity analyses; and the original stress score versus scaled failure count comparison is a controlled ablation of the uncertainty term in the refinement criterion. These interventions do not constitute a full factorial ablation of the core ARMS mechanisms. Overall task performance should be attributed to the integrated algorithm except where a controlled comparison directly isolates a specific mechanism.

### 7.1. 2D Mazes

**Figure 7:**
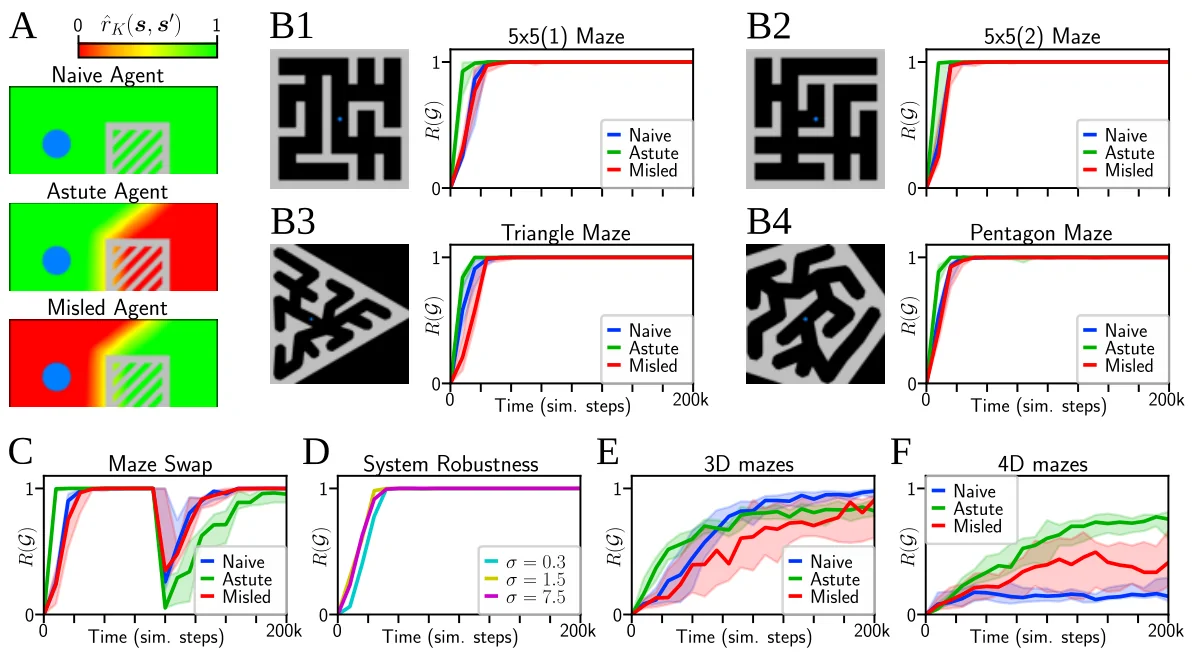
(**A**) Agents can have different knowledge of spatial navigability, either being Naive, Astute, or Misled. (**B**) Naive, Astute, and Misled agents’ ability to construct a reliable segraph in different maze environments. (**C**) Naive, Astute, and Misled agents’ ability to recover when the maze is changed (at 100k sim. steps). (**D**) Sensitivity of the Naive agent to a 25-fold variation in the initial field scale $\sigma_{0}$ in the 5 × 5(1) maze. (**E**) Naive, Astute, and Misled agents in random 3D mazes. (**F**) Naive, Astute, and Misled agents in random 4D mazes. Lines and shaded regions show medians and interquartile ranges. The 2D maze, maze-swap, and sensitivity conditions use n=8 completed trials per condition, while all 3D and 4D conditions use n=16 completed trials per condition. Corresponding endpoint, time-resolved, mean/SD, and confidence interval analyses can be found in Appendix C.

To build mazes, we generated randomly-connected 25-node graphs, converting the graph into a maze by (1) finding a random spanning tree of the graph and (2) converting the topology of the spanning tree into a continuous geometric environment with barriers. Two 5 × 5 mazes (5 × 5(1) and 5 × 5(2)), a triangular maze, and a pentagonal maze were randomly generated (see Appendix B.6) and used to determine if (1) the ARMS algorithm could successfully learn to navigate around these mazes and (2) whether incorporating prior knowledge about environment navigability could influence the ability of the ARMS algorithm to learn these mazes (Figure 7A). We ran eight simulations for each condition (maze × prior knowledge) for 200,000 timesteps, measuring R(G) every 10,000 timesteps (because the measurement is quite computationally expensive to make). The median (solid line) and interquartile range (shaded area) are shown for each condition in Figure 7. Across all conditions, ARMS achieves nearly perfect navigation ability within approximately 30,000 timesteps, and as the first four rows of Table 1 show, by the end of each simulation every prior-knowledge condition in all four fixed mazes had a final median R(G)=1.000 across all mazes and all prior knowledge conditions. At the beginning, it appears that having some accurate prior knowledge does speed up learning; surprisingly, however, misleading prior knowledge only seems to have a negative effect compared to the naive agent in the triangular maze, suggesting that our approach of adding prior knowledge to ARMS is conceptually shallow and does not correspond to the kinds of prior knowledge that we might expect animals or humans to have.

Consistent with this apparent early effect, a time-resolved Kruskal–Wallis analysis found a figure-wide corrected omnibus difference at 10,000 timesteps in the 5 × 5(1) maze ($\epsilon^{2}=0.648$, $p_{\mathrm{FWER}}=0.00704965$). Consistent with the absence of a final-time ordering by prior-knowledge condition, no final-time omnibus comparison in the four fixed 2D mazes survived figure-wide Holm correction (Appendix C).

Next, we were interested in the ability of the ARMS algorithm to recover from major environmental perturbations. To simulate this, we started Naive, Astute, and Misled agents inside of the 5 × 5(1) maze, then swapped at 100,000 timesteps to the 5 × 5(2) maze and continued the simulation for another 100,000 timesteps. As before, we measured R(G) every 10,000 timesteps, ran eight trials per condition, and plotted the median and interquartile range (Figure 7C). It seems that prior knowledge (the astute agent) speeds up early convergence, but it appears that this initial strength comes at a cost: the Astute agent recovers from the perturbation more slowly than the Naive one (which recovered to a final median R(G)=1.000) or the Misled one (which recovered to a final median of 0.999); see Table 1. Time-resolved omnibus differences survived figure-wide family-wise correction at 10,000–20,000 timesteps and again at 110,000 timesteps, shortly after the maze swap (Appendix C).

The initial field scale $\sigma_{0}$ controls the coarseness (inverse resolution) of the agent’s initial decomposition of the state space. An inappropriately small $\sigma_{0}$ will result in over-allocation of representational capacity on unnecessary detail, while an inappropriately large $\sigma_{0}$ will be insufficiently detailed to represent the genuine environmental topology. As the agent is assumed to be naive, we cannot rely on picking a “good” $\sigma_{0}$ for the environment. Therefore, we performed a sensitivity analysis on $\sigma_{0}$ for a Naive agent in the 5 × 5(1) maze using $\sigma_{0}\in \left\{0.3,1.5,7.5\right\}$, a 25-fold range, with eight independent trials per condition run for 200,000 timesteps. As can be seen in Figure 7D, despite initial differences in navigation ability, all conditions quickly converge to nearly perfect R(G) within about 40,000 timesteps. This demonstrates performance stability with respect to initial field scale in this particular 2D maze. However, because only one parameter was varied in a single environment, this should not be interpreted as evidence that ARMS is generally insensitive to parameter variation.

### 7.2. Higher-Dimensional Mazes

We also wanted to see if the ARMS algorithm could work in higher-dimensional state spaces. To test this, we generated random 3D and 4D mazes composed of overlapping capsules to make collision-checking simple, with 25-node maze topologies intended to be comparable to those of the 2D mazes we initially tested the ARMS algorithm on. However, matching the nominal number of maze nodes does not make geometric volume, sampling difficulty, or representational complexity equivalent across dimensions. Again, we simulated Naive, Astute, and Misled ARMS agents for 200,000 timesteps, though this time we ran 16 independent trials per condition due to the high variability induced by randomizing mazes per-trial. An additional difference is that due to the computational cost of collision-checking in these mazes, we allowed the agent to “jump” towards its target as opposed to actually numerically integrating the physics of the controller, stopping at any obstacles along the way. This makes the effective time scales of the physics somewhat faster compared to the 2D mazes.

In the shown 25-node 3D and 4D mazes, performance varied strongly with both dimensionality and prior-knowledge condition, and the ordering of the conditions was not consistent across dimensions. At the final measurement in 3D, the Naive agent had the highest median R(G), followed by the Misled and Astute agents. The final-time omnibus difference survived correction across all eight Figure 7 panels (H=15.16, $\epsilon^{2}=0.292$, $p_{\mathrm{Holm}}=0.00358$), although only the Naive–Astute pairwise comparison survived correction across all 24 pairwise comparisons (U=231, $r_{\mathrm{rb}}=0.805$, $p_{\mathrm{Holm}}=0.00257$). In 4D, the observed ordering was reversed, the Astute agent performed relatively well, the Naive agent had substantially lower performance, and the Misled agent split the difference. The final-time omnibus difference again survived correction (H=21.71, $\epsilon^{2}=0.438$, $p_{\mathrm{Holm}}=0.000154$), although only the Naive–Astute pairwise comparison survived figure-wide correction (U=19, $r_{\mathrm{rb}}=-0.852$, $p_{\mathrm{Holm}}=0.00104$). Time-resolved omnibus differences survived figure-wide correction intermittently in 3D and continuously from 60,000 through 200,000 timesteps in 4D (Appendix C).

**Figure 8:**
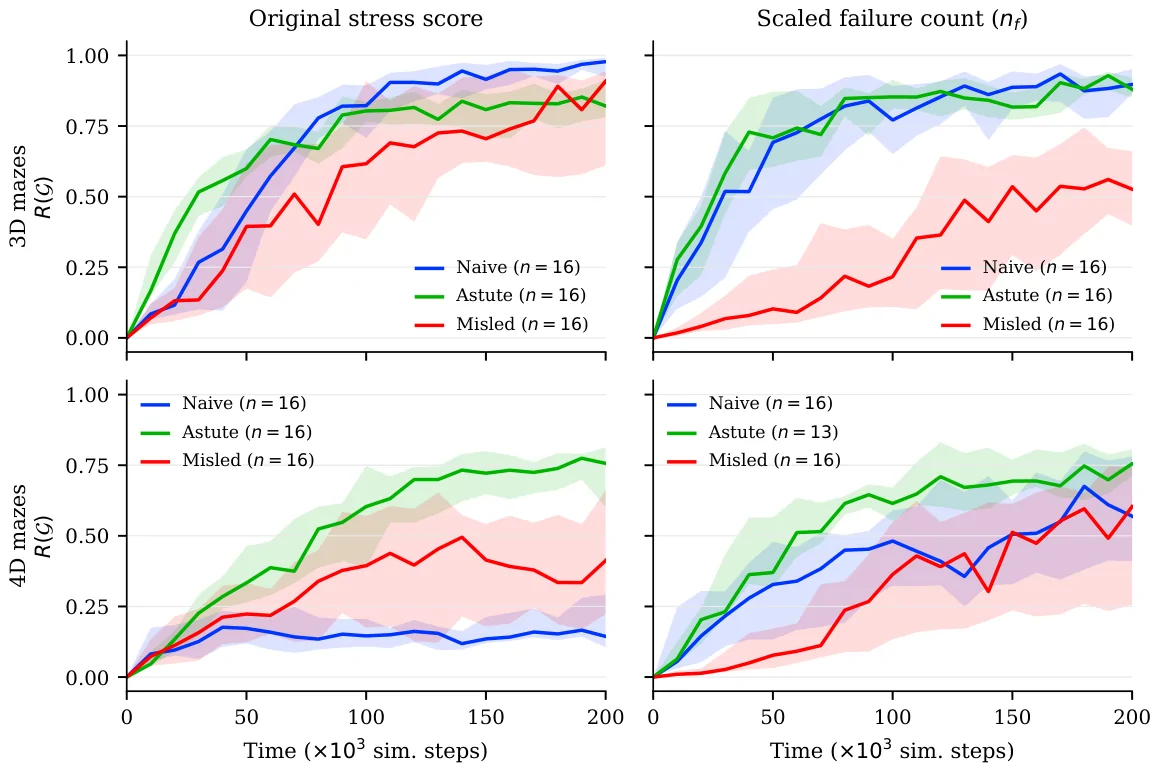
Controlled comparison of the original uncertainty-weighted stress score and a scaled failure-count refinement criterion in 3D and 4D mazes. Lines and shaded regions show medians and interquartile ranges for Naive, Astute, and Misled agents. All parameters other than the refinement criterion were held constant. The original-score conditions use n=16 trials per condition. The failure-count conditions use n=16, except for the 4D Astute condition, for which 13 of 16 attempted trials completed. Corrected endpoint and time-resolved comparisons are reported in Appendix C.

To directly test the contribution of the uncertainty term in the stress score, we performed a controlled ablation in which the 3D and 4D simulations were rerun using a scaled failure count $c\;\cdot \;n_{f}$ (based on Equation (20)) as the refinement criterion, with all other parameters held constant (Figure 8). The original-score conditions use n=16 trials throughout, as did the failure-count conditions, except for the 4D Astute condition, for which 13 of 16 attempted trials completed. At the final measurement, the original stress score produced higher R(G) for the 3D Naive (U=202, $r_{\mathrm{rb}}=0.578$, $p_{\mathrm{Holm}}=0.0224$) and 3D Misled agents (U=208, $r_{\mathrm{rb}}=0.625$, $p_{\mathrm{Holm}}=0.0137$), whereas the failure-count criterion produced higher R(G) for the 4D Naive agent (U=41, $r_{\mathrm{rb}}=-0.680$, $p_{\mathrm{Holm}}=0.00668$). No corrected difference was detected in the other three comparisons. Thus, the uncertainty-weighted score can improve refinement relative to failure count alone, but does not do so uniformly across dimensions or prior-knowledge conditions. Because both conditions retain failure-driven refinement, this comparison isolates the uncertainty weighting in the refinement criterion rather than the contribution of refinement itself.

Why these search biases interact with dimensionality in this way is currently unclear. One possible contributor is the affordance threshold mechanism: edge affordance depends on the hyperball field measure, the scale of which is strongly dimension-dependent, so fixed threshold adaptation dynamics may change which edges are used and consequently which failed edges become eligible for refinement as dimensionality increases. However, the present experiment does not isolate this mechanism. More broadly, these results show that the effects of prior knowledge and refinement criteria emerge from their interaction with the complete segraph search dynamics, and that dimensionally stable mechanisms for biasing this search remain an open problem.

### 7.3. MountainCar

We further tested the ARMS algorithm on Continuous MountainCar, a standard reinforcement learning task. Instead of two spatial dimensions, its state space contains one position and one velocity dimension. This makes low-level control less trivial, as it is usually impossible to simply “move towards the goal” because the dimensions are dynamically coupled. We used a PID controller with hand-tuned gains for ARMS. We compared it with Proximal Policy Optimization with Random Network Distillation, equipped with the same low-level controller in order to reduce the extent to which controller quality could determine the comparison. This baseline, technically PPO-RND-PID, is referred to as “PPO+”. Unlike the maze environments, MountainCar resets to an initial condition after 1000 timesteps or when the agent reaches the goal. Because our PPO+ implementation requires this reset, both PPO+ and ARMS experience the same reset conditions. ARMS merely “experiences” the reset as an abrupt but otherwise unmarked change in state; it receives no explicit reset signal, performs no reset-specific operation, and retains its segraph without special changes. From the perspective of ARMS, there are no resets, only unusually fast aberrant transitions.

On ordinary MountainCar, we found that both algorithms converged extremely fast. This is because using a PID controller makes it sufficient to pick a goal to the left, then pick a goal to the right on top of the hill. The presence of the wall means that the exact goal location is irrelevant, so the task becomes fairly trivial.

We made a very simple modification to the ARMS algorithm in order to convert it into an RL algorithm, allowing ARMS to spontaneously switch between an “exploration” mode (the default ARMS algorithm) and “exploitation” mode, where the agent selects as its goal the vertex with the highest time-average reward. The decision between exploration and exploitation is made by calculating a weighted measure of the mean-relative reward deviation across all vertices: if the deviation is high (there are some states with *much* higher reward than average), then the agent is more likely to choose to “exploit” the segraph rather than exploring it. Learning still occurs during exploitation, as ARMS is otherwise unchanged, so experience about paths *to* the reward is still being collected. Basically, we converted ARMS into an RL algorithm by simply adding a reward-weighted *bias* to its exploration goals.

**Figure 9:**
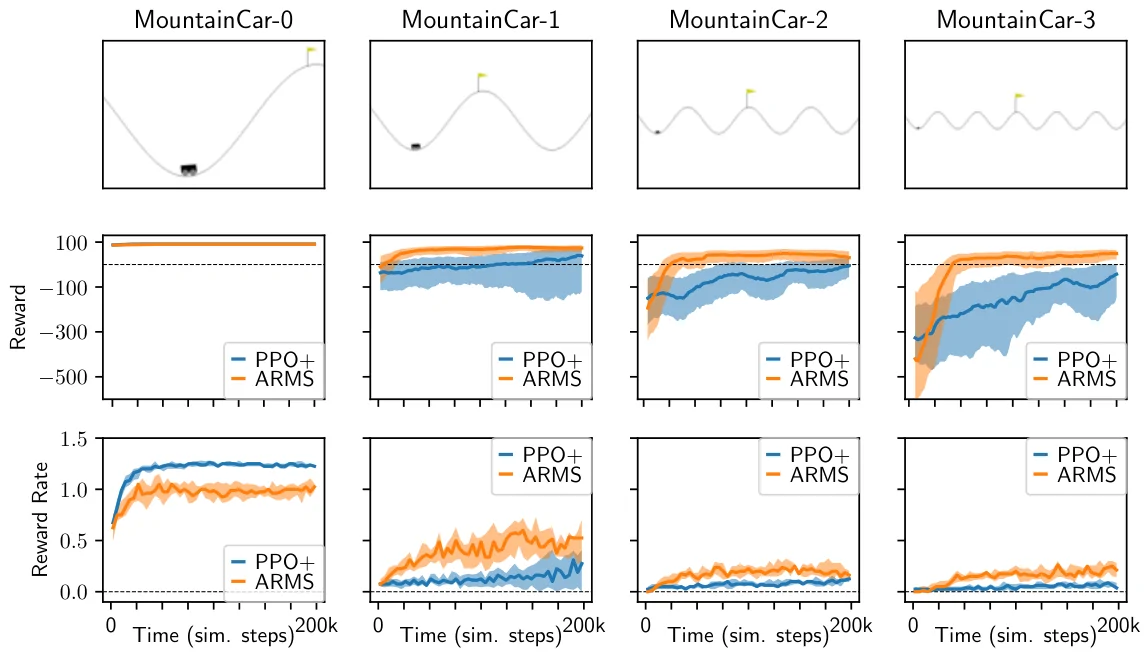
Comparison of ARMS (orange) and PPO+ (blue) on traditional Continuous MountainCar (MountainCar-0) and three increasingly challenging variants with larger state space geometries and stricter goal conditions. The top row provides a direct visual comparison of the environment geometry and goal region, the middle row shows smoothed per-episode reward, and the bottom row shows reward-event rate. Lines and shaded regions show the median and interquartile range over 16 trials. ARMS uses one shared configuration, $c_{\Delta \tilde{q}}=2.0$ and $p_{\mathrm{persist}}=1.0$, selected from the grid evaluated across the four-environment family. PPO+ uses a separately selected configuration for each environment, evaluated in 16 trials not used during its hyperparameter search. Results for all four ARMS grid configurations are shown in Figure A1. Both algorithms experience the same environmental reset following task success or expiration of the episode time limit, while their internal structures (the segraph for ARMS and the learned network weights for PPO+) persist across episode boundaries.

To more strongly test ARMS, we developed much more difficult variants of the MountainCar environment. Instead of the usual +100 reward being granted for merely crossing the goal position, we converted the goal *position threshold* to a goal *radius* in the (position, velocity) state space, so that the reward is only granted to the agent when it is at the top of the hill *and* is also moving very slowly. We also extended the environment past the first hill to make it possible to overshoot the first hill, meaning that the agent cannot simply slam into the wall to achieve the goal. We developed three levels of difficulty (see top row of Figure 9): the first level, MountainCar-1, is as we just described: a valley followed by the hill with the reward, then another valley. The second level, MountainCar-2, has three hills with a reward in the middle (only when moving slowly), while the third level, MountainCar-3, has five hills.

**Figure A1:**
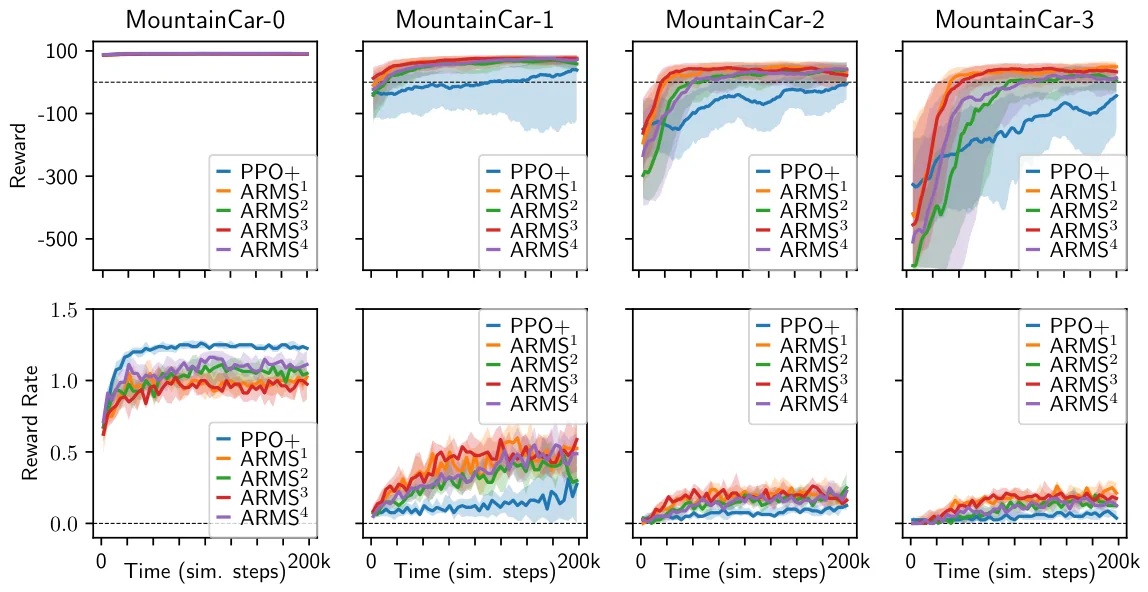
Targeted sensitivity of ARMS to refinement sensitivity $c_{\Delta \tilde{q}}$ and goal persistence $p_{\mathrm{persist}}$ across MountainCar-0 through MountainCar-3. Top panels show the per-episode reward and bottom panels show the reward event rate for the four combinations of $c_{\Delta \tilde{q}}\in \left\{0.455,2.0\right\}$ and $p_{\mathrm{persist}}\in \left\{0.6,1.0\right\}$. Lines and shaded regions show the median and interquartile range over 16 independent 200,000-timestep trials per configuration. ARMS^1^ = ($p_{\mathrm{persist}}=1.0,c_{\Delta \tilde{q}}=2.0$), ARMS^2^ = ($p_{\mathrm{persist}}=1.0,c_{\Delta \tilde{q}}=0.455$), ARMS^3^ = ($p_{\mathrm{persist}}=0.6,c_{\Delta \tilde{q}}=2.0$), and ARMS^4^ = ($p_{\mathrm{persist}}=0.6,c_{\Delta \tilde{q}}=0.455$).

We performed a small 2×2 hyperparameter grid search for ARMS over $c_{\Delta \tilde{q}}\in \left\{0.455,2.0\right\}$ and $p_{\mathrm{persist}}\in \left\{0.6,1.0\right\}$ in each of MountainCar-0 through MountainCar-3, using 16 independent trials per configuration. After qualitatively selecting the overall performance across the four environments, we selected a single shared configuration, $c_{\Delta \tilde{q}}=2.0$ and $p_{\mathrm{persist}}=1.0$. The same 16 trials from the selected ARMS configuration are reported in Figure 9, and the results for all four ARMS configurations are shown in Figure A1. We also performed a much more extensive per-environment hyperparameter search for PPO+ so that a single ARMS configuration could be compared against the best PPO+ configuration in each environment. After finding the best PPO+ configuration in each environment, that configuration was re-run for 16 trials to increase statistical power, as our compute budget restricted us to performing five trials per condition during the PPO+ hyperparameter search (see Appendix B.9). The median total per-episode reward (first and third quartiles shown as shaded region) over 200k timesteps of training is shown for both ARMS (orange) and PPO+ (blue) in the middle row of Figure 9.

To confirm that PPO+ was actually learning the more difficult tasks (since for MountainCar-3 the PPO+ reward never goes above 0, due to the per-time step action penalty outweighing the final reward for goal achievement), we also measured the reward event rate, plotted on the bottom row of Figure 9, which shows that PPO+ is actually gradually improving at the task and reaching the reward. The purpose of these experiments is not to establish that ARMS is generally superior to PPO+ or to other reinforcement learning algorithms but to test whether a concrete implementation of behavioral search can achieve credible performance on continuous control tasks. Thus, PPO+ was given a large environment-specific hyperparameter search in order to reduce the possibility that weak baseline performance could be attributed to an inappropriate configuration. Additionally, the comparison is not a held-out algorithm ranking benchmark, as all four MountainCar environments were consulted when selecting the shared ARMS configuration and its 16 selection trials are also the trials reported in Figure 9. In contrast, the reported PPO+ curves come from independent evaluations performed after environment-specific parameter selection. These differences in the selection and evaluation protocols should be considered when interpreting the comparison.

At the final analyzed point of each trace, all eight environment-by-metric comparisons survived figure-wide Holm correction ($p_{\mathrm{Holm}}\leq 0.0218$, $|r_{\mathrm{rb}}|=0.492$–1.000). PPO+ had higher reward and reward event rate in MountainCar-0, while ARMS had higher values for both outcomes in MountainCar-1 through MountainCar-3. Time-resolved comparisons likewise identified broad figure-wide corrected intervals in every environment and metric (Appendix C). Mean trajectories with sample standard deviations and pointwise bootstrap 95% confidence intervals are also reported in Appendix C.

One possible explanation for the advantage of ARMS in MountainCar-1 through MountainCar-3 is its explicit representation and composition of local transitions. ARMS records each successful or failed local transition directly on the corresponding edge, can reuse reliable edges during subsequent pathfinding, and can inhibit a failed edge across all paths that use it. PPO+ instead has to incorporate the sparse task reward and RND exploration signal through gradual modification of a parameterized policy. The harder MountainCar variants may particularly benefit from retaining and recombining useful local transitions, whereas PPO+ performed better in the simpler MountainCar-0 environment. However, this is only a mechanistic interpretation of the observed results; the experiment does not isolate these mechanisms, and as such does not establish that they caused the performance difference.

All reported evaluation trials were run on a small cluster of NVIDIA Tesla P100 GPUs, while PPO+ hyperparameter search was run on a much larger cluster of regular CPUs. On the P100 hardware, mean wall-clock time per 200,000-timestep maze trial ranged from 30.55–37.03 min across the 2D conditions, 67.25–72.75 min in 3D, and 87.88–139.39 min in 4D. Across the MountainCar environments, mean runtime was 13.94–15.63 min for ARMS and 2.37–3.35 min for PPO+, making ARMS 4.57–6.51 times slower in the present implementation. All four runtime comparisons survived Holm correction ($p_{\mathrm{Holm}}=6.07\times 10^{-6}$, Appendix C). Thus, even where ARMS achieved higher task performance, it did so at substantially greater computational cost in the present implementation.

## 8. Discussion

We have developed a theory of behavioral search in continuous state spaces and constructed ARMS as one finite-memory heuristic realization of that theory. The experiments support the basic feasibility of the integrated algorithm rather than establishing its general superiority: the clearest results were obtained in the 2D mazes and MountainCar variants, while the higher-dimensional mazes exposed dimension-dependent and inconsistent effects of prior knowledge and refinement criterion. Comparison against PPO+ provides a task-relevant baseline but does not establish superiority over reinforcement learning, archive-based, goal-conditioned, novelty search, or planning methods more generally. The following sections delimit the assumptions and scope of the theory, the empirical attribution supported by the present experiments, and the principal representational, scaling, and algorithmic limitations of ARMS.

### 8.1. Assumptions, Boundary Conditions, and Limitations

#### 8.1.1. Boundary Conditions and Assumptions

Our work assumes a directly and fully observable finite-dimensional Euclidean state space with a provided coordinate representation rather than a learned one. Observations, actuators, and state dynamics are also assumed to be noiseless and deterministic. Therefore, the present formulation does not address partial observability, latent state inference, stochastic dynamics, or sensor and actuator noise. We also assume that there are neither absorbing nor Garden-of-Eden regions of the state space. These restrictions isolate behavioral search from representation learning and system identification, but sharply limit the present work’s real-world applicability.

ARMS must also be supplied with a low-level goal-directed controller and termination criteria, and provides no means of improving that controller. A goal is solvable only if there exists a finite composition of transitions that the controller can execute robustly; otherwise, neither the ideal construction nor ARMS can solve it. Moreover, the formal problem is primarily attainment of the goal state. Objectives involving accumulated costs, constraints, or changing reward functions require additional mechanisms. The simple maze and MountainCar experiments consequently provide a proof of concept rather than evidence of broad applicability.

#### 8.1.2. Scope of Behavioral Search Theory

The ideal theory guarantees complete enumeration only under a fair and balanced extension-and-refinement schedule, and guarantees success only in finite but otherwise unbounded time. Finite-memory ARMS does not inherit this result: epistemic selection, stress-guided refinement, and affordance thresholding only heuristically approximate the required schedule. In addition, ARMS deletes vertices after reaching a fixed capacity, and this ad hoc deletion is not containment-preserving. Thus, ARMS may fail to find an existing robust solution when its heuristics or deletion prevent the segraph from representing a necessary region at sufficient precision. Further work is needed to characterize when these departures can be controlled and to develop theoretically justified deletion criteria, potentially also clarifying the functional role of forgetting in biological systems.

#### 8.1.3. Component Attribution and Ablation

The present experiments do not provide a full factorial ablation of ARMS. Removing extension or refinement would prevent expansion of coverage or local resolution, respectively, producing useful failure controls but no longer approximating the basic theoretical construction. Epistemic goal selection, affordance threshold adaptation, reward-biased goal selection, and vertex deletion could instead be replaced or removed while retaining a recognizable behavioral search algorithm, but were not independently ablated. Therefore, Table 2 separates each component’s intended role from the empirical evidence actually provided for it.

Consequently, the experiments support only limited component-level attribution. They show mixed effects of the uncertainty term relative to failure count, consequential but dimensionally inconsistent effects of the supplied extension bias, and continued function across the tested initial field scale and MountainCar parameter settings. They do not establish that every component is necessary or quantify all independent contributions and interactions, as doing so would require a factorial comparison of alternative goal selection, thresholding, extension, refinement, deletion, and reward bias mechanisms.

#### 8.1.4. Empirical Failure Cases and Evaluation Limitations

The experiments contain several cases in which ARMS underperforms or behaves inconsistently. First, the supplied extension bias does not produce a consistent ordering of Naive, Astute, and Misled agents. In the maze-swap experiment, the Astute agent initially converges quickly but recovers more slowly following the environmental change. In the higher-dimensional experiments, the Naive agent has the highest final median reliability in 3D but largely collapses in 4D, where its final median R(G) is 0.144. The Astute agent instead performs relatively well in 4D, showing that neither dimensionality nor the supplied information has a simple independent relationship with performance.

Second, the controlled refinement criterion ablation does not identify a uniformly superior criterion. The uncertainty-weighted stress score outperforms scaled failure count for the 3D Naive and Misled agents, but the scaled failure-count criterion outperforms it for the 4D Naive agent. This reversal shows that refinement behavior depends on its interaction with the dimensionality, extension bias, affordance threshold, and developing segraph rather than only on the local information contained in the refinement score.

Third, ARMS does not uniformly outperform PPO+. PPO+ achieves significantly higher reward and reward event rate in the standard MountainCar-0 environment, while ARMS performs better on the three constructed variants. ARMS is also computationally more expensive in the present implementation, requiring 4.57–6.51 times the wall-clock runtime of PPO+ across the four MountainCar environments. Thus, the results show a possible advantage for ARMS in the more difficult sparse reward variants but also a disadvantage in the standard environment together with a substantial computational cost disadvantage.

Finally, the reported metrics do not evaluate path optimality, memory consumption, energy efficiency, noisy or partially observable conditions, transfer between environments, or performance with a learned low-level controller. The present evaluation demonstrates selected capabilities and exposes several important failures, but does not establish general robustness or superiority.

#### 8.1.5. Representational and Scaling Limitations

One of the major limitations of our formulation of behavioral search and the ARMS algorithm itself is the use of radially symmetric hyperball vertex fields. Hyperballs provide a simple and easy-to-use representation of neighborhoods in a Euclidean space, and arise naturally in our theoretical construction of robust behavior, but arguably offer a suboptimal decomposition of state space, especially in higher dimensions. Place fields in the hippocampus are also known to depend on environmental geometry and do not need to be radially symmetric, including elongated fields found in narrow corridors. More general field geometries such as ellipsoidal fields aligned with the locally traversable directions of the environment could potentially represent such environments more efficiently, but are not considered here.

There is also a direct dimensional dependence in the quantities used by ARMS. For a *d*-dimensional hyperball vertex field of radius σ,

$$M\left(v\right)=\frac{\pi^{d/2}}{\Gamma (d/2+1)}\sigma^{d},$$

while the measure of an edge field is $M\left(e_{i,j}\right)=M\left(v_{i}\right)M\left(v_{j}\right)$. Therefore, scaling the radius of one endpoint by a factor *a* scales the corresponding edge measure by $a^{d}$, while scaling both endpoints scales it by $a^{2d}$. For the factor $\sqrt{2}$ used during refinement and extension, these factors become $2^{d/2}$ and $2^{d}$, respectively. Because the edge field measure contributes directly to estimated affordance and epistemic score, fixed affordance threshold and goal selection dynamics need not have the same effective behavior as dimensionality changes. This provides one plausible explanation for the dimensional dependence observed in the experiments; however, the present experiments do not isolate this mechanism.

We did not perform a controlled measurement of vertex and edge counts as a function of dimension or a higher-dimensional sensitivity analysis of the affordance threshold adaptation parameters. Therefore, the present results do not establish an empirical scaling law for ARMS, and our practical claims are restricted to the low-dimensional environments in which performance was reliable. Our current mechanism for incorporating prior knowledge is likewise preliminary and ad hoc. The varying effects observed across dimension and refinement criterion show that such search biases can be consequential, but we do not yet have a general method for biasing behavioral search in a dimensionally stable way.

#### 8.1.6. Limitations of Planning and Learning

The present pathfinding algorithm represents binary reachability rather than transition costs such as energy, time, or reward. Cost minimization would require a different pathfinding procedure. Planning also occurs over a flat graph without clustering, abstraction, or hierarchy, and ARMS cannot consolidate its graph into procedural or semantic knowledge that is transferable to future problems. Finally, more complex environments may require a low-level controller that ARMS cannot presently learn or improve. The experiments should be understood as a proof of concept in simple continuous environments rather than evidence of general planning, learning, or real-world capability.

### 8.2. Related Work

This work intersects reinforcement learning, exploration, and planning. Table 3 summarizes its relationship to the closest varieties of methods. The purpose is not to argue that these alternatives are infeasible or that ARMS is generally superior but to locate the particular theoretical and architectural contribution made in this paper.

Goal-conditioned reinforcement learning organizes behavior around explicit goals. Universal value function approximators condition value estimates on state and goal [3], while hindsight experience replay relabels unsuccessful episodes with achieved goals to improve sparse reward learning [4]. These methods learn reusable goal-conditioned policies or values, and may generalize much more efficiently than ARMS. ARMS instead retains an explicit representation of local transitions and composable plans, and does not intrinsically require external reward. These tradeoffs were not empirically compared.

Go-Explore is strategically close to ARMS because it remembers reached regions and uses them to organize further exploration [5]. ARMS stores local transition information in a continuously navigated segraph, whereas Go-Explore uses an archive of representative cells and trajectories and may return through simulator restoration or a goal-conditioned policy. This makes Go-Explore a particularly relevant missing empirical comparator.

IMGEPs generate and order goals using intrinsic signals, producing an automatic curriculum and repertoire of skills [6]. This resembles epistemic goal edge selection, but IMGEPs ordinarily learn goal-parameterized policies rather than constructing an enumerable plan representation. Novelty search and quality diversity methods instead maintain diverse behavior-producing solutions, often in an archive [7,8]; instead, ARMS represents potential behaviors as paths through one mutable graph.

RRT and PRM also construct graph-like structures in continuous state spaces and perform well in high-dimensional planning [15,16]. However, they ordinarily assume a geometric free space in which transition feasibility can be queried, whereas ARMS estimates transition reliability through physical attempts. This locality makes inefficient exploration costly and suggests that better control of attempted edges could substantially improve ARMS. In addition, these methods are probabilistically complete, while ARMS heuristically approximates resolution-completeness.

MuZero combines learned dynamics with Monte Carlo tree search, and has also been extended to continuous domains [17,18]. Its forward model simulates futures in a search tree rebuilt at each timestep. ARMS instead maintains and incrementally modifies a persistent graph of possible state–state transitions while delegating actions to a low-level controller.

Finally, the epistemic score in Equation (21) resembles count- or visitation-based curiosity [19]: large underexplored edges receive higher priority, and visiting them reduces that priority. Unlike novelty search archives, this signal operates over regions and transitions within one graph.

The novelty claimed here is the combination of a single mutable graph-based index of continuous plans with extension and refinement, together with the ideal construction showing how such an index can generate a countable dense family of plans. The particular mechanisms used by ARMS to approximately realize this construction are heuristic and overlap with mechanisms found in prior methods, and the present experiments do not establish that these mechanisms are preferable to the alternatives summarized above.

### 8.3. Conclusions

We introduce hyperadaptability as an ideal theoretical limit of behavioral adaptability: the ability of an agent, within the stated assumptions, to solve any solvable continuous problem in finite time regardless of its prior knowledge or experience. The central theoretical contribution is a graph-based construction that uses balanced extension and refinement to incrementally generate a countably infinite dense family of plans. This makes systematic behavioral search possible while allowing experience to alter the order in which plans are tried. ARMS provides one finite-memory heuristic realization of this construction, but does not inherit its theoretical guarantee.

Empirically, ARMS reached a final median R(G)=1.000 in every prior-knowledge condition across the four fixed 2D mazes after 200,000 timesteps and remained stable across the tested 25-fold variation in initial field scale. The higher-dimensional results were substantially less consistent: the ordering of Naive, Astute, and Misled agents changed between 3D and 4D, and the controlled refinement criterion ablation favored the original uncertainty-weighted stress score in the 3D Naive and Misled conditions but favored failure count in the 4D Naive condition, with no corrected difference in the remaining comparisons. On MountainCar, all eight final environment-by-metric comparisons between ARMS and PPO+ survived correction: PPO+ performed better on MountainCar-0, while ARMS performed better on MountainCar-1 through MountainCar-3. However, ARMS also required 4.57–6.51 times the wall-clock runtime of PPO+ in the present implementation.

Taken together, these results provide preliminary evidence that an integrated finite-memory behavioral search algorithm can function in low-dimensional continuous environments, though also exposing substantial limitations in computational cost, dimensional scaling, component attribution, and integration of prior knowledge. The main contribution of this paper is the theoretical construction and its concrete proof-of-concept realization.

## Figures and Tables

**Table 1:** R(G) values after 200,000 timesteps of simulation, reported for the Naive, Astute, and Misled agents in each maze as (Q1, **median**, Q3). The 2D maze, maze-swap, and sensitivity conditions use n=8 completed trials per condition, while all 3D and 4D conditions use n=16 completed trials per condition. Corresponding endpoint, time-resolved, mean/SD, and confidence interval analyses can be found in Appendix C.

Maze	Naive Agent	Astute Agent	Misled Agent
5 × 5(1)	(1.000, **1.000**, 1.000)	(1.000, **1.000**, 1.000)	(1.000, **1.000**, 1.000)
5 × 5(2)	(0.999, **1.000**, 1.000)	(1.000, **1.000**, 1.000)	(1.000, **1.000**, 1.000)
Triangle	(1.000, **1.000**, 1.000)	(1.000, **1.000**, 1.000)	(1.000, **1.000**, 1.000)
Pentagon	(1.000, **1.000**, 1.000)	(1.000, **1.000**, 1.000)	(1.000, **1.000**, 1.000)
Maze Swap	(1.000, **1.000**, 1.000)	(0.888, **0.955**, 1.000)	(0.998, **0.999**, 1.000)
3D Mazes	(0.924, **0.978**, 0.992)	(0.782, **0.821**, 0.914)	(0.611, **0.909**, 0.944)
4D Mazes	(0.106, **0.144**, 0.292)	(0.603, **0.757**, 0.813)	(0.223, **0.413**, 0.661)

**Table 2:** Functional and empirical attribution of the main ARMS components. “Not ablated” means that the present experiments did not estimate that component’s independent contribution to performance.

Component	Intended Function	Empirical Status
Segraph representation	Stores state space regions, local transition experience, and composable paths.	Used in every ARMS condition and not ablated. The experiments evaluate the integrated segraph-based algorithm rather than this representation in isolation.
Extension	Expands segraph coverage by adding candidate vertices around successfully reached regions.	Used in every condition and not ablated. Naive, Astute, and Misled conditions manipulate the information used to bias candidate admission, rather than ablating extension itself.
Refinement	Increases local resolution around failed transitions.	Used in every condition and not ablated. The controlled refinement-criterion comparison changes when refinement is triggered, rather than testing refinement against no refinement.
Uncertainty term in the stress score	Concentrates refinement on edges whose estimated reliability remains uncertain.	Directly ablated by replacing the original stress score with a scaled failure count while holding all other parameters constant. The effect was significant in three of six higher-dimensional comparisons and differed in direction across conditions.
Epistemic goal selection and affordance threshold	Distribute exploration across under-sampled edges and prioritize paths according to estimated reliability and field measure.	Used in every condition and not independently ablated. Their individual performance contributions therefore remain unknown.
Prior-guided extension bias	Uses supplied geometric information to alter which extension candidates are admitted.	Manipulated through the Naive, Astute, and Misled conditions. Its effects were inconsistent across dimensions, so the experiments do not establish a general benefit.
Goal persistence	Controls whether ARMS continues pursuing the same goal after a failed transition.	Varied together with refinement sensitivity in the 2 × 2 MountainCar sensitivity analysis. This tests a limited parameter interaction rather than removal of the mechanism.
Reward-biased goal selection	Allows ARMS to alternate between exploratory goals and goals associated with high observed reward.	Used in the MountainCar experiments and not independently ablated. Its contribution cannot be separated from that of the underlying behavioral search.
Vertex deletion	Restricts the segraph to a finite representational capacity.	Used when capacity was reached and not independently ablated. It is a practical finite-memory heuristic rather than a theoretically required component.

**Table 3:** Comparison of ARMS with closely related method families and the empirical PPO+ baseline. The distinctions describe the usual formulation of each family rather than properties that must hold for every possible variant.

Method	Memory Representation	Search/Learning Signal	Reward and Reset Procedure	Primary Difference from ARMS
ARMS	Mutable segraph with local transition failure/success counts	Epistemic score, estimated reliability, affordance threshold, vertex-visitation	External reward and reset not intrinsically required, but are compatible	
Goal-conditioned RL/HER [3,4]	Goal-conditioned policy or value function, generally with replayed experience	Goal-specific reward together with generalization or hindsight relabeling	Uses an explicit goal-success or reward definition. HER reuses episodic experience	Learns a reusable mapping from states and goals to actions or values rather than an enumerable representation of plans
Go-Explore [5]	Archive of representative cells and trajectories, optionally with a goal-conditioned return policy	Selection of promising archived cells followed by return and further exploration	Designed for sparse or deceptive rewards, returning may use simulator restoration or a learned policy, depending on the variant	Stores reached states or cells in an archive rather than local transition fields in a continuously navigated graph
IMGEP [6]	Repertoire of generated goals and goal-parameterized policies or models	Intrinsic goal selection, competence, novelty, or learning progress	Does not require one externally supplied target goal, trial and reset structure depends on the implementation	Constructs an autotelic goal curriculum and skill repertoire rather than an explicitly enumerable plan-space
Novelty search and quality diversity [7,8]	Population or archive of behavior-producing solutions	Behavioral novelty and, for quality diversity, local solution quality	External task quality may be absent or combined with novelty, candidate behaviors are ordinarily evaluated separately	Maintains diverse candidate solutions rather than paths through one online transition graph
RRT and PRM [15,16]	Search tree or roadmap over configurations	Random sampling and geometric or collision feasibility	No task reward is needed, but the configuration space and transition-feasibility queries are assumed to be available	Plans using queryable geometric information rather than estimating local transition reliability solely through physical attempts
PPO+	Parameterized policy and value networks	External task reward together with RND intrinsic reward	Uses task reward and environmental episode resets in the present experiment	The only direct empirical baseline in this paper, it is a task-learning comparison rather than the closest architectural relative

**Figure A2:**
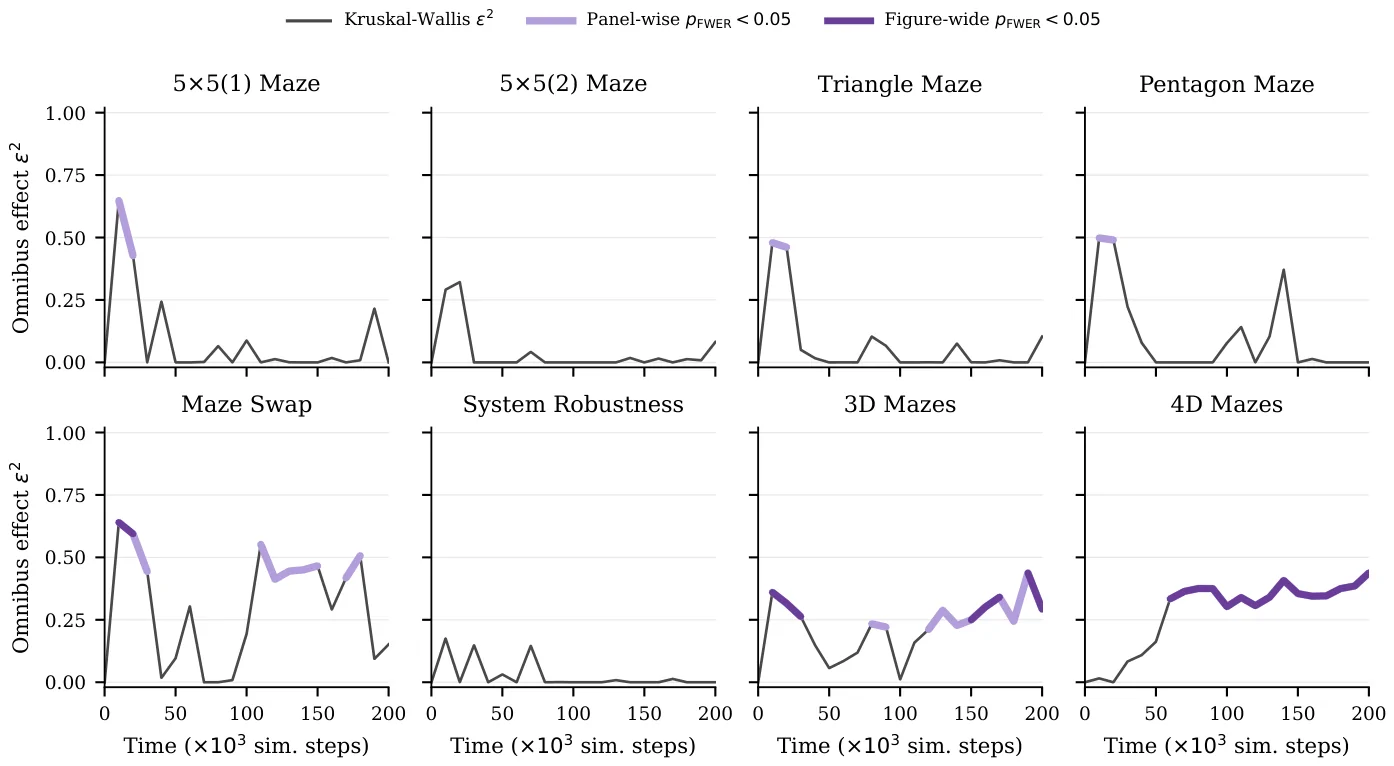
Time-resolved Kruskal–Wallis effect sizes for Figure 7. Light and dark segments indicate $p_{\mathrm{FWER}}<0.05$ after panel-wide and figure-wide whole-trajectory permutation correction, respectively. The imposed R(G)=0 value at t=0 is not tested. The first six panels use n=8 per condition, 3D uses n=16, and 4D uses n=16.

**Figure A3:**
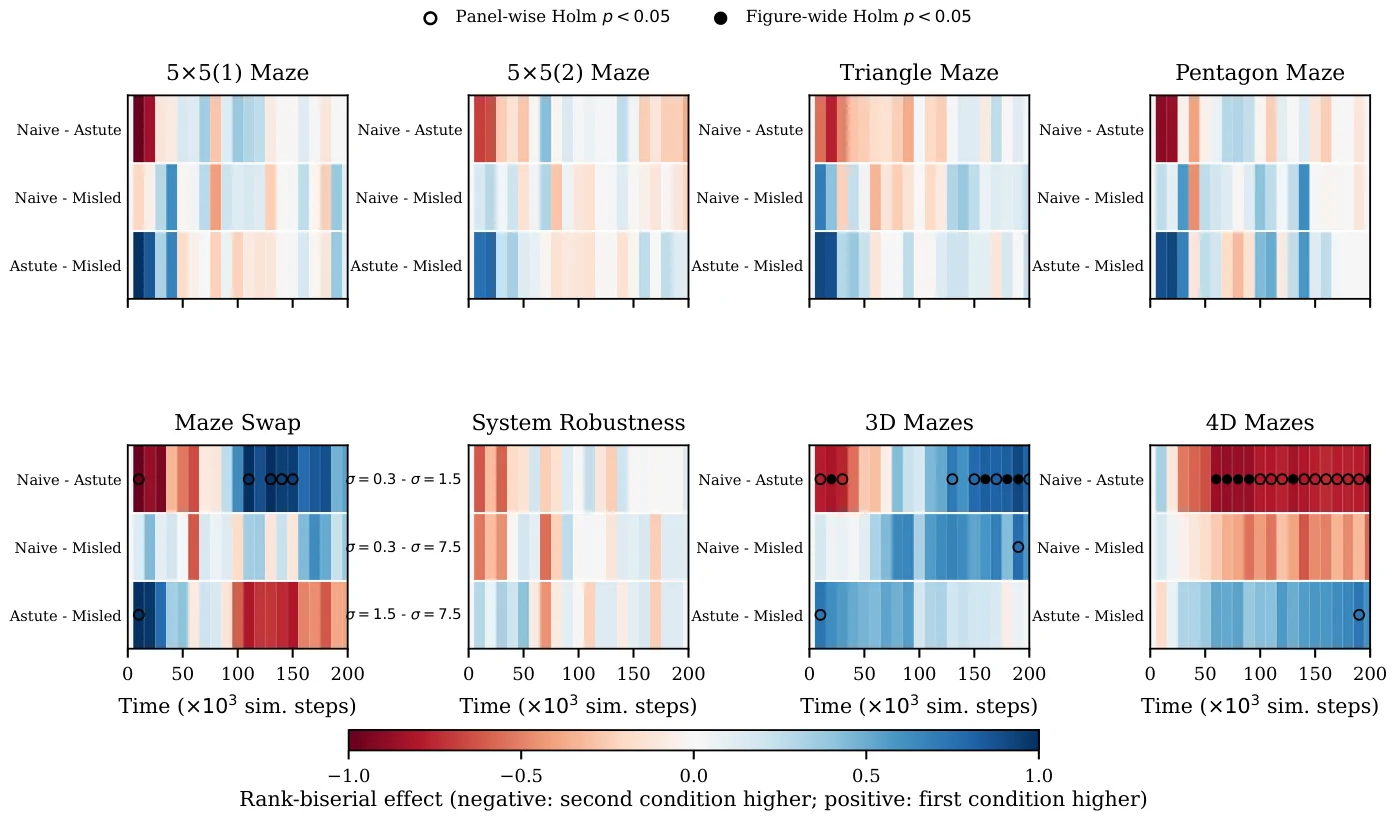
Time-resolved pairwise rank-biserial effects for Figure 7. Positive values indicate that the first listed condition had higher R(G), while negative values indicate that the second condition was higher. Open and filled circles indicate significance after Holm correction within the panel across pairs and time and across the complete figure, respectively. Sample sizes are as reported in Figure A2.

**Figure A4:**
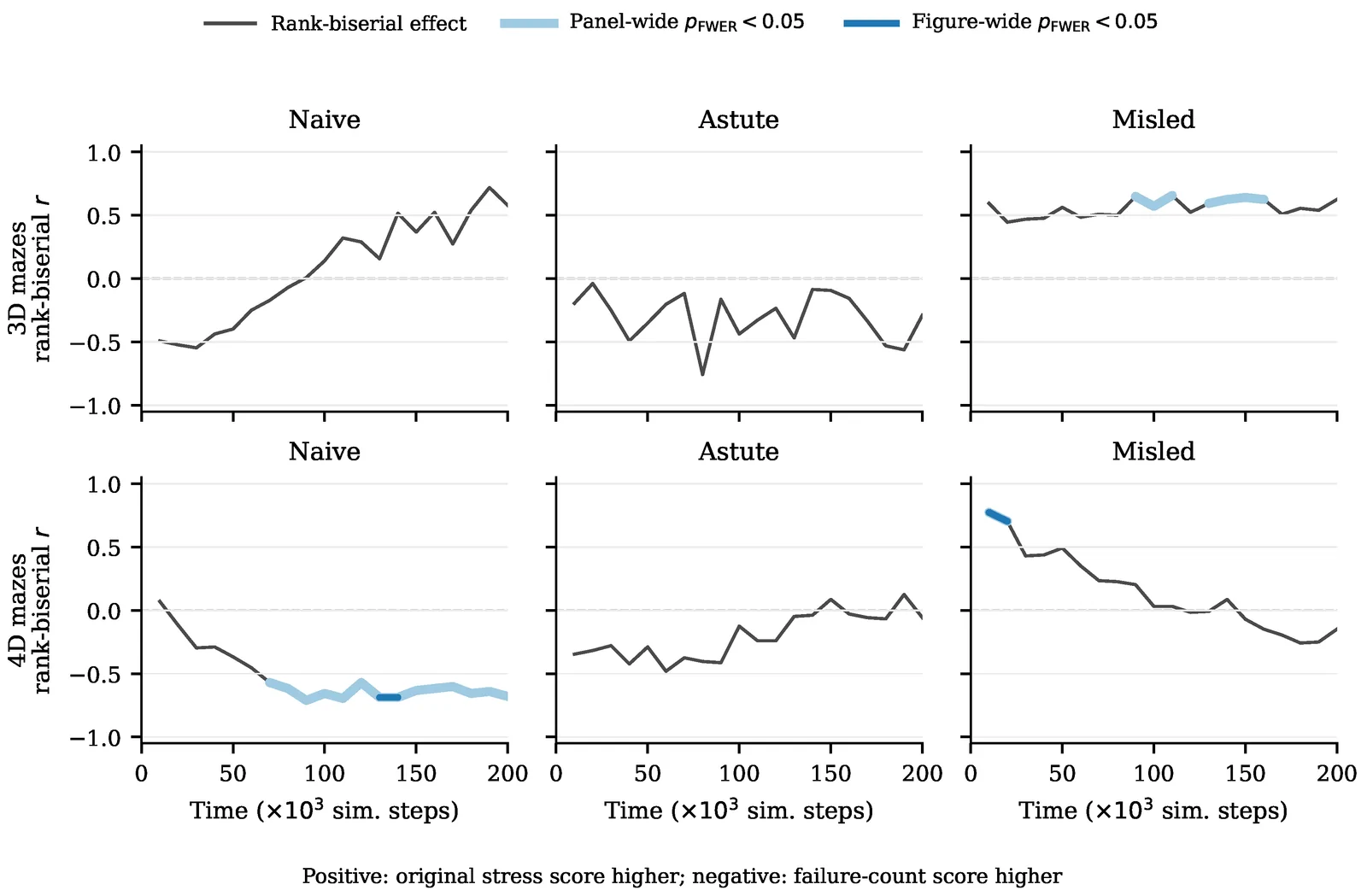
Time-resolved rank-biserial effects comparing the original stress score with the scaled failure-count refinement criterion. Positive effects indicate higher R(G) under the original score, while negative effects indicate higher R(G) under the failure-count criterion. Light and dark segments indicate panel-wide and figure-wide whole-trajectory permutation-corrected $p_{\mathrm{FWER}}<0.05$, respectively. Original-score conditions use n=16 throughout, as do the failure-count conditions, except for 4D Astute, which uses n=13. The imposed R(G)=0 value at t=0 is not tested.

**Figure A5:**
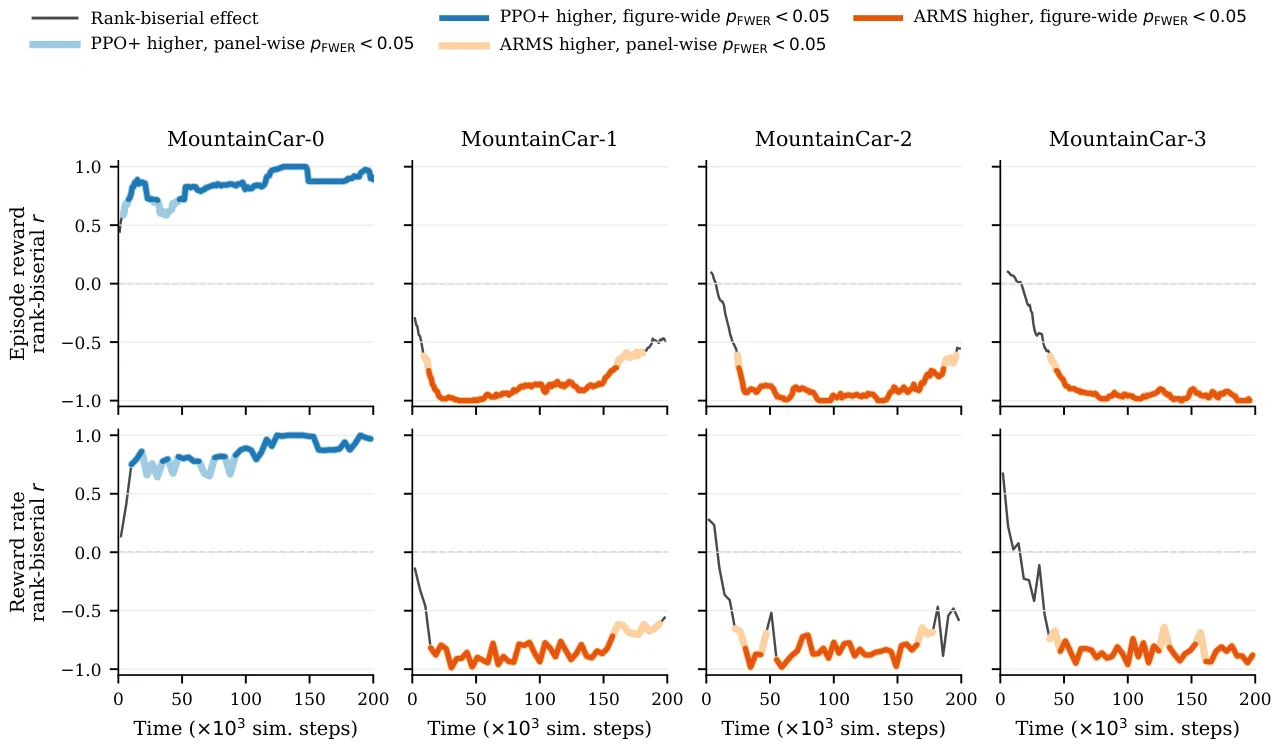
Time-resolved rank-biserial effects comparing PPO+ with ARMS in Figure 9. Positive effects indicate higher PPO+ values and negative effects indicate higher ARMS values. Light and dark segments indicate panel-wide and figure-wide permutation-corrected $p_{\mathrm{FWER}}<0.05$, respectively. Each comparison uses n=16 trials per algorithm.

**Figure A6:**
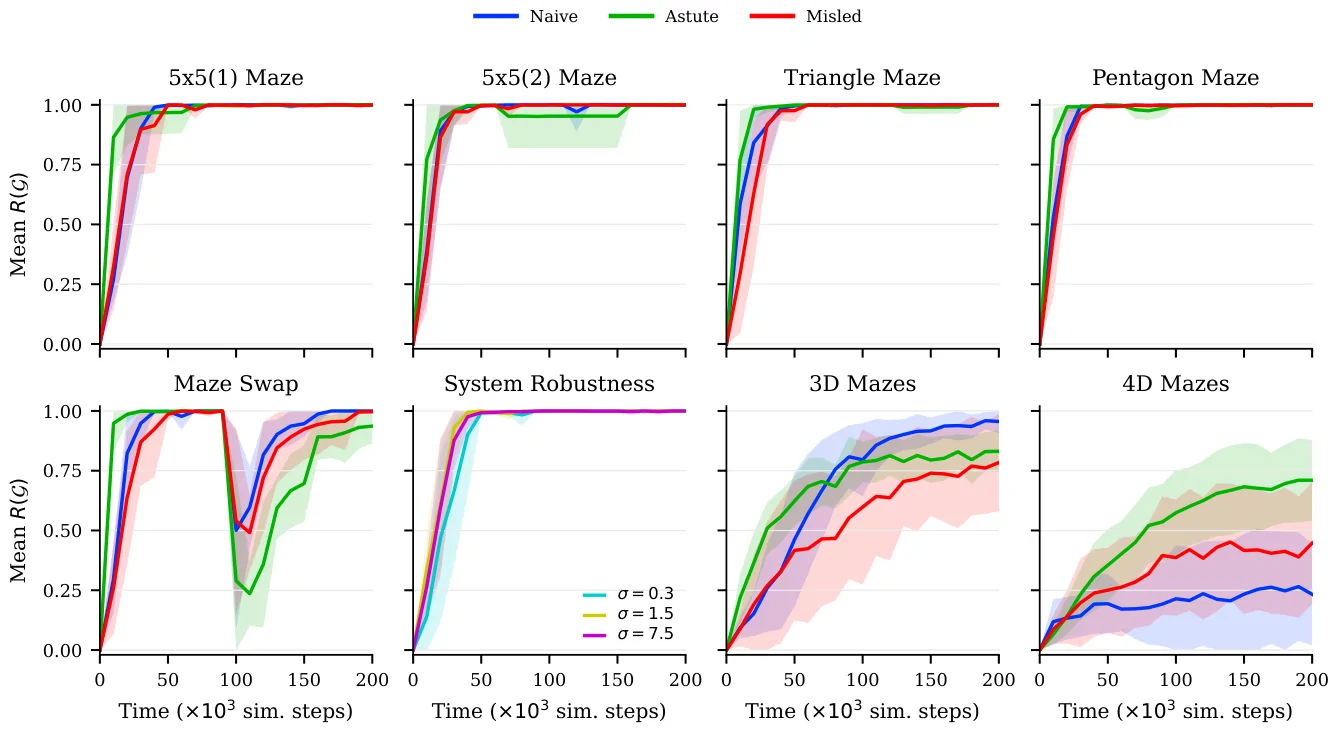
Mean R(G) trajectories corresponding to Figure 7. Shading shows the sample standard deviation. The imposed initial value is R(G)=0. Sample sizes are n=8 per condition in the first six panels, n=16 in 3D and 4D.

**Figure A7:**
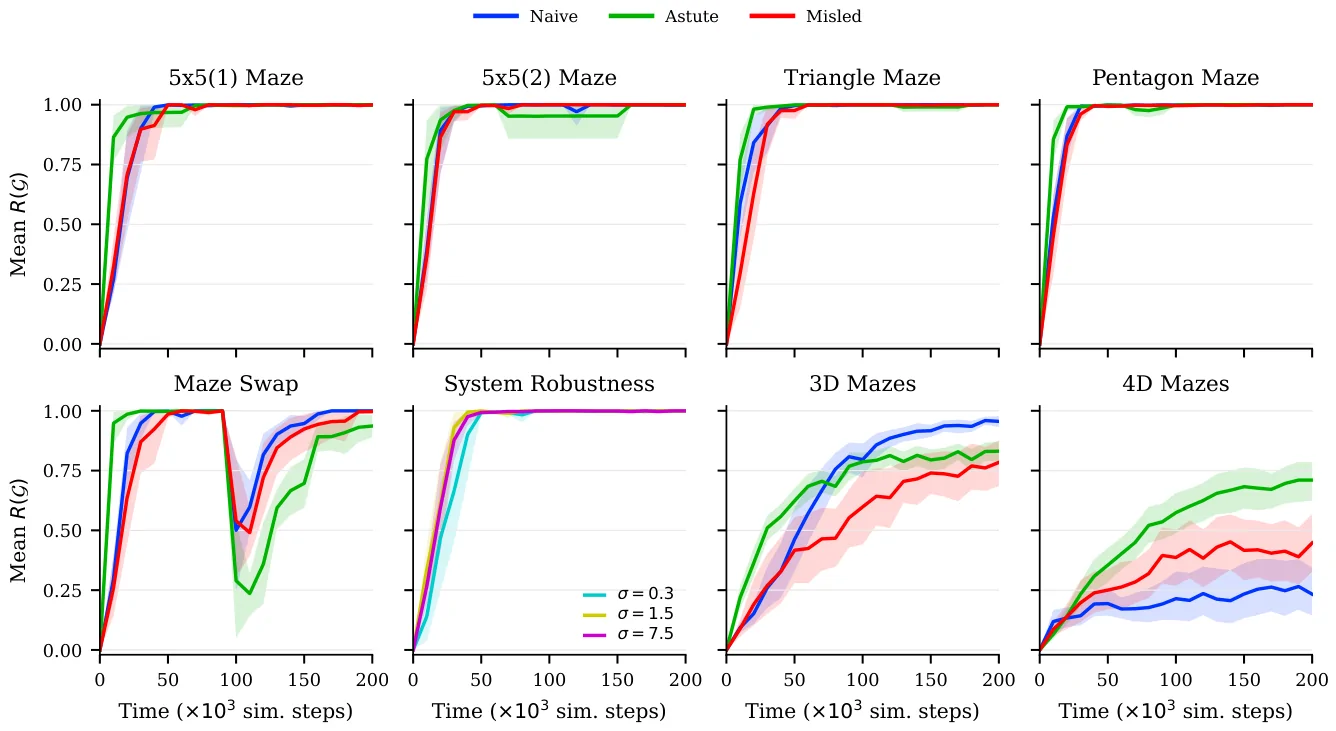
Mean R(G) trajectories corresponding to Figure 7. Shading shows pointwise percentile-bootstrap 95% confidence intervals for the mean based on 10,000 resamples. Sample sizes and the imposed initial value are as in Figure A6.

**Figure A8:**
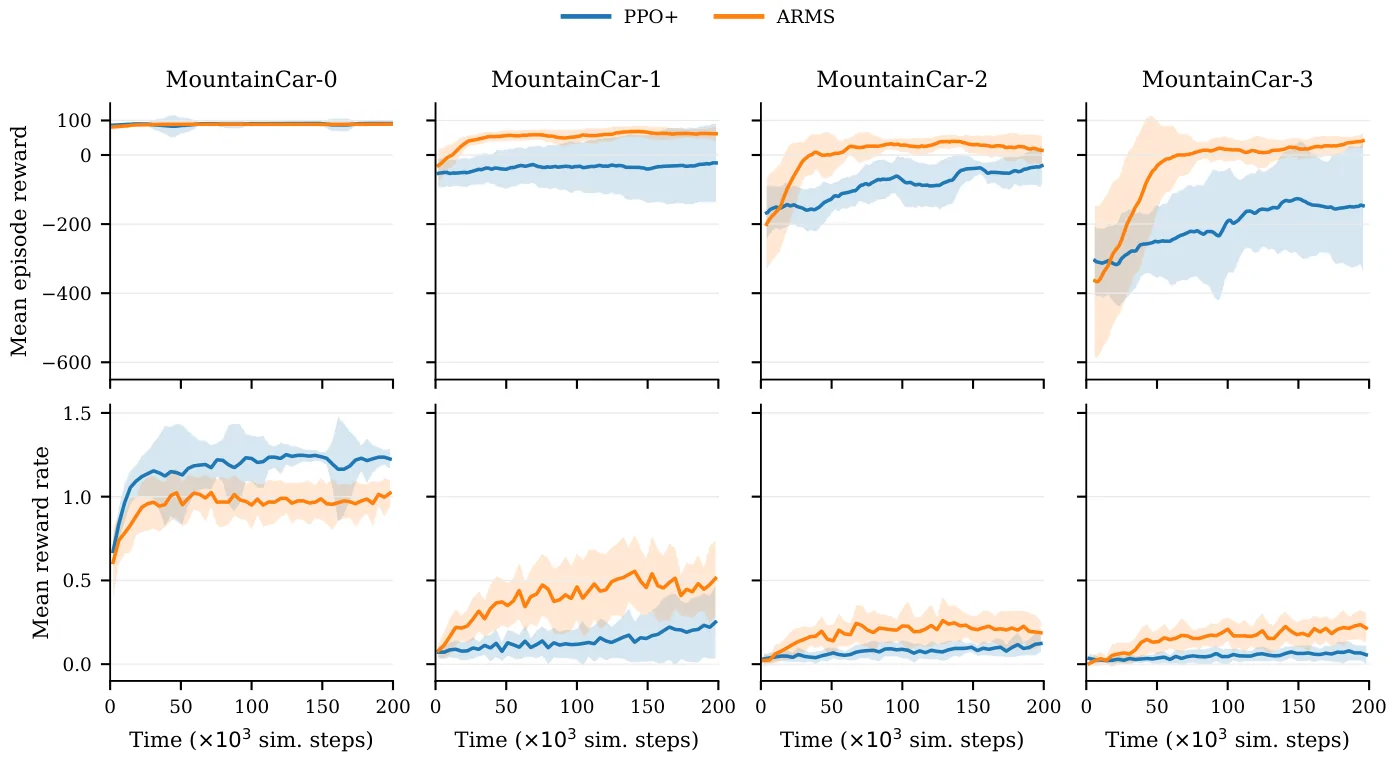
Mean smoothed per-episode reward and mean reward-event rate corresponding to Figure 9. Shading shows the sample standard deviation across n=16 trials per algorithm and environment.

**Figure A9:**
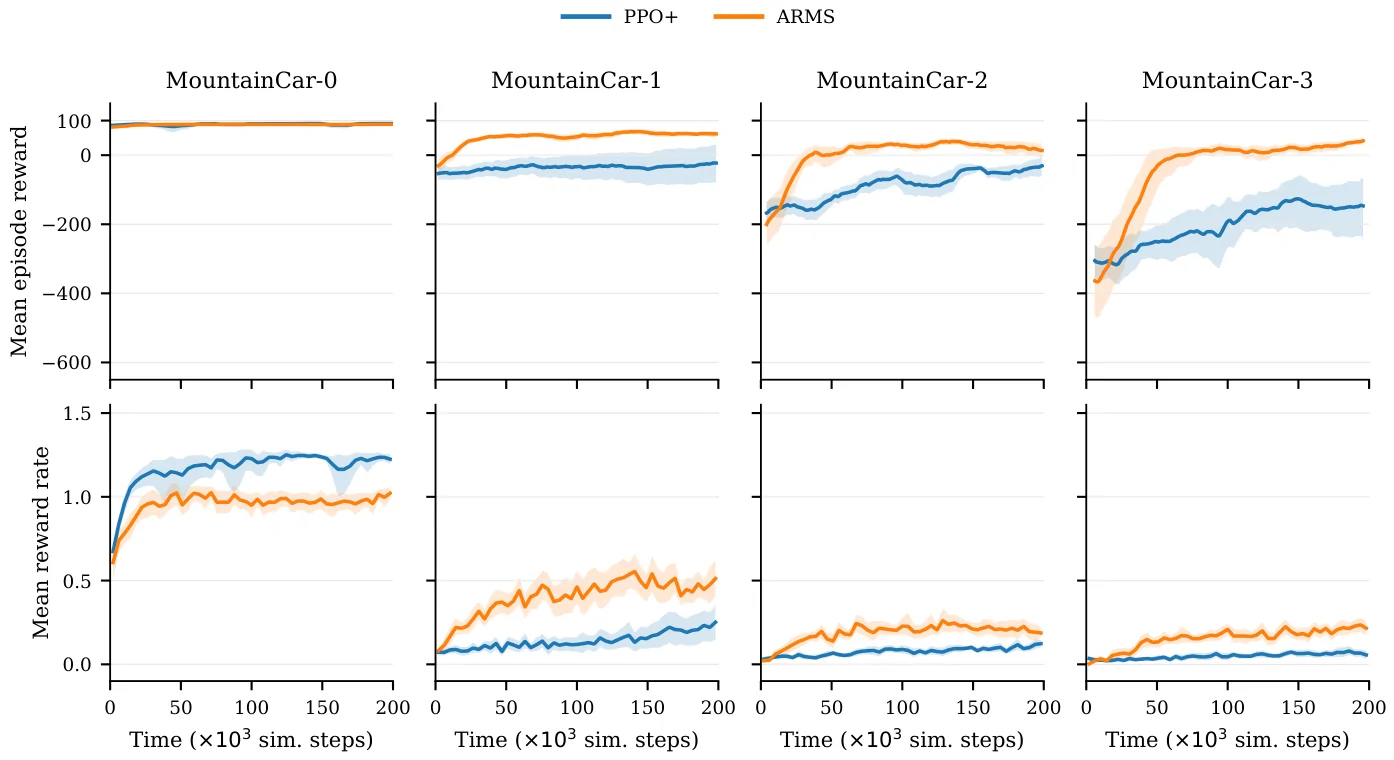
Mean smoothed per-episode reward and mean reward-event rate corresponding to Figure 9. Shading shows pointwise percentile-bootstrap 95% confidence intervals for the mean based on 10,000 resamples and n=16 trials per algorithm and environment.

**Figure A10:**
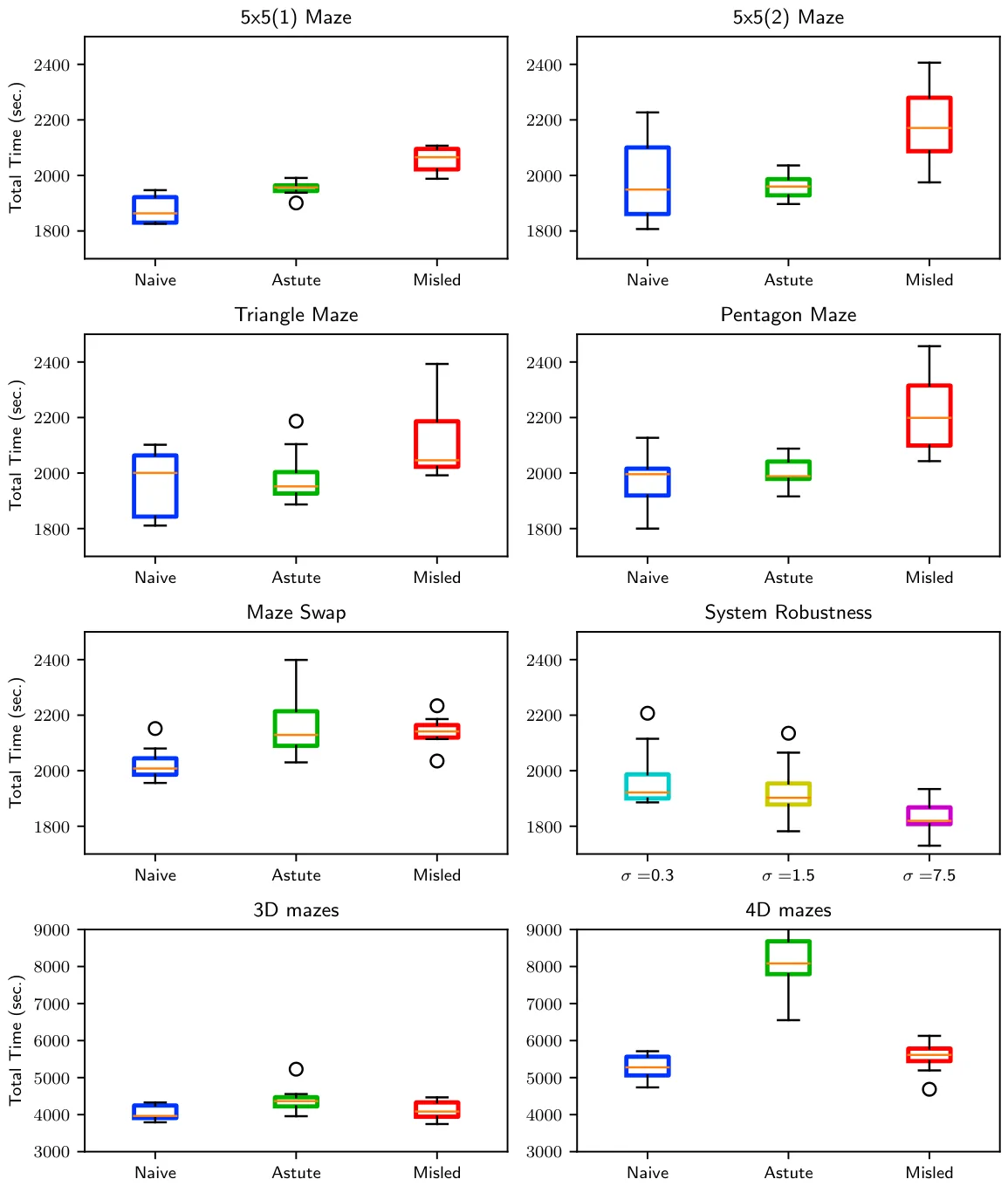
Total wall-clock runtime of completed 200,000-timestep trials corresponding to Figure 7. Boxes show medians and interquartile ranges. The first six panels use n=8 per condition, 3D and 4D use n=16.

**Figure A11:**
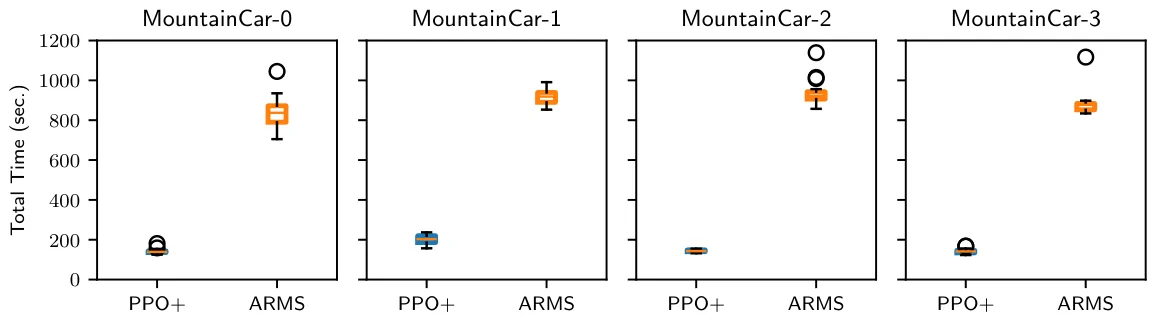
Total wall-clock runtime of completed 200,000-timestep MountainCar trials on NVIDIA Tesla P100 hardware. Boxes show medians and interquartile ranges for n=16 trials per algorithm and environment.

## Data Availability

The complete raw simulation archive is available at https://huggingface.co/datasets/Hazmatius/HyperadaptabilityByBehavioralSearch (accessed on 4 September 2026). Source code, experiment configurations, analysis scripts, and the compact processed performance measurement data underlying the figures are available at https://github.com/oist-cnru/hyperadaptability (accessed on 4 September 2026).

## Appendix A. Rational Behaviors

### Appendix A.1. General Mathematical Machinery

Let $O_{S}=\left\{B_{\epsilon }\left(s\right):s\in S,\epsilon \in R^{+}\right\}$ be the set of all hyperballs in *S*, and let $P_{\mathrm{fin}}^{+}\left(O_{S}\right)$ be the set of all nonempty finite subsets of $O_{S}$. We fix an arbitrary nonempty finite initial “seed” set $B_{0}\in P_{\mathrm{fin}}^{+}\left(O_{S}\right)$, which will be repeatedly transformed. Throughout, B denotes a generic nonempty finite set of hyperballs.

**Definition** **A1** (Selector Function)**.** *A* selector function* $s:P_{\mathrm{fin}}^{+}\left(O_{S}\right)\to O_{S}$ selects a single hyperball from a set of hyperballs, so that $\forall B\in P_{\mathrm{fin}}^{+}\left(O_{S}\right),s\left(B\right)\in B$. In other words, s:B↦b,b∈B.*

**Definition** **A2** (Generator Function)**.** *A* generator function* $g:O_{S}\to P_{\mathrm{fin}}^{+}\left(O_{S}\right)$ maps a hyperball to a finite set of hyperballs. If $g:b\mapsto B^{\prime }$, we call $B^{\prime }$ the* children *by g of b, or the g-1-descendants of b.*

**Definition** **A3** (Mutator Function)**.** *$m_{s,g}:P_{\mathrm{fin}}^{+}\left(O_{S}\right)\to P_{\mathrm{fin}}^{+}\left(O_{S}\right)$ is a* mutator function*, with *

$$m_{s,g}\left(B\right)=B\cup g\left(s\left(B\right)\right).$$

*When it is clear from context, we will write $m_{j}$ for $m_{s_{j},g_{j}}$.*

From this definition, it is plain to see that the operand of a mutator function is contained in its resultant, giving us the following proposition.

**Proposition** **A1** (Mutation Containment)**.**
*
$B\subset m_{s,g}\left(B\right)$
*

If we had a single mutator function *m*, we could recursively call it on our initial set of hyperballs as $m^{i}\left(B_{0}\right)$ to model mutating the initial set of hyperballs $B_{0}$ with the same mutator function *i* times. If we had multiple different mutator functions, we could choose to apply a different mutator at each step.

**Definition** **A4** (Mutation Schedule)**.** *Let $M=\{m_{1},m_{2},\ldots ,m_{n}\}$ be a finite set of mutator functions, with $m_{j}=m_{s_{j},g_{j}}$. Then, $C:N_{1}\to M$ is a* mutation schedule.

*The* individual schedule* for each mutation function $m_{j}\in M$ is defined such that:*
  (A1) $$k_{m_{j}}\left(i\right)=\min(\{n\in N:|\left\{\ell \in \left[1.\;.n\right]:C\left(\ell \right)=m_{j}\right\}|=i\}).$$
  *In other words, if we increment up C(1),C(2),C(3)…, after $k_{m_{j}}\left(i\right)$ “steps”, $m_{j}$ will have been selected i-times.*
*For brevity, we write $k_{j}$ for $k_{m_{j}}$.*

**Definition** **A5** (Polymutator Function)**.** *The* polymutator function

$${\overset{⋄}{m}}_{M,C}^{i}\left(B_{0}\right)=C\left(i\right)\left({\overset{⋄}{m}}_{M,C}^{i-1}\left(B_{0}\right)\right)$$

*with ${\overset{⋄}{m}}_{M,C}^{0}\left(B_{0}\right)=B_{0}$ describes the sequential application of mutator functions to an initial set $B_{0}$ of hyperballs.*
*For brevity we write ${\overset{⋄}{m}}^{i}$ for ${\overset{⋄}{m}}_{M,C}^{i}$, and when appropriate, we let $B_{i}={\overset{⋄}{m}}^{i}\left(B_{0}\right)$.*

**Corollary** **A1** (Polymutator Order)**.**
*
$n_{1}<n_{2}\Rightarrow {\overset{⋄}{m}}^{n_{1}}\left(B_{0}\right)\subset {\overset{⋄}{m}}^{n_{2}}\left(B_{0}\right)$
*

**Definition** **A6** (Fair Polymutator)**.** *A polymutator function $\overset{⋄}{m}={\overset{⋄}{m}}_{M,C}$ is *fair*if $\forall m_{j}\in M$, $k_{j}:N_{1}\to N_{1}$ and $|k_{j}\left(N_{1}\right)|={ℵ}_{0}$, which is to say that every mutator will be called infinitely many times, eventually.*

**Proposition** **A2** (Schedule Identity)**.**
*
$C\left(k_{m_{j}}\left(i\right)\right)=m_{j}$
*

**Definition** **A7** (“Powers” of Single Mutators)**.**
*For any mutator function $m_{j}\in M$, $m_{j}^{\left[i\right]}\left(B_{0}\right)={\overset{⋄}{m}}^{k_{j}\left(i\right)}\left(B_{0}\right)$. This means that $m_{j}^{\left[i\right]}\left(B_{0}\right)$ is the result of $k_{j}\left(i\right)$ mutations of $B_{0}$,* exactly *i of which were $m_{j}$-mutations.*

**Proposition** **A3** (Mutator Order)**.**
*
$n_{1}<n_{2}\Rightarrow m^{\left[n_{1}\right]}\left(B_{0}\right)\subset m^{\left[n_{2}\right]}\left(B_{0}\right)$
*

Let $B^{C}={⋃}_{i=0}^{\infty }{\overset{⋄}{m}}^{i}\left(B_{0}\right)$ be the union of all the sets of hyperballs generated by the mutator schedule C. For any given mutator function, we can ask when that mutator function will select a specific hyperball and generate new hyperballs from it, if it ever will. First, we let
  (A2) $${\mathrm{indices}}_{j}\left(b\right)=\left\{i\in N_{1}:s_{j}\left({\overset{⋄}{m}}^{k_{m_{j}}\left(i\right)-1}\left(B_{0}\right)\right)=b\right\}.$$
  From this, we have the following definition.

**Definition** **A8** (Index Function)**.** *For each mutator function $m_{s_{j},g_{j}}\in M$, its* index function* $I\left[m_{s_{j},g_{j}}\right]:B^{C}\to N_{\infty }$ maps a hyperball b to the integer representing the step immediately before it is selected by the corresponding mutator function $m_{s_{j},g_{j}}$, if ever. More formally:*

$$I\left[m_{s_{j},g_{j}}\right]\left(b\right)=\left\{\begin{array}{cc} \min({\mathrm{indices}}_{j}\left(b\right)) & {\mathrm{indices}}_{j}\left(b\right)\neq ⌀,\\ \infty & {\mathrm{indices}}_{j}\left(b\right)=⌀. \end{array}\right.$$

*For brevity, we write $I_{j}$ for $I[m_{s_{j},g_{j}}]$.*

Notice this means that if $I_{j}\left(b\right)\in N$, then $b=s_{j}\left({\overset{⋄}{m}}^{k_{m_{j}}\left(I_{j}\left(b\right)\right)-1}\left(B_{0}\right)\right)$. For the sake of brevity, we introduce the following notational shorthand.

**Definition** **A9** (Notation Abuse)**.**
*
$k_{j}\left(b\right)=k_{m_{j}}\left(I_{j}\left(b\right)\right)$
*

This lets us re-write the previous identity as follows.

**Proposition** **A4** (Selection Index)**.**

$$I_{j}\left(b\right)\in N\Rightarrow b=s_{j}\left({\overset{⋄}{m}}^{k_{j}\left(b\right)-1}\left(B_{0}\right)\right)$$

That $I_{j}\left(b\right)$ is a natural number is a profound requirement for our construction. To codify it as a constraint, we introduce the following definition.

**Definition** **A10** (Balanced Mutator Function)**.** *A mutator function $m_{j}$ is* balanced* with respect to C if and only if*

$$\forall b\in B^{C},\;I\left[m_{j}\right]\left(b\right)\in N.$$

Henceforth, we will assume that all mutators are *balanced*, from which we have the following lemma.

**Lemma** **A1** (Children Index)**.**
*
$g_{j}\left(b\right)\subset m_{j}^{\left[I_{j}\left(b\right)\right]}\left(B_{0}\right)$
*

**Proof.** 

$$\begin{array}{cccc} 1. & m_{j}^{\left[I_{j}\left(b\right)\right]}\left(B_{0}\right) & ={\overset{⋄}{m}}^{k_{j}\left(I_{j}\left(b\right)\right)}\left(B_{0}\right)={\overset{⋄}{m}}^{k_{j}\left(b\right)}\left(B_{0}\right) & (\mathrm{By}\;\mathrm{Def}.\mathrm{A7})\\ 2. & & =C\left(k_{j}\left(b\right)\right)\left({\overset{⋄}{m}}^{k_{j}\left(b\right)-1}\left(B_{0}\right)\right) & (\mathrm{By}\;\mathrm{Def}.\mathrm{A5})\\ 3. & & =m_{j}\left({\overset{⋄}{m}}^{k_{j}\left(b\right)-1}\left(B_{0}\right)\right) & (\mathrm{By}\;\mathrm{Prop}.\mathrm{A2})\\ 4. & & ={\overset{⋄}{m}}^{k_{j}\left(b\right)-1}\left(B_{0}\right)\cup g_{j}\left(s_{j}\left({\overset{⋄}{m}}^{k_{j}\left(b\right)-1}\left(B_{0}\right)\right)\right) & (\mathrm{By}\;\mathrm{Def}.\mathrm{A3})\\ 5. & & ={\overset{⋄}{m}}^{k_{j}\left(b\right)-1}\left(B_{0}\right)\cup g_{j}\left(b\right) & (\mathrm{By}\;\mathrm{Prop}.\mathrm{A4})\\ 6. & & ⊃g_{j}\left(b\right) & \end{array}$$

   □

Thus, all of the $g_{j}$-1-descendants of b will exist in $m_{j}^{\left[I_{j}\left(b\right)\right]}\left(B_{0}\right)$, which is to say, after $k_{j}\left(I_{j}\left(b\right)\right)$ “steps” of the mutation schedule C. But what about the “grandchildren” (2-children), “great-grandchildren” (3-children), and further *n*-descendants by $g_{j}$ of b?

**Definition** **A11** (Powers of Generator Functions)**.** *The set of i-descendants of hyperball b is*

$$g^{i}\left(b\right)=\left\{\begin{array}{cc} \left\{b\right\} & i=0,\\ ⋃\{g\left(b^{\prime }\right):b^{\prime }\in g^{i-1}\left(b\right)\} & i>0. \end{array}\right.$$

Note that this extension is compatible with the original definition of a generator, as 

$$\begin{array}{cc} g(b) & =g^{1}\left(b\right)\\ \; & =⋃\{g\left(b^{\prime }\right):b^{\prime }\in g^{0}\left(b\right)\}\\ \; & =⋃\{g\left(b^{\prime }\right):b^{\prime }\in \left\{b\right\}\}\\ \; & =g(b). \end{array}$$

We can now ask when every *i*-descendant by $g_{j}$ of b will exist, i.e., be generated from its parent.

**Definition** **A12** (Powers of Index Functions)**.**

$$I_{j}^{i}\left(b\right)=\max\left(\left\{I_{j}\left(b^{\prime }\right):b^{\prime }\in g_{j}^{i-1}\left(b\right)\right\}\right),\;i>0$$

**Corollary** **A2** (Index Order)**.**
*
$n_{1}<n_{2}\Rightarrow I_{j}^{n_{1}}\left(b\right)<I_{j}^{n_{2}}\left(b\right)$
*

From this definition we can easily derive the following lemma.

**Lemma** **A2** (Generation Index)**.**
*
$g_{j}^{i}\left(b\right)\subset m_{j}^{\left[I_{j}^{i}\left(b\right)\right]}\left(B_{0}\right)$
*

**Proof.** 

$$\begin{array}{cccc} 1. & & I_{j}^{i}\left(b\right)=\max\left(\left\{I_{j}\left(b^{\prime }\right):b^{\prime }\in g_{j}^{i-1}\left(b\right)\right\}\right) & (\mathrm{Def}.\mathrm{A12})\\ 2. & & \forall b^{\prime }\in g_{j}^{i-1}\left(b\right), & \\ & \;2.a & \;I_{j}\left(b^{\prime }\right)\leq I_{j}^{i}\left(b\right) & (\mathrm{By}\;\mathrm{def}.\;\mathrm{of}\;\max)\\ & \;2.b & \;m_{j}^{\left[I_{j}\left(b^{\prime }\right)\right]}\left(B_{0}\right)\subset m_{j}^{\left[I_{j}^{i}\left(b\right)\right]}\left(B_{0}\right) & (\mathrm{By}\;\mathrm{step}2.a\;\mathrm{and}\;\mathrm{Prop}.\mathrm{A3})\\ & \;2.c & \;g_{j}\left(b^{\prime }\right)\subset m_{j}^{\left[I_{j}\left(b^{\prime }\right)\right]}\left(B_{0}\right)\subset m_{j}^{\left[I_{j}^{i}\left(b\right)\right]}\left(B_{0}\right) & (\mathrm{By}\;\mathrm{Lem}.\mathrm{A1})\\ & \;2.d & \;g_{j}\left(b^{\prime }\right)\subset m_{j}^{\left[I_{j}^{i}\left(b\right)\right]}\left(B_{0}\right) & \\ 3. & & ⋃\left\{g_{j}\left(b^{\prime }\right):b^{\prime }\in g_{j}^{i-1}\left(b\right)\right\}\subset m_{j}^{\left[I_{j}^{i}\left(b\right)\right]}\left(B_{0}\right) & \\ 4. & & g_{j}^{i}\left(b\right)\subset m_{j}^{\left[I_{j}^{i}\left(b\right)\right]}\left(B_{0}\right) & (\mathrm{By}\;\mathrm{Def}.\mathrm{A11}) \end{array}$$

   □

To say more succinctly what this means, we introduce more notational sugar.

**Definition** **A13** (Powers of Notation Abuse)**.**
*
$k_{j}^{i}\left(b\right)=k_{m_{j}}\left(I_{j}^{i}\left(b\right)\right)$
*

Thus, all of $g_{j}^{i}\left(b\right)$ (the $g_{j}$–*i*-descendants of the hyperball b) exist in $m_{j}^{\left[I_{j}^{i}\left(b\right)\right]}\left(B_{0}\right)$, meaning that they exist by the $k_{j}^{i}\left(b\right)$^th^ step of the mutation schedule C. For a set of hyperballs B, when do *all* of their $g_{j}$-*i*-descendants exist?

**Definition** **A14** (Set Generator Function)**.**
*We extend the domain of a generator function to sets of hyperballs by defining*

$$g_{j}^{i}\left(B\right)=⋃\left\{g_{j}^{i}\left(b\right):b\in B\right\}.$$

**Definition** **A15** (Set Index Function)**.**
*
$I_{j}^{i}\left(B\right)=\max\left(\left\{I_{j}^{i}\left(b\right):b\in B\right\}\right)$
*

From these definitions, it is easy to show the following.

**Lemma** **A3** (Set Generation Index)**.**
*
$g_{j}^{i}\left(B\right)\subset m_{j}^{\left[I_{j}^{i}\left(B\right)\right]}\left(B_{0}\right)$
*

**Proof.** 

$$\begin{array}{cccc} 1. & & I_{j}^{i}\left(B\right)=\max\left(\left\{I_{j}^{i}\left(b\right):b\in B\right\}\right) & (\mathrm{Def}.\mathrm{A15})\\ 2. & & \forall b\in B, & \\ & \;2.a & \;I_{j}^{i}\left(b\right)\leq I_{j}^{i}\left(B\right) & (\mathrm{By}\;\mathrm{def}.\;\mathrm{of}\;\max)\\ & \;2.b & \;m_{j}^{\left[I_{j}^{i}\left(b\right)\right]}\left(B_{0}\right)\subset m_{j}^{\left[I_{j}^{i}\left(B\right)\right]}\left(B_{0}\right) & (\mathrm{By}\;\mathrm{Lem}.\mathrm{A3.2.a}\;\mathrm{and}\;\mathrm{Prop}.\mathrm{A3})\\ & \;2.c & \;g_{j}^{i}\left(b\right)\subset m_{j}^{\left[I_{j}^{i}\left(b\right)\right]}\left(B_{0}\right)\subset m_{j}^{\left[I_{j}^{i}\left(B\right)\right]}\left(B_{0}\right) & (\mathrm{By}\;\mathrm{Lem}.\mathrm{A2})\\ & \;2.d & \;g_{j}^{i}\left(b\right)\subset m_{j}^{\left[I_{j}^{i}\left(B\right)\right]}\left(B_{0}\right) & \\ 3. & & ⋃\left\{g_{j}^{i}\left(b\right):b\in B\right\}\subset m_{j}^{\left[I_{j}^{i}\left(B\right)\right]}\left(B_{0}\right) & \\ 4. & & g_{j}^{i}\left(B\right)\subset m_{j}^{\left[I_{j}^{i}\left(B\right)\right]}\left(B_{0}\right) & (\mathrm{By}\;\mathrm{Def}.\mathrm{A14}) \end{array}$$

   □

Which is to say that all of the $m_{j}$-*i*-descendants of every hyperball in B exist in $m_{j}^{\left[I_{j}^{i}\left(B\right)\right]}\left(B_{0}\right)$. Further stretching our notation, $k_{j}^{i}\left(B\right)=k_{m_{j}}\left(I_{j}^{i}\left(B\right)\right)$, meaning that every $m_{j}$-*i*-descendant of every hyperball in B exists by the $k_{j}^{i}\left(B\right)$^th^ step of the mutation schedule C.

### Appendix A.2. Coverage by Extension

Let us now restate the definition of the *extension* function as follows.

**Definition** **A16** (Extension)**.**
*The extension function extend takes in three arguments, a hyperball b and two scaling parameters a>1 and c>1, and returns a new set of hyperballs covering “new” regions of S:*

$$\mathrm{extend}\left(b,a,c\right)=\left\{b^{\left(1\right)},b^{\left(2\right)},\ldots b^{\left(k\right)}\right\}$$

*with the following properties:*

1. *$\forall b^{\prime }\in \mathrm{extend}\left(b,a,c\right),\epsilon \left(b^{\prime }\right)=a\;\cdot \;\epsilon \left(b\right)$,*
2. *$B_{c\;\cdot \;\epsilon (b)}\left(s\left(b\right)\right)\subset b\cup \left(⋃\mathrm{extend}(b,a,c)\right)$.*

For the sake of convenience, we will use $g_{\Delta }\left(b\right)$ for extend(b,a,c), with $m_{\Delta }=m_{s_{\Delta },g_{\Delta }}$. If we keep mutating our initial set of “seed” hyperballs $B_{0}$, how large a region can we cover?

**Definition** **A17** (Family Function)**.** *The union of all descendants of a hyperball b up to the i^th^-generation is referred to as the i*-family* of b:*

$${\hat{g}}_{j}^{i}\left(b\right)=\overset{i}{\underset{l=0}{⋃}}g_{j}^{l}\left(b\right).$$

Notice that ${\hat{g}}_{j}\left(b\right)=\left\{b\right\}\cup g_{j}\left(b\right)$. We denote the area covered by the *i*-family of b as per the following definition.

**Definition** **A18** (Family Area)**.**
*
$A^{i}\left(b\right)=⋃{\hat{g}}_{\Delta }^{i}\left(b\right)$
*

From these definitions, the following proposition is evident.

**Proposition** **A5** (Single Extension Cover)**.**
*
$B_{c\epsilon (b)}\left(s\left(b\right)\right)\subset A^{1}\left(b\right)$
*

Furthermore, we can derive the following relationship.

**Lemma** **A4** (Family Area Recurrence Relation)**.**

$$A^{i+1}\left(b\right)=A^{i}\left(b\right)\cup ⋃\left\{A^{1}\left(b^{\prime }\right):b^{\prime }\in g_{\Delta }^{i}\left(b\right)\right\}$$

**Proof.** 

$$\begin{array}{cccc} 1. & A^{i+1} & =⋃{\hat{g}}_{\Delta }^{i+1}\left(b\right) & (\mathrm{By}\;\mathrm{Def}.\mathrm{A18})\\ 2. & & =⋃\left\{{⋃}_{l=0}^{i+1}g_{\Delta }^{l}\left(b\right)\right\} & (\mathrm{By}\;\mathrm{Def}.\mathrm{A17})\\ 3. & & =⋃\left\{{⋃}_{l=0}^{i}g_{\Delta }^{l}\left(b\right)\right\}\cup \left(⋃g_{\Delta }^{i}\left(b\right)\cup ⋃g_{\Delta }^{i+1}\left(b\right)\right) & \\ 4. & & =⋃{\hat{g}}_{\Delta }^{i}\left(b\right)\cup \left(⋃g_{\Delta }^{i}\left(b\right)\cup ⋃g_{\Delta }^{i+1}\left(b\right)\right) & (\mathrm{By}\;\mathrm{Def}.\mathrm{A17})\\ 5. & & =A^{i}\left(b\right)\cup \left(⋃g_{\Delta }^{i}\left(b\right)\cup ⋃g_{\Delta }^{i+1}\left(b\right)\right) & (\mathrm{By}\;\mathrm{Def}.\mathrm{A18})\\ 6. & & =A^{i}\left(b\right)\cup ⋃\left\{b^{\prime }\cup ⋃g_{\Delta }\left(b^{\prime }\right):b^{\prime }\in g_{\Delta }^{i}\left(b\right)\right\} & \\ 7. & & =A^{i}\left(b\right)\cup ⋃\left\{⋃{\hat{g}}_{\Delta }\left(b^{\prime }\right):b^{\prime }\in g_{\Delta }^{i}\left(b\right)\right\} & (\mathrm{By}\;\mathrm{Def}.\mathrm{A17})\\ 8. & & =A^{i}\left(b\right)\cup ⋃\left\{⋃A^{1}\left(b^{\prime }\right):b^{\prime }\in g_{\Delta }^{i}\left(b\right)\right\} & (\mathrm{By}\;\mathrm{Def}.\mathrm{A18}) \end{array}$$

   □ Now, letting ∂X be the *boundary* for any set *X*, consider the following.

**Proposition** **A6** (Family Area Boundary)**.**
*
$\partial A^{i}\left(b\right)\subset ⋃g_{\Delta }^{i}\left(b\right)$
*

*This follows from the fact that $\forall i\in N,{\hat{g}}_{\Delta }^{i-1}\left(b\right)\subset {\hat{g}}_{\Delta }^{i}\left(b\right)$.*

Before we go on, we need to introduce the following operation:

**Definition** **A19** (Dilation)**.**
*
$D_{r}\left(X\right)=⋃\left\{B_{r}\left(s\right):s\in X\right\}$
*

as well as several propositions about it.

**Proposition** **A7** (Boundary Dilation)**.**
*
$X\cup D_{r}\left(\partial X\right)=D_{r}\left(X\right)$
*

**Proposition** **A8** (Hyperball Dilation)**.**

$$D_{r_{2}}\left(B_{r_{1}}\left(s\right)\right)=B_{r_{1}+r_{2}}\left(s\right)$$

**Proposition** **A9** (Dilation Preserves Subsets)**.**

$$X\subset Y\Rightarrow D_{r}\left(X\right)\subset D_{r}\left(Y\right)$$

Now, we can return to the matter of the family area. Notice that

**Proposition** **A10** (Extension Hyperball Size)**.**
*$\forall b^{\prime }\in g_{\Delta }^{i}\left(b\right),\;\epsilon \left(b^{\prime }\right)=a^{i}\;\cdot \;\epsilon \left(b\right)$.*

For convenience, we will write $\epsilon_{b}$ for ϵ(b), meaning that $\forall b^{\prime }\in g_{\Delta }^{i}\left(b\right),\;\epsilon_{b^{\prime }}=a^{i}\;\cdot \;\epsilon_{b}$.

**Lemma** **A5** (Area Lower Limit)**.**
*
$A^{i+1}⊃D_{a^{i}\epsilon_{b}\left(c-1\right)}\left(A^{i}\left(b\right)\right)$
*

**Proof.** 

$$\begin{array}{ccccc} 1. & & A^{i+1} & =A^{i}\left(b\right)\cup ⋃\left\{A^{1}\left(b^{\prime }\right):b^{\prime }\in g_{\Delta }^{i}\left(b\right)\right\} & (\mathrm{By}\;\mathrm{Lem}.\mathrm{A4})\\ 2. & & & ⊃A^{i}\left(b\right)\cup ⋃\left\{B_{c\epsilon_{b^{\prime }}}\left(s_{b^{\prime }}\right):b^{\prime }\in g_{\Delta }^{i}\left(b\right)\right\} & (\mathrm{By}\;\mathrm{Prop}.\mathrm{A5})\\ & \;2.a & & c\epsilon_{b^{\prime }}=c\epsilon_{b^{\prime }}-\epsilon_{b^{\prime }}+\epsilon_{b^{\prime }} & \\ & \;2.b & & \;\;\;\;\;\;\;=\epsilon_{b^{\prime }}\left(c-1\right)+\epsilon_{b^{\prime }} & \\ 3. & & & ⊃A^{i}\left(b\right)\cup ⋃\left\{B_{\epsilon_{b^{\prime }}\left(c-1\right)+\epsilon_{b^{\prime }}}\left(s_{b^{\prime }}\right):b^{\prime }\in g_{\Delta }^{i}\left(b\right)\right\} & \\ 4. & & & ⊃A^{i}\left(b\right)\cup ⋃\left\{D_{\epsilon_{b^{\prime }}\left(c-1\right)}\left(B_{\epsilon_{b^{\prime }}}\left(s_{b^{\prime }}\right)\right):b^{\prime }\in g_{\Delta }^{i}\left(b\right)\right\} & (\mathrm{By}\;\mathrm{Prop}.\mathrm{A8})\\ 5. & & & ⊃A^{i}\left(b\right)\cup ⋃\left\{D_{\epsilon_{b^{\prime }}\left(c-1\right)}\left(b^{\prime }\right):b^{\prime }\in g_{\Delta }^{i}\left(b\right)\right\} & \\ 6. & & & ⊃A^{i}\left(b\right)\cup D_{\epsilon_{b^{\prime }}\left(c-1\right)}\left(⋃g_{\Delta }^{i}\left(b\right)\right) & \\ 7. & & & ⊃A^{i}\left(b\right)\cup D_{\epsilon_{b^{\prime }}\left(c-1\right)}\left(\partial A^{i}\left(b\right)\right) & (\mathrm{By}\;\mathrm{Prop}.\mathrm{A6}\mathrm{and}\mathrm{A9})\\ 8. & & & ⊃D_{\epsilon_{b^{\prime }}\left(c-1\right)}\left(A^{i}\left(b\right)\right) & (\mathrm{By}\;\mathrm{Prop}.\mathrm{A7})\\ 9. & & & ⊃D_{a^{i}\epsilon_{b}\left(c-1\right)}\left(A^{i}\left(b\right)\right) & (\mathrm{By}\;\mathrm{Prop}.\mathrm{A10}) \end{array}$$

   □

**Lemma** **A6** (Extension Coverage Radius)**.**

$$\forall i\in N,\;B_{R_{i}}\left(s_{b}\right)\subset A^{i}\left(b\right),\;\mathrm{with}\;R_{i}=\epsilon_{b}+\epsilon_{b}\left(c-1\right)\frac{a^{i}-1}{a-1}$$

**Proof.** We will prove by induction.

$$\begin{array}{cccc} 1. & A^{0}\left(b\right) & ={⋃}_{l=0}^{0}g_{\Delta }^{l}\left(b\right) & (\mathrm{By}\;\mathrm{Def}.\mathrm{A17}\mathrm{and}\mathrm{A18})\\ 2. & & =b=B_{\epsilon_{b}}\left(s_{b}\right) & \end{array}$$

The base-case: let $R_{0}=\epsilon_{b}$.

$$\begin{array}{ccc} 3. & B_{R_{0}}\left(s_{b}\right) & \subset A^{0}\left(b\right) \end{array}$$

The inductive-step: $R_{i+1}=R_{i}+a^{i}R_{0}\left(c-1\right)$. Assume that $B_{R_{i}}\left(s_{b}\right)\subset A^{i}\left(b\right)$.

$$\begin{array}{cccc} 4. & B_{R_{i}}\left(s_{b}\right) & \subset A^{i}\left(b\right) & (\mathrm{By}\;\mathrm{Assumption})\\ 5. & D_{a^{i}\epsilon_{b}\left(c-1\right)}\left(B_{R_{i}}\left(s_{b}\right)\right) & \subset D_{a^{i}\epsilon_{b}\left(c-1\right)}\left(A^{i}\left(b\right)\right) & (\mathrm{By}\;\mathrm{Prop}.\mathrm{A9})\\ 6. & B_{R_{i}+a^{i}\epsilon_{b}\left(c-1\right)}\left(s_{b}\right) & \subset D_{a^{i}\epsilon_{b}\left(c-1\right)}\left(A^{i}\left(b\right)\right) & (\mathrm{By}\;\mathrm{Prop}.\mathrm{A8})\\ 7. & B_{R_{i}+a^{i}\epsilon_{b}\left(c-1\right)}\left(s_{b}\right) & \subset A^{i+1}\left(b\right) & (\mathrm{By}\;\mathrm{Lem}.\mathrm{A5})\\ 8. & B_{R_{i+1}}\left(s_{b}\right) & \subset A^{i+1}\left(b\right) & \\ 9. & B_{R_{i}}\left(s_{b}\right)\subset A^{i}\left(b\right) & \Rightarrow B_{R_{i+1}}\left(s_{b}\right)\subset A^{i+1}\left(b\right) & (\mathrm{By}\;\mathrm{Lem}.\mathrm{A6.4}\mathrm{and}\mathrm{A6.8})\\ 10. & & \;\forall i\in N,\;B_{R_{i}}\left(s_{b}\right)\subset A^{i}\left(b\right) & (\mathrm{By}\mathrm{induction}) \end{array}$$

For the derivation of $R_{i}$.

$$\begin{array}{ccc} 11. & R_{i} & =R_{i-1}+a^{i-1}\epsilon_{b}\left(c-1\right)\\ 12. & & =\epsilon_{b}+\sum_{n=0}^{i-1}\epsilon_{b}\left(c-1\right)a^{n}\\ 13. & & =\epsilon_{b}+\epsilon_{b}\left(c-1\right)\sum_{n=0}^{i-1}a^{n}\\ 14. & & =\epsilon_{b}+\epsilon_{b}\left(c-1\right)\frac{a^{i}-1}{a-1} \end{array}$$

   □

Now, for a given radius *R*, we can calculate how many generations it will take to match or exceed that radius, $i_{\Delta }^{b}\left(R\right)$.
  $$\begin{array}{cc} \epsilon_{b}+\epsilon_{b}\left(c-1\right)\frac{a^{i}-1}{a-1} & =R\\ \epsilon_{b}\left(c-1\right)\frac{a^{i}-1}{a-1} & =R-\epsilon_{b}\\ \frac{a^{i}-1}{a-1} & =\frac{R-\epsilon_{b}}{\epsilon_{b}\left(c-1\right)}\\ a^{i}-1 & =\frac{R-\epsilon_{b}}{\epsilon_{b}\left(c-1\right)}\left(a-1\right)\\ a^{i} & =\frac{R-\epsilon_{b}}{\epsilon_{b}\left(c-1\right)}\left(a-1\right)+1\\ i & =\log_{a}\left(\frac{\left(R-\epsilon_{b}\right)\left(a-1\right)}{\epsilon_{b}\left(c-1\right)}+1\right) \end{array}$$
  The actual number of generations must be an integer as big or bigger than *i*, which we denote as follows:
  (A3) $$\begin{array}{cc} i_{\Delta }^{b}\left(R\right) & =\left\{\begin{array}{cc} 0, & 0\leq R\leq \epsilon_{b},\\ \left\lceil \log_{a}\left(\frac{\left(R-\epsilon_{b}\right)\left(a-1\right)}{\epsilon_{b}\left(c-1\right)}+1\right)\right\rceil , & R>\epsilon_{b}. \end{array}\right. \end{array}$$
  By construction then, we have the following.

**Proposition** **A11** (Radius Inequality)**.**
*
$R\leq R_{i_{\Delta }^{b}\left(R\right)}$
*

From this we, can show the following lemma.

**Lemma** **A7** (Point Coverage)**.**

$$s\in S,\;b\in O_{S}\Rightarrow s\in A^{i_{\Delta }^{b}(∥s-s_{b}∥)}\left(b\right)$$

**Proof.** 

$$\begin{array}{cccc} 1. & s & \in B_{∥s-s_{b}∥}\left(s_{b}\right) & \\ 2. & & \in B_{∥s-s_{b}∥}\left(s_{b}\right)\subset B_{R_{i_{\Delta }^{b}(∥s-s_{b}∥)}}\left(s_{b}\right) & (\mathrm{By}\;\mathrm{Prop}.\mathrm{A11})\\ 3. & & \in B_{R_{i_{\Delta }^{b}(∥s-s_{b}∥)}}\left(s_{b}\right)\subset A^{i_{\Delta }^{b}(∥s-s_{b}∥)}\left(b\right) & (\mathrm{By}\;\mathrm{Lem}.\mathrm{A6})\\ 4. & & \in A^{i_{\Delta }^{b}(∥s-s_{b}∥)}\left(b\right) & \end{array}$$

   □

This means that for any starting hyperball b, any arbitrary point s will be covered by b’s *i*-family when $i=i_{\Delta }^{b}(∥s-s_{b}∥)$. When will every point (sub-goal) in a plan s be covered by b’s *i*-family? Each point s∈s will have a (potentially different) “coverage” generation, $i_{\Delta }^{b}(∥s-s_{b}∥)$. The maximum of these integers will correspond to the generation that covers *all* of the points in s.
  (A4) $$i_{\Delta }^{b}\left(s\right)=\max(\{i_{\Delta }^{b}(∥s-s_{b}∥):s\in s\})$$
  By construction, ∀s∈s, $s\in A^{i_{\Delta }^{b}\left(s\right)}\left(b\right)$, so by definition, we have the following.

**Proposition** **A12** (Hyperball Family Plan Coverage)**.**

∀s∈SS*and∀b∈OS,s⊂←.AiΔb(s)(b)

Consider again our arbitrary but fixed set of “seed” hyperballs, $B_{0}$. We know by the previous proposition that for any plan s and hyperball $b\in B_{0}$, s⊂←.AiΔb(s). In the course of $B_{0}$ being mutated, for each hyperball $b\in B_{0}$ this will occur, per A2, after $I_{\Delta }^{i_{\Delta }^{b}\left(s\right)}\left(b\right)$ applications of the extension function. The first time this occurs for *any* of the hyperballs, s will be covered; but for the ease of the proof, we will be conservative and let

(A5) $$b^{⋆}=\underset{b\in B_{0}}{\mathrm{argmin}}I_{\Delta }^{i_{\Delta }^{b}\left(s\right)}\left(b\right)\;\mathrm{and}\;I_{\Delta }^{⋆}\left(s\right)=I_{\Delta }^{i_{\Delta }^{b^{⋆}}\left(s\right)}\left(B_{0}\right).$$

Letting $k_{\Delta }^{⋆}\left(s\right)=k_{\Delta }\left(I_{\Delta }^{⋆}\left(s\right)\right)$, notice the following.

**Lemma** **A8.**
*
$A^{i_{\Delta }^{b^{⋆}}\left(s\right)}\left(b^{⋆}\right)\subset ⋃{\overset{⋄}{m}}^{k_{\Delta }^{⋆}\left(s\right)}\left(B_{0}\right)$
*

**Proof.** 

$$\begin{array}{cccc} 1. & A^{i_{\Delta }^{b^{⋆}}\left(s\right)}\left(b^{⋆}\right) & \subset ⋃{\hat{g}}_{\Delta }^{i_{\Delta }^{b^{⋆}}\left(s\right)}\left(b^{⋆}\right) & (\mathrm{By}\;\mathrm{Def}.\mathrm{A18})\\ 2. & ⋃{\hat{g}}_{\Delta }^{i_{\Delta }^{b^{⋆}}\left(s\right)}\left(b^{⋆}\right) & \subset ⋃{\hat{g}}_{\Delta }^{i_{\Delta }^{b^{⋆}}\left(s\right)}\left(B_{0}\right) & (\mathrm{By}\;\mathrm{Def}.\mathrm{A14})\\ 3. & ⋃{\hat{g}}_{\Delta }^{i_{\Delta }^{b^{⋆}}\left(s\right)}\left(B_{0}\right) & \subset ⋃m_{\Delta }^{\left[I_{\Delta }^{⋆}\left(s\right)\right]}\left(B_{0}\right) & (\mathrm{By}\;\mathrm{Lem}.\mathrm{A3})\\ 4. & ⋃m_{\Delta }^{\left[I_{\Delta }^{⋆}\left(s\right)\right]}\left(B_{0}\right) & \subset ⋃{\overset{⋄}{m}}^{k_{\Delta }^{⋆}\left(s\right)}\left(B_{0}\right) & (\mathrm{By}\;\mathrm{Def}.\mathrm{A7}) \end{array}$$

   □

From which we have the following theorem.

**Theorem** **A1** (Behavior Coverage by Extension)**.** *If $\overset{⋄}{m}$ is a *fair* polymutator function and $m_{\Delta }$ is a *balanced* mutator function, then*

s∈SS*⇒s⊂←.m⋄kΔ⋆(s)(B0)

**Proof.** 

1.s⊂←.AiΔb⋆(s)(b⋆)(ByEquation (A5)andProp.A12)2.s⊂←.m⋄kΔ⋆(s)(B0)(ByLem.A8)

   □

### Appendix A.3. Precision by Refinement

For a given behavior s, imagine that we have a set of hyperballs $B_{\Delta }={\overset{⋄}{m}}^{k_{\Delta }^{⋆}\left(s\right)}\left(B_{0}\right)$, so that s⊂←.BΔ. Notice that s⥺B does not imply that $\exists b\in B_{B_{\Delta }}^{*}$ such that s∈⟦b⟧, and even if there *does* exist such a $b\in B_{B_{\Delta }}^{*}$, it does not imply that $r_{K}\left(b\right)=1$. However, we can *refine* hyperballs in $B_{\Delta }$ to fix this problem. Briefly, let us restate the definition of refinement.

**Definition** **A20** (Refinement)**.**
*The refinement function refine takes in two arguments, a hyperball b and a scaling parameter a∈(0,1), and returns a set of new “denser” hyperballs:*

$$\mathrm{refine}\left(b,a\right)=\left\{b^{\left(1\right)},b^{\left(2\right)},\ldots b^{\left(k\right)}\right\}$$

*with the following properties:*

1. *$s\left(b^{\left(1\right)}\right)=s\left(b\right)$,*
2. *$\forall b^{\prime }\in \mathrm{refine}\left(b,a\right),\epsilon \left(b^{\prime }\right)=a\;\cdot \;\epsilon \left(b\right)$,*
3. *b⊂⋃refine(b,a).*

For the sake of convenience, we will use $g_{▿}\left(b\right)$ for refine(b,a), with $m_{▿}=m_{s_{▿},g_{▿}}$. Notice also that $b\subset ⋃g_{▿}\left(b\right)$. From this, we can derive the following.

**Proposition** **A13** (Refinement Generation Coverage)**.**

$$\forall i\in N,\;⋃g_{▿}^{i}\left(B\right)\subset ⋃g_{▿}^{i+1}\left(B\right)$$

**Proof.** 

$$\begin{array}{cccc} 1. & g_{▿}^{i+1}\left(B\right) & =⋃\{g_{▿}^{i+1}\left(b\right):b\in B\} & (\mathrm{By}\;\mathrm{Def}.\mathrm{A14})\\ 2. & & =⋃\{⋃\left\{g_{▿}\left(b^{\prime }\right):b^{\prime }\in g_{▿}^{i}\left(b\right)\right\}:b\in B\} & (\mathrm{By}\;\mathrm{Def}.\mathrm{A11})\\ 3. & & =⋃\{g_{▿}\left(b\right):b\in g_{▿}^{i}\left(B\right)\} & \\ 4. & ⋃g_{▿}^{i+1}\left(B\right) & =⋃⋃\{g_{▿}\left(b\right):b\in g_{▿}^{i}\left(B\right)\} & \\ 5. & & =⋃\{⋃g_{▿}\left(b\right):b\in g_{▿}^{i}\left(B\right)\} & \\ 6. & & ⊃⋃\{b:b\in g_{▿}^{i}\left(B\right)\} & (\mathrm{By}\;\mathrm{Def}.\mathrm{A20.3})\\ 7. & & ⊃⋃g_{▿}^{i}\left(B\right) & \end{array}$$

   □

From this, the next proposition follows by induction.

**Proposition** **A14** (Refinement Progenitor Coverage)**.**

$$\forall i\in N,\;⋃B\subset ⋃g_{▿}^{i}\left(B\right)$$

We can then see the next proposition from the definition of $g_{▿}$.

**Proposition** **A15** (Refinement Scaling)**.**

$$\forall b^{\prime }\in g_{▿}^{i}\left(b\right),\;\epsilon \left(b^{\prime }\right)=a^{i}\;\cdot \;\epsilon \left(b\right)$$

Which is to say that after *i* generations, all hyperballs produced by refinement in the *i*-generation from b will have a radius $a^{i}$-times as big as that of b. How many generations have to pass until the hyperballs produced have a radius as small or smaller than a given value *r*?

$$\begin{array}{cc} \epsilon_{b}\;\cdot \;a^{i} & =r\\ a^{i} & =\frac{r}{\epsilon_{b}}\\ i & =\log_{a}\left(\frac{r}{\epsilon_{b}}\right) \end{array}$$

The actual number of generations must be an integer as big or bigger than *i*, which we denote as

(A6) $$\begin{array}{cc} i_{▿}^{b}\left(r\right) & =\max\{0,\left\lceil \log_{a}\left(\frac{r}{\epsilon_{b}}\right)\right\rceil \}. \end{array}$$

For a set of hyperballs B, each hyperball b in the set will require a different number of generations to reach a stage where all descendants in a generation have a radius smaller than *r*, as shown formally below.

**Lemma** **A9.**
*
$\forall b^{\prime }\in g_{▿}^{i_{▿}^{b}\left(r\right)}\left(b\right),\;\epsilon \left(b^{\prime }\right)\leq r$
*

**Proof.** 

$$\begin{array}{cccc} 1. & \forall b^{\prime }\in g_{▿}^{i_{▿}^{b}\left(r\right)}\left(b\right),\;\epsilon \left(b^{\prime }\right) & =a^{i_{▿}^{b}\left(r\right)}\;\cdot \;\epsilon \left(b\right) & (\mathrm{By}\;\mathrm{Prop}.\mathrm{A15})\\ 2. & & \leq a^{\left\lceil \log_{a}\left(\frac{r}{\epsilon_{b}}\right)\right\rceil }\;\cdot \;\epsilon_{b} & (\mathrm{By}\;\mathrm{Equation}\;(\mathrm{A6}))\\ 3. & & \leq a^{\log_{a}\left(\frac{r}{\epsilon_{b}}\right)}\;\cdot \;\epsilon_{b} & (\mathrm{By}\;a<1)\\ 4. & & \leq \frac{r}{\epsilon_{b}}\;\cdot \;\epsilon_{b} & \\ 5. & & \leq r & \end{array}$$

   □

From this, it also follows that

(A7) $$\forall n\geq i_{▿}^{b}\left(r\right),\;\forall b^{\prime }\in g_{▿}^{n}\left(b\right),\;\epsilon \left(b^{\prime }\right)\leq r.$$

The number of generations after which they *all* have descendants of radius less than *r* is given by

(A8) $$i_{▿}^{B}\left(r\right)=\max\left(\left\{i_{▿}^{b}\left(r\right):b\in B\right\}\right).$$

Using this definition, we can derive the following lemma.

**Lemma** **A10** (Refinement Radius)**.**
*
$\forall b\in g_{▿}^{i_{▿}^{B}\left(r\right)}\left(B\right),\;\epsilon \left(b\right)\leq r$
*

**Proof.** 

$$\begin{array}{cccc} 1. & i_{▿}^{B}\left(r\right) & =\max(\left\{i_{▿}^{b}\left(r\right):b\in B\right\}) & (\mathrm{By}\;\mathrm{Equation}\;(\mathrm{A8}))\\ 2. & \forall b\in B,\;i_{▿}^{b}\left(r\right) & \leq i_{▿}^{B}\left(r\right) & \\ 3. & \forall b\in g_{▿}^{i_{▿}^{b}\left(r\right)}\left(b\right),\;\epsilon \left(b\right) & \leq r & (\mathrm{By}\;\mathrm{Lem}.\mathrm{A9})\\ 4. & \forall b\in g_{▿}^{i_{▿}^{B}\left(r\right)}\left(B\right),\;\epsilon \left(b\right) & \leq r & (\mathrm{By}\;\mathrm{Equation}\;(\mathrm{A7})) \end{array}$$

   □

Here, the notation begins to get a little heavier, so we introduce the following definitions.

**Definition** **A21** (Refinement Index)**.**
*
$I_{▿}^{⋆}\left(B,r\right)=I_{▿}^{i_{▿}^{B}\left(r\right)}\left(B\right)$
*

**Definition** **A22** (Polymutator Refinement Index)**.**
*
$k_{▿}^{⋆}\left(B,r\right)=k_{m_{▿}}\left(I_{▿}^{⋆}\left(B,r\right)\right)$
*

By Lemma A3, we have the following.

**Definition** **A23** (Refined Hyperball Set)**.**
*
$B_{▿}\left(B,r\right)={\overset{⋄}{m}}^{k_{▿}^{⋆}\left(B,r\right)}\left(B_{0}\right)$
*

**Lemma** **A11** (Sub-Hyperballs)**.**

$$b=B_{\epsilon }\left(s\right),\;b^{\prime }=B_{\epsilon^{\prime }}\left(s^{\prime }\right),\;\epsilon^{\prime }\leq \frac{\epsilon }{2},\;s\in b^{\prime }\Rightarrow b^{\prime }\subset b$$

**Proof.** 

$$\begin{array}{cccc} 1. & ∥s-s^{\prime }∥ & \leq \frac{\epsilon }{2} & \left(\mathrm{Given}\right)\\ 2. & \forall s^{\prime \prime }\in b^{\prime },\;∥s^{\prime \prime }-s^{\prime }∥ & \leq \frac{\epsilon }{2} & \\ 3. & ∥s-s^{\prime }∥+∥s^{\prime \prime }-s^{\prime }∥ & \leq \frac{\epsilon }{2}+\frac{\epsilon }{2}=\epsilon & \\ 4. & ∥s^{\prime \prime }-s∥\leq ∥s-s^{\prime }∥+∥s^{\prime \prime }-s^{\prime }∥ & \leq \epsilon & (\mathrm{Triangle}\;\mathrm{Ineq}.)\\ 5. & ∥s^{\prime \prime }-s∥ & \leq \epsilon & \\ 6. & s^{\prime \prime }\in b^{\prime } & \Rightarrow s^{\prime \prime }\in b & \\ 7. & b^{\prime } & \subset b & \end{array}$$

   □

**Lemma** **A12** (Arbitrary Refinement Precision)**.**

$$\forall s\in S,\forall r>0\;;\;s\in ⋃B\Rightarrow \exists b\in \;B_{▿}\left(B,\frac{r}{2}\right)\;s.t.\;s\in b,b\subset B_{r}\left(s\right)$$

**Proof.** 

$$\begin{array}{cccc} 1. & s & \in ⋃B & \left(\mathrm{Given}\right)\\ 2. & & \in ⋃B\subset ⋃g_{▿}^{i_{▿}^{B}\left(\frac{r}{2}\right)}\left(B\right) & (\mathrm{By}\;\mathrm{Prop}.\mathrm{A14})\\ 3. & \exists b\in g_{▿}^{i_{▿}^{B}\left(\frac{r}{2}\right)}\left(B\right):s & \in b & \\ 4. & \epsilon (b) & \leq \frac{r}{2} & (\mathrm{By}\;\mathrm{Lem}.\mathrm{A10})\\ 5. & b & \subset B_{r}\left(s\right) & (\mathrm{By}\;\mathrm{Lem}.\mathrm{A11})\\ 6. & g_{▿}^{i_{▿}^{B}\left(\frac{r}{2}\right)}\left(B\right) & \subset m_{▿}^{\left[I_{▿}^{⋆}\left(B,\frac{r}{2}\right)\right]}\left(B_{0}\right)={\overset{⋄}{m}}^{k_{▿}^{⋆}\left(B,\frac{r}{2}\right)}\left(B_{0}\right)=B_{▿}\left(B,\frac{r}{2}\right) & \\ 7. & b & \in B_{▿}\left(B,\frac{r}{2}\right) & \end{array}$$

   □

**Lemma** **A13** (Path Containment)**.**

$$\forall s\in S_{S}^{*},\forall r>0\;;\;s⥺B\Rightarrow \exists b\in B_{B_{▿}\left(B,\frac{r}{2}\right)}^{*}\;s.t.\;s\in ⟦b⟧,⟦b⟧\subset N_{r}\left(s\right)$$

**Proof.** 

$$\begin{array}{cccc} 1. & s⥺B & \Rightarrow \forall s\in s,s\in ⋃B & \\ 2. & & \Rightarrow \forall s\in s,\exists b\in B_{▿}\left(B,\frac{r}{2}\right):s\in b,b\subset B_{r}\left(s\right) & (\mathrm{By}\;\mathrm{Lem}.\mathrm{A12}) \end{array}$$

Let b(s) be such a hyperball for each s∈s, and let $b={\left(b\left(s\right)\right)}_{s\in s}$.

$$\begin{array}{ccc} 3. & \forall b & \in b,b\in B_{▿}\left(B,\frac{r}{2}\right)\\ 4. & b & \in B_{B_{▿}\left(B,\frac{r}{2}\right)}^{*}\\ 5. & s & \in ⟦b⟧\\ 6. & ⟦b⟧ & \subset N_{r}\left(s\right) \end{array}$$

   □

### Appendix A.4. Density and Countability

Now, we can use the preceding definitions and facts to construct a special set of behaviors.

**Definition** **A24** (Polymutator Behavior Set)**.** *The* polymutator behavior set* is*

$$S_{\overset{⋄}{m}}^{*}=\underset{i\in N}{⋃}S_{S({\overset{⋄}{m}}^{i}\left(B_{0}\right))}^{*}$$

We have analogized this set to the *rational numbers* and the set of all behaviors $S_{S}^{*}$ to the *real numbers*. For each fixed plan length *n*, equip $S_{S}^{n}$ with the metric

$$d_{n}\left(s,s^{\prime }\right)=\max_{0\leq i\leq n}∥s\left[i\right]-s^{\prime }\left[i\right]∥,$$

and equip $S_{S}^{*}={⨆}_{n\geq 1}S_{S}^{n}$ with the corresponding disjoint-union topology. For a scalar r>0, $N_{r}\left(s\right)$ denotes the tube obtained by assigning radius *r* to every waypoint. All closures and density statements below use this topology. We can give a formal definition of a *rational set of behaviors*.

**Definition** **A25** (Rational Set of Behaviors)**.** *A set of behaviors S is* rational *if and only if:*

$$\begin{array}{cccc} 1. & \left|S\right| & ={ℵ}_{0} & (\mathrm{the}\;\mathrm{set}\;\mathrm{is}\;\mathrm{countably}\;\mathrm{infinite}).\\ 2. & \bar{S} & =S_{S}^{*} & (\mathrm{the}\;\mathrm{closure}\;\mathrm{of}\;\mathrm{the}\;\mathrm{set}\;\mathrm{is}\;\mathrm{equal}\;\mathrm{to}\;\mathrm{the}\;\mathrm{set}\;\mathrm{of}\;\mathrm{all}\;\mathrm{behaviors},\\ & & & \mathrm{that}\;\mathrm{is}\;\mathrm{to}\;\mathrm{say},\mathrm{it}\;\mathrm{is}\;\mathrm{dense}\;\mathrm{in}\;\mathrm{the}\;\mathrm{set}\;\mathrm{set}\;\mathrm{of}\;\mathrm{all}\;\mathrm{behaviors}). \end{array}$$

It may be obvious, but for the sake of completeness we will prove that the polymutator behavior set is a rational set of behaviors. First, we show that it is countably infinite.

**Lemma** **A14** (Countability of the Polymutator Behavior Set)**.**
*
$\left|S_{\overset{⋄}{m}}^{*}\right|={ℵ}_{0}$
*

**Proof.** 

$$\begin{array}{cccc} 1. & \left|S_{\overset{⋄}{m}}^{*}\right| & =|⋃\{S_{S({\overset{⋄}{m}}^{i}\left(B_{0}\right))}^{*}:i\in N\}| & (\mathrm{By}\;\mathrm{Def}.\mathrm{A24}) \end{array}$$

Let $n_{i}=\left|S\left({\overset{⋄}{m}}^{i}\left(B_{0}\right)\right)\right|$, note that $n_{i}\in N$.

$$\begin{array}{ccc} 2. & |S_{S({\overset{⋄}{m}}^{i}\left(B_{0}\right))}^{*}| & =\sum_{j=2}^{n_{i}}\frac{n_{i}!}{(n_{i}-j)!}\\ 3. & & \in N \end{array}$$

A union of countably infinitely many countable sets is countably infinite, therefore:

$$\begin{array}{ccc} 4. & \left|S_{\overset{⋄}{m}}^{*}\right| & ={ℵ}_{0}. \end{array}$$

   □

Now, we show that it is dense in $S_{S}^{*}$. To do this, we have to re-arrange our picture of $S_{\overset{⋄}{m}}^{*}$, which we have constructed by interleaving going “out” (increasing coverage) with going “in” (increasing resolution). We begin by introducing a function that returns the index after which given plan is guaranteed to be covered by hyperballs of less than a given radius.

**Definition** **A26** (Hyperball Counting)**.**
*
$\psi \left(B_{0},s,r\right)=k_{▿}^{⋆}\left({\overset{⋄}{m}}^{k_{\Delta }^{⋆}\left(s\right)}\left(B_{0}\right),r\right)$
*

From this, we can define the set of corresponding paths.

**Definition** **A27** (Sufficient Path Set)**.**
*
$B_{\psi (B_{0},s,r)}^{*}=B_{{\overset{⋄}{m}}^{\psi (B_{0},s,r)}\left(B_{0}\right)}^{*}$
*

**Theorem** **A2** (Plan Possibility Index)**.** *If $\overset{⋄}{m}$ is a *fair polymutator *function and if $m_{\Delta }$ and $m_{▿}$ are *balanced mutator* functions, then $\forall B_{0}\in P_{\mathrm{fin}}^{+}\left(O_{S}\right),\forall s\in S_{S}^{*},\forall r>0$:*

$$\exists b\in B_{\psi (B_{0},s,r)}^{*}:s\in ⟦b⟧,⟦b⟧\subset N_{r}\left(s\right),\psi \left(B_{0},s,r\right)\in N$$

**Proof.** 

$$\begin{array}{cccc} 1. & s & ⥺{\overset{⋄}{m}}^{k_{\Delta }^{⋆}\left(s\right)}\left(B_{0}\right) & (\mathrm{By}\;\mathrm{Thm}.\mathrm{A1})\\ 2. & \exists b\in B_{\psi (B_{0},s,r)}^{*}:s & \in ⟦b⟧,⟦b⟧\subset N_{r}\left(s\right) & (\mathrm{By}\;\mathrm{Lem}.\mathrm{A13})\\ 3. & \psi (B_{0},s,r) & \in N & (\mathrm{By}\;\mathrm{Def}.\;\mathrm{A8},\;\mathrm{A6},\;\mathrm{A10},\;\mathrm{A22}) \end{array}$$

   □

This simply tells us at what point the set of paths contains a path that “hugs” a plan arbitrarily tightly. Framed in terms of paths, it is not very helpful to us, but for every path b there is a corresponding plan $s_{b}$, which is the plan of corresponding waypoints. We denote this set as $S_{\psi (B_{0},s,r)}^{*}=\left\{s_{b}:b\in B_{\psi (B_{0},s,r)}^{*}\right\}$. Using this, we can show that $S_{\overset{⋄}{m}}^{*}$ is dense in $S_{S}^{*}$. To do this, we construct (a subset of) $S_{\overset{⋄}{m}}^{*}$ in terms of “slices” of behavior space that have the same resolution, by introducing the following definitions.

**Definition** **A28** (Behavior Resolution)**.**

$$\Omega \left(s,r\right)=s^{\prime }\;\mathrm{such}\;\mathrm{that}\;s^{\prime }\in S_{\psi (B_{0},s,r)}^{*}:s\in N_{\frac{r}{2}}\left(s^{\prime }\right),s^{\prime }\in N_{r}\left(s\right)$$

**Definition** **A29** (Behavior Resolution Slice)**.**

$$\Omega [S_{\overset{⋄}{m}}^{*},r]=\{\Omega (s,r):s\in S_{S}^{*}\}$$

From this definition, it should be clear that $\Omega [S_{\overset{⋄}{m}}^{*},r]\subset S_{\overset{⋄}{m}}^{*}$. While this set is obviously not enough to reconstruct the entirety of $S_{\overset{⋄}{m}}^{*}$, we at least know that ${⋃}_{n\in N}\Omega \left[S_{\overset{⋄}{m}}^{*},\frac{1}{n}\right]\subset S_{\overset{⋄}{m}}^{*}$. We can use this set to cleanly show the density of $S_{\overset{⋄}{m}}^{*}$.

**Lemma** **A15** (Density of the Polymutator Behavior Set)**.**
*
$\bar{S_{\overset{⋄}{m}}^{*}}=S_{S}^{*}$
*

**Proof.** 

$$\begin{array}{cccc} 1. & \Omega [S_{\overset{⋄}{m}}^{*},r] & =\{\Omega (s,r):s\in S_{S}^{*}\} & (\mathrm{By}\;\mathrm{Def}.\mathrm{A29})\\ 2. & \Omega [S_{\overset{⋄}{m}}^{*},r] & =\{\Omega (s,r):s\in S_{S}^{*}\}\subset S_{\overset{⋄}{m}}^{*}\subset SS* & \\ 3. & \lim_{r\to 0}\Omega \left[S_{\overset{⋄}{m}}^{*},r\right] & =\left\{\lim_{r\to 0}\Omega \left(s,r\right):s\in S_{S}^{*}\right\}\subset \bar{S_{\overset{⋄}{m}}^{*}}\subset S_{S}^{*} & \\ 4. & & =\left\{s:s\in S_{S}^{*}\right\}\subset \bar{S_{\overset{⋄}{m}}^{*}}\subset S_{S}^{*} & \\ 5. & & =S_{S}^{*}\subset \bar{S_{\overset{⋄}{m}}^{*}}\subset S_{S}^{*} & \\ 6. & & =\bar{S_{\overset{⋄}{m}}^{*}}=S_{S}^{*} & (\mathrm{By}\;\mathrm{def}.\;\mathrm{of}\;\subset ) \end{array}$$

   □

This directly gives us our goal.

**Theorem** **A3** (The Polymutator Behavior Set is Rational)**.**
*$S_{\overset{⋄}{m}}^{*}$ is a rational set of behaviors.*

**Proof.** 

$$\begin{array}{cccc} 1. & \left|\;S_{\overset{⋄}{m}}^{*}\right| & ={ℵ}_{0} & (\mathrm{By}\;\mathrm{Lem}.\mathrm{A14})\\ 2. & \bar{S_{\overset{⋄}{m}}^{*}} & =SS* & (\mathrm{By}\;\mathrm{Lem}.\mathrm{A15}) \end{array}$$

   □

## Appendix B. Detailed Methods

We now cover some technical details that are important for understanding the operation of the ARMS algorithm.

### Appendix B.1. Extension and Prior Knowledge

Extension adds new vertices around an existing vertex so as to increase the coverage of G. During extension, the agent randomly samples points $P=\{p_{j}\}$ around the exterior of the field of the vertex v, then geometrically checks whether the path between the agent’s current state and $p_{j}$ is clear ($r_{j}=1$) or not ($r_{j}=0$).

Then, the agent tries to add new fields. Suppose the size of the field F(v) is σ. The agent starts by trying to add new fields with a size of $\sigma^{\prime }=\sqrt{2}\sigma$. It does this by checking the value ${\hat{r}}_{k}=\frac{R_{k}}{C_{k}}$, where

$$\begin{array}{cc} R_{k} & =\sum_{p_{j}\in P}r_{j}\;\cdot \;e^{-\frac{∥p_{k}-p_{j}{∥}^{2}}{\sigma^{\prime }}} \end{array}$$

and

$$\begin{array}{cc} C_{k} & =\sum_{p_{j}\in P}e^{-\frac{∥p_{k}-p_{j}{∥}^{2}}{\sigma^{\prime }}} \end{array}$$

which effectively makes ${\hat{r}}_{j}$ the local “average” of reachability, with “local” defined by the scale of $\sigma^{\prime }$. All $p_{j}$ are evaluated and ordered from highest to lowest ${\hat{r}}_{j}$, and the points with the highest ${\hat{r}}_{j}$ (above a certain threshold) are added as new vertices with fields of size $\sigma^{\prime }$ (candidate vertex fields that are too redundant with older vertices are not added).

After performing this check for $\sigma^{\prime }=\sqrt{2}\sigma$, the agent does the same for $\sigma^{\prime }=\sigma$ and $\sigma^{\prime }=\frac{\sqrt{2}}{2}\sigma$. For the last check, it does not matter if ${\tilde{r}}_{j}$ is above the pre-defined threshold, as the space surrounding F(v) is filled with new vertices of field-size $\frac{\sqrt{2}}{2}\sigma$. The “Astute” agent uses a direct collision detection calculation to determine $r_{j}$, while the “Misled” agent uses $1-{\hat{r}}_{j}$ instead of ${\hat{r}}_{j}$, effectively inverting the judgments of the “Astute” agent.

Here, “prior knowledge” refers only to information used to bias the admission of candidate vertices during graph extension. It is not a prior over the complete dynamics or topology of the environment, nor does it directly alter the controller or the estimated reachability of existing edges. The labels “Astute” and “Misled” describe the source of this local extension bias rather than implying any ordering of agent competence. Because this bias changes which vertices are admitted during graph growth, its effect can interact with the candidate distribution, vertex field scale, redundancy, and deletion mechanism. Consequently, a monotonic relationship between this information and final performance is not guaranteed in the present implementation.

### Appendix B.2. ARMS α ′ Regulation

Internally, information about inhibition dynamics is stored in a tuple

(A9) $$\Lambda =(\alpha^{\prime },\alpha_{0}^{\prime },\Delta^{+},\Delta^{-},\kappa )$$

with three main functions that modify Λ, $f_{1}^{\Lambda }$, $f_{2}^{\Lambda }$, and $f_{3}^{\Lambda }$. Referring back to the ARMS flowchart in Figure 6, there are three points where Λ is updated, those being steps [ p ] (where $f_{1}^{\Lambda }$ is called on the edge that just failed), [ e ] (where $f_{2}^{\Lambda }$ is called after a path has been found), and [ r ] (where $f_{3}^{\Lambda }$ is called on the edge with the highest α that is inhibited after a pathing failure).

$\begin{array}{c} \mathrm{Function A1:}\mathrm{Stochastic}\alpha \mathrm{update}.\\ \;\;\;\mathrm{function}\;d\alpha^{\prime }(\alpha^{\prime },\Delta )\\ \;\;\;\;\;\;\;\;\;n∼U([0,2])\\ \;\;\;\;\;\;\;\;\;\alpha^{\prime }\leftarrow \alpha^{\prime }+n\;\cdot \;\Delta \\ \;\;\;\;\;\;\;\;\;\mathrm{if}\alpha^{\prime }<0\;\mathrm{then}\\ \;\;\;\;\;\;\;\;\;\;\;\;\;\alpha^{\prime }\leftarrow 0\\ \;\;\;\;\;\;\;\;\;\mathrm{return}\alpha^{\prime } \end{array}$

$\begin{array}{c} \mathrm{Function A2:}\mathrm{Traversal-failure}\mathrm{response}.\\ \;\;\;\mathrm{function}\;f_{1}^{\Lambda }(\Lambda ,e)\\ \;\;\;\;\;\;\;\;(\alpha^{\prime },\alpha_{0}^{\prime },\Delta^{+},\Delta^{-},\kappa )\leftarrow \Lambda \\ \;\;\;\;\;\;\;\;\Delta \alpha_{+}^{\prime }\leftarrow \tilde{\alpha }\left(e\right)-\alpha^{\prime }\\ \;\;\;\;\;\;\;\;\Delta^{+}\leftarrow \left(1-\beta_{r}\right)\;\cdot \;\Delta^{+}+\beta_{r}\;\cdot \;\lambda_{\tau }^{+}\;\cdot \;\max\left(0,\Delta \alpha_{+}^{\prime }\right)\\ \;\;\;\;\;\;\;\;\alpha^{\prime }\leftarrow d\alpha^{\prime }\left(\alpha^{\prime },\Delta^{+}\right)\\ \;\;\;\;\;\;\;\;\Lambda \leftarrow (\alpha^{\prime },\alpha_{0}^{\prime },\Delta^{+},\Delta^{-},\kappa )\\ \;\;\;\;\;\;\;\;\mathrm{return}\Lambda \end{array}$

$\begin{array}{c} \mathrm{Function A3:}\mathrm{Pathing-success}\mathrm{response}.\\ \;\;\;\mathrm{function}\;f_{2}^{\Lambda }(\Lambda )\\ \;\;\;\;\;\;\;\;(\alpha^{\prime },\alpha_{0}^{\prime },\Delta^{+},\Delta^{-},\kappa )\leftarrow \Lambda \\ \;\;\;\;\;\;\;\;\mathrm{if}\kappa >0\;\mathrm{then}\\ \;\;\;\;\;\;\;\;\;\;\;\;\Delta \alpha_{-}^{\prime }\leftarrow \alpha_{0}^{\prime }-\alpha^{\prime }\\ \;\;\;\;\;\;\;\;\;\;\;\;\Delta^{-}\leftarrow \left(1-\beta_{r}\right)\Delta^{-}+\beta_{r}\;\cdot \;\lambda_{\tau }^{-}\;\cdot \;\Delta \alpha_{-}^{\prime }\\ \;\;\;\;\;\;\;\;\;\;\;\;\kappa \leftarrow 0\\ \;\;\;\;\;\;\;\;\Lambda \leftarrow (\alpha^{\prime },\alpha_{0}^{\prime },\Delta^{+},\Delta^{-},\kappa )\\ \;\;\;\;\;\;\;\;\mathrm{return}\Lambda \end{array}$

$\begin{array}{c} \mathrm{Function A4:}\mathrm{Pathing-failure}\mathrm{response}.\\ \;\;\;\mathrm{function}\;f_{3}^{\Lambda }(\Lambda ,e)\\ \;\;\;\;\;\;\;\;(\alpha^{\prime },\alpha_{0}^{\prime },\Delta^{+},\Delta^{-},\kappa )\leftarrow \Lambda \\ \;\;\;\;\;\;\;\;\mathrm{if}\kappa =0\;\mathrm{then}\\ \;\;\;\;\;\;\;\;\;\;\;\;\alpha_{0}^{\prime }\leftarrow \alpha^{\prime }\\ \;\;\;\;\;\;\;\;\kappa \leftarrow \kappa +1\\ \;\;\;\;\;\;\;\;\alpha^{\prime }\leftarrow \min\left(\alpha^{\prime },\alpha \left(e\right)\right)\\ \;\;\;\;\;\;\;\;\alpha^{\prime }\leftarrow d\alpha^{\prime }\left(\alpha^{\prime },-\Delta^{-}\right)\\ \;\;\;\;\;\;\;\;\Lambda \leftarrow (\alpha^{\prime },\alpha_{0}^{\prime },\Delta^{+},\Delta^{-},\kappa )\\ \;\;\;\;\;\;\;\;\mathrm{return}\Lambda \end{array}$

### Appendix B.3. Derivation of Δq

While “refinement” is an operation that occurs to an individual *vertex*, the relevant information about when to refine a vertex actually exists on an *edge*, as we are ultimately interested in increasing the resolution of the corresponding ϵ-tubes. Henceforth, when we refer to “refinement”, we will be talking about an operation that occurs *on an edge*, with the decision to “refine an edge” resulting in the refinement of *one of its vertices*. To understand when our agent should refine an edge, we need to understand what exactly the agent gains through refinement. Technically, the overall utility of an edge is related to how helpful the edge is for getting the agent to the rest of the environment, a global measure that depends on all the paths that pass through that edge and their reliability. We can quantify this as

(A10) $$q\left(e\right)=\sum_{p\in P_{G}\left(e\right)}p(p)\;\cdot \;\alpha (p),$$

where $P_{G}\left(e\right)$ is the set of all paths containing e, p(p) is the probability that p is sampled, and α(p) is a generalization of potential affordance to paths, with $\alpha \left(p\right)=r_{K}\left(p\right)\;\cdot \;M\left(p\left[0\right]\right)\;\cdot \;M\left(p\left[-1\right]\right)$. To make q(e) tractable to calculate, we have to make some assumptions. First, we assume that $p\left(p\right)\propto r_{K}\left(e\right)$, with an unknown proportionality constant $c_{p}$. Second, we assume that $r_{K}\left(p\right)\propto r_{K}\left(e\right)$, with a proportionality constant of $c_{r}$. Third, we assume that M(p[0]) and M(p[−1]) are independent of each other and of e, so we factor them out into their average across the whole graph, denoted $c_{F}$. Fourth, we assume that the number of paths including e is proportional to the number of traversals over that edge, n(e), giving us:

(A11) $$\begin{array}{cc} q(e) & =\sum_{p\in P_{G}\left(e\right)}p(p)\;\cdot \;\alpha (p)=\sum_{p\in P_{G}\left(e\right)}c_{p}r_{K}\left(e\right)\;\cdot \;r_{K}\left(p\right)\;\cdot \;M\left(p\left[0\right]\right)\;\cdot \;M\left(p\left[-1\right]\right)\\ \; & =\sum_{p\in P_{G}\left(e\right)}c_{p}r_{K}\left(e\right)\;\cdot \;c_{r}r_{K}\left(e\right)\;\cdot \;c_{F}=c_{p}c_{r}c_{F}\;\cdot \;\sum_{p\in P_{G}\left(e\right)}r_{K}{\left(e\right)}^{2}=c_{q}\;\cdot \;n\left(e\right)\;\cdot \;r_{K}{\left(e\right)}^{2} \end{array}$$

where $c_{q}=c_{p}c_{r}c_{F}$. Assuming that refining the edge will split it into *k* smaller edges $\left\{e_{1}^{\prime },...e_{k}^{\prime }\right\}$ with disjoint fields, the total “utility” of these refined edges would be

(A12) $$q_{\mathrm{ref}}\left(e\right)=\sum_{i=1}^{k}q(e_{i}^{\prime })=\sum_{i=1}^{k}c_{q}n\left(e_{i}^{\prime }\right)r_{K}{\left(e_{i}^{\prime }\right)}^{2}.$$

We assume that each “child” edge $e_{i}^{\prime }$ is in approximately $\frac{1}{k}$th as many paths as e and that $r_{K}\left(e_{i}^{\prime }\right)∼\mathrm{Beta}\left(c\;\cdot \;r_{K}\left(e\right),c\;\cdot \;\left(1-r_{K}\left(e\right)\right)\right)$ (the beta distribution re-parameterized with $r_{K}\left(e\right)$ as the average of the distribution and *c* a parameter encoding the intrinsic properties of the state space). Taking k→∞ for analytical tractability, we get

(A13) $$\begin{array}{cc} q_{\mathrm{ref}}\left(e\right) & =\sum_{i=1}^{k}c_{q}n\left(e_{i}^{\prime }\right)r_{K}{\left(e_{i}^{\prime }\right)}^{2}=\sum_{i=1}^{k}c_{q}\frac{n(e)}{k}r_{K}{\left(e_{i}^{\prime }\right)}^{2}=c_{q}\;\cdot \;n\left(e\right)\;\cdot \;\frac{1}{k}\sum_{i=1}^{k}r_{K}{\left(e_{i}^{\prime }\right)}^{2}\\ \; & =c_{q}\;\cdot \;n\left(e\right)\;\cdot \;E\left[r_{K}{\left(e_{i}^{\prime }\right)}^{2}\right]=c_{q}\;\cdot \;n\left(e\right)\;\cdot \;\left(\frac{r_{K}\left(e\right)\left(1-r_{K}\left(e\right)\right)}{c+1}+r_{K}{\left(e\right)}^{2}\right). \end{array}$$

The difference between these two terms, q(e) and $q_{\mathrm{ref}}\left(e\right)$, gives the expected utility of refining an edge:

(A14) $$\begin{array}{cc} \Delta q(e) & =q_{\mathrm{ref}}\left(e\right)-q\left(e\right)\\ \; & =\left[c_{q}\;\cdot \;n\left(e\right)\;\cdot \;\left(\frac{r_{K}\left(e\right)\left(1-r_{K}\left(e\right)\right)}{c+1}+r_{K}{\left(e\right)}^{2}\right)\right]-\left[c_{q}\;\cdot \;n\left(e\right)\;\cdot \;r_{K}{\left(e\right)}^{2}\right]\\ \; & =c_{q}\;\cdot \;n\left(e\right)\;\cdot \;\left(\frac{r_{K}\left(e\right)\left(1-r_{K}\left(e\right)\right)}{c+1}+r_{K}{\left(e\right)}^{2}-r_{K}{\left(e\right)}^{2}\right)\\ \; & =4c_{\Delta \tilde{q}}n\left(e\right)r_{K}\left(e\right)\left(1-r_{K}\left(e\right)\right) \end{array}$$

where $c_{\Delta \tilde{q}}=c_{q}/\left[4\left(c+1\right)\right]$ absorbs the unknown proportionality terms. The factor of 4 normalizes the Bernoulli-variance term, since $4r_{K}\left(e\right)\left(1-r_{K}\left(e\right)\right)\in \left[0,1\right]$. One small caveat is that the agent must estimate $r_{K}\left(e\right)$ as ${\tilde{r}}_{K}\left(e\right)$, but pseudo-counts are unhelpful in this case. Avoiding them and rearranging the terms gives

(A15) $$\Delta \tilde{q}\left(e\right)=4c_{\Delta \tilde{q}}n\left(e\right)\frac{n_{s}\left(e\right)}{n(e)}\frac{n_{f}\left(e\right)}{n(e)}\approx 4c_{\Delta \tilde{q}}\frac{n_{s}\left(e\right)n_{f}\left(e\right)}{n(e)+\epsilon },$$

with ϵ being a constant for numerical stability (we arbitrarily pick ϵ=1).

This score, an indicator of how useful it would be to refine an edge, is actually quite intuitive. An edge that is never used (or has never been used) is unlikely to benefit from refinement. If an edge is completely reliable *or* completely unreliable, then $\Delta \tilde{q}\left(e\right)=0$ and it is useless to refine the edge. If the edge is of intermediate reliability, then $\Delta \tilde{q}\left(e\right)>0$, because refining the edge creates the possibility that some child edges will be *more* reliable than the parent and others less so; these lesser children can be filtered out (inhibited), allowing our agent to separate the wheat from the chaff, so to speak.

### Appendix B.4. Unobserved Vertices and Vertex Deletion

A state that has never been entered by the agent has a special status in the ARMS system. Any edge $e_{i,j}=\left(v_{i},v_{j}\right)$ for which $v_{i}$ has not been visited is considered a *ghost edge*. Such an edge is considered to have zero epistemic value, and is inhibited until $v_{i}$ is visited. Not maintaining this “ghost/non-ghost” distinction can cause the agent to fixate on impossible-to-reach goals.

Once the agent has reached the maximum number of vertices that its memory matrix can allow, in order to keep progressing it needs a way to delete useless vertices. To determine this, we develop an ad hoc score which incorporates the fraction of the edges that a vertex v has which are “ghosts”, $F_{\mathrm{ghost}}\left(v\right)$. This score is computed as follows:

(A16) $$g\left(v\right)=-\frac{\left(1-F_{\mathrm{ghost}}\left(v\right)\right)\left(n_{\mathrm{transits}}\left(v\right)+1\right)\left(n_{\mathrm{visits}}\left(v\right)+1\right)\left(M\left(v\right)+1\right)}{\sqrt{T_{\mathrm{last}}\left(v\right)}+1}$$

where $n_{\mathrm{transits}}\left(v\right)$ is the number of times the agent successfully entered and then subsequently left v, $n_{\mathrm{visits}}\left(v\right)$ is the number of times v has been visited, and $T_{\mathrm{last}}\left(v\right)$ is the time since v was last visited. When the agent wants to add a new vertex but no space is left, it deletes the vertex which has the maximum *g*.

This deletion rule is a practical finite-memory heuristic. Because vertex deletion breaks the containment property of the ideal mutator, the completeness guarantee derived for the idealized construction does not extend to the implemented ARMS algorithm.

### Appendix B.5. Pathfinding Algorithm

The $P_{\;⊚}$ pathfinding algorithm is based on recursive problem decomposition via wave-based midpoint finding. The algorithm can be described by the following functions.

$\begin{array}{c} \mathrm{Function A5:}\mathrm{Find}\mathrm{midway}\mathrm{vertices.}.\\ \;\;\;\mathrm{function}\;\mathrm{FIND\_MID}(m_{s},m_{g},G)\\ \;\;\;\;\;\;\;\;f_{s}\leftarrow m_{s},\;f_{g}\leftarrow m_{g}\\ \;\;\;\;\;\;\;\;\mathrm{while}\;\mathrm{True}\;\mathrm{do}\\ \;\;\;\;\;\;\;\;\;\;\;\;f_{s}\leftarrow f_{\mathrm{prop}}\left(G,f_{s}\right)\\ \;\;\;\;\;\;\;\;\;\;\;\;\mathrm{if}\;f_{s}⊙f_{g}\neq 0\;\mathrm{then}\\ \;\;\;\;\;\;\;\;\;\;\;\;\;\;\;\;\mathrm{return}\;f_{s}⊙f_{g}\\ \;\;\;\;\;\;\;\;\;\;\;\;f_{g}\leftarrow f_{\mathrm{prop}}\left(G,f_{g}\right)\\ \;\;\;\;\;\;\;\;\;\;\;\;\mathrm{if}\;f_{s}⊙f_{g}\neq 0\;\mathrm{then}\\ \;\;\;\;\;\;\;\;\;\;\;\;\;\;\;\;\mathrm{return}\;f_{s}⊙f_{g}\\ \;\;\;\;\;\;\;\;\;\;\;\;\mathrm{if}\;f_{s}=0\;\mathrm{OR}\;f_{g}=0\;\mathrm{then}\\ \;\;\;\;\;\;\;\;\;\;\;\;\;\;\;\;\mathrm{return}\;0 \end{array}$

$\begin{array}{c} \mathrm{Function A6:}\mathrm{Find}\mathrm{path}\mathrm{to}\mathrm{goal}.\\ \;\;\;\mathrm{function}P_{⊚}(m_{s}^{\prime },m_{g}^{\prime },G)\\ \;\;\;\;\;\;\;\;\mathrm{stack}\leftarrow [],\;p\leftarrow []\\ \;\;\;\;\;\;\;\;m_{s}\leftarrow m_{s}^{\prime },\;m_{g}\leftarrow m_{g}^{\prime }\\ \;\;\;\;\;\;\;\;\mathrm{while}\;\mathrm{True}\;\mathrm{do}\\ \;\;\;\;\;\;\;\;\;\;\;\;m\leftarrow \mathrm{FIND\_MID}(m_{s},m_{g},G)\\ \;\;\;\;\;\;\;\;\;\;\;\;\mathrm{if}\;m=0\;\mathrm{then}\\ \;\;\;\;\;\;\;\;\;\;\;\;\;\;\;\;\mathrm{return}\;\mathrm{None}\\ \;\;\;\;\;\;\;\;\;\;\;\;\mathrm{if}\;m⊙m_{g}=0\;\mathrm{then}\\ \;\;\;\;\;\;\;\;\;\;\;\;\;\;\;\;\mathrm{stack.push}\left(m_{g}\right)\\ \;\;\;\;\;\;\;\;\;\;\;\;\;\;\;\;m_{g}\leftarrow m\\ \;\;\;\;\;\;\;\;\;\;\;\;\mathrm{else}\\ \;\;\;\;\;\;\;\;\;\;\;\;\;\;\;\;p\leftarrow \mathrm{RANDOM}(m⊙m_{s})\\ \;\;\;\;\;\;\;\;\;\;\;\;\;\;\;\;p.\mathrm{append}(p)\\ \;\;\;\;\;\;\;\;\;\;\;\;\;\;\;\;m_{s}\leftarrow p\\ \;\;\;\;\;\;\;\;\;\;\;\;\;\;\;\;m_{g}\leftarrow \mathrm{stack.pop()}\\ \;\;\;\;\;\;\;\;\;\;\;\;\;\;\;\;\mathrm{if}\;p⊙m_{g}^{\prime }\neq 0\;\mathrm{then}\\ \;\;\;\;\;\;\;\;\;\;\;\;\;\;\;\;\;\;\;\;\mathrm{return}\;p \end{array}$

The RANDOM function returns a random one-hot vector from a multi-hot vector, and corresponds to random selection of a vertex from a set of vertices.

### Appendix B.6. Maze Generation

We use two different kinds of random mazes: the first are mazes laid out on a rectilinear lattice, while the second are randomly distributed within the boundaries of a specific polygon. In both cases, we generate a set of “node” points (on a grid for rectilinear mazes and randomly but evenly distributed using Lloyd’s algorithm [20] for polygonal mazes) for which we compute the Delaunay triangulation, forming a planar graph. The topology of the maze is determined by randomly selecting a spanning tree of this graph. The interior of the maze is determined just by the geometric union of many “hallway” polygons corresponding to edges of this graph, with a small amount of “smoothing” to reduce the complexity of the polygon so as to speed up collision detection.

### Appendix B.7. Estimating Graph Reliability R(G)

In the text, we say that the reliability of a segraph R(G) measures the ability of the agent to go from any point in a state space to any other point in the state space. Technically, this is not true, for two reasons: first, we restrict ourselves to the subset of state space that is physically *reachable* by the agent, meaning that we only include points that are inside of the maze; second, there is a continuum of points within the maze, so measuring the reliability for all points is computationally impossible. We compromise by selecting “special” points in the maze, which correspond to the “node” points used for constructing the maze in the first place.

So, consider two node points from the maze, $p_{1}$ and $p_{2}$. We map these points to vertices of G, $v_{1}$ and $v_{2}$, respectively. If either of these points is not inside a vertex field of G, then the reliability of G for going between $p_{1}$ and $p_{2}$ is marked as 0. Otherwise, A uses the $P_{\;⊚}$ algorithm to find a path across the graph, using a value of $\alpha^{\prime }$ that is a decaying running average of the actual $\alpha^{\prime }$ used by the agent up until the point of measurement. If no path can be found, the agent uses its regular mechanisms for lowering the threshold until a path can be found. We then simulate the agent following the path. If the sampled trajectory hits a wall, we use the normal mechanism for handling the traversal failure of inhibiting the failed edge and raising $\alpha^{\prime }$, then looking for a new path. If at any point no path can be found, the reliability of the graph for going from $p_{1}$ to $p_{2}$ is marked as 0.

The “true” length of the path between $p_{1}$ and $p_{2}$ is determined with the graph used to initially generate the maze, denoted $d(p_{1},p_{2})$. If the reliability of the path is $r\left(p_{1},p_{2}\right)\in \left\{0,1\right\}$ and the set of node-points for the maze is *P*, then the formula for graph reliability is given as

(A17) $$R\left(G\right)=\frac{1}{\sum_{p_{1},p_{2}\in P}d\left(p_{1},p_{2}\right)}\sum_{p_{1},p_{2}\in P}d\left(p_{1},p_{2}\right)\;\cdot \;r\left(p_{1},p_{2}\right).$$

Because the reported mazes use 25 node points, each scheduled estimate of R(G) evaluates up to $25^{2}=625$ ordered point pairs. Each nonzero-distance pair requires pathfinding, simulated path traversal, and re-pathing after failed edges. This measurement workload is why R(G) was evaluated every 10,000 simulation timesteps rather than at every timestep.

### Appendix B.8. Evaluation Metrics and Reporting

No single metric captures every relevant aspect of ARMS. We use task-specific primary outcomes together with measures of task completion frequency, variability, learning dynamics, and computational cost. Table A1 summarizes the rationale and principal limitation of each reported metric.

**Table A1:** Rationale and limitations of the principal empirical evaluation metrics.

Metric	Rationale	Principal Limitation
Graph reliability R(G)	The maze experiments ask whether the agent can navigate between arbitrary reachable points, rather than merely reach one fixed goal. R(G) therefore evaluates ordered pairs of maze-node points, assigns zero reliability when coverage or pathfinding fails, and weights successful pairs by their true maze distance so that successful long-range navigation contributes more than local movement. Its normalization to [0,1] makes results interpretable across the tested mazes.	It is an offline diagnostic evaluated on the finite set of maze-construction nodes. It does not measure path optimality, movement cost, segraph size, or the online frequency with which arbitrary navigation goals are actually selected.
Per-episode reward	Reward is the standard external objective of the MountainCar environments and incorporates both task accomplishment and the action costs incurred while attempting it. It therefore permits a task-level comparison with PPO+.	Reward combines goal attainment, episode duration, and action penalty. In the harder environments, an agent can repeatedly reach the goal while retaining a negative total reward.
Reward-event rate	The number of goal-reward events within each reporting bin directly measures how frequently the task is completed. It is reported in addition to total reward so that successful goal attainment remains visible when action penalties dominate the reward value.	It treats all successful completions equally and does not measure the path length, action cost, or quality of an individual completion.
Wall-clock runtime	Runtime measures the practical computational cost of executing a complete 200,000-timestep trial and exposes efficiency differences that are not represented by task performance alone.	It depends on hardware and implementation details and does not separately identify the cost of pathfinding, graph mutation, controller execution, or performance measurement.

For each task outcome, we report the trajectory over time rather than only the final value, allowing differences in convergence and recovery to remain visible. The main figures use medians and interquartile ranges because trial distributions can be skewed, while means, sample standard deviations, and pointwise bootstrap confidence intervals are provided in Appendix C. The present evaluation does not include path length, energy expenditure, segraph memory usage, performance under observation or actuator noise, or transfer to previously unseen task families. Consequently, the reported metrics evaluate navigability, task accomplishment, variability, learning dynamics, and computational runtime but should not be interpreted as a comprehensive evaluation of every potentially relevant performance dimension.

### Appendix B.9. Hyperparameter Selection

#### Appendix B.9.1. ARMS Hyperparameters

Table A2 summarizes the main parameters of ARMS that were varied experimentally or differed across different sets of experiments. Other details are available in the released machine-readable configuration files and the code implementation itself.

These parameters were selected through intuition-guided experimentation rather than a standardized optimization procedure. For the 2D maze experiments, the same configuration was used across all conditions, with the exception of the explicit variation of $\sigma_{0}$ in Figure 7D. The 3D and 4D experiments used their own configuration, with $\sigma_{0}=0.3$ and $p_{\mathrm{persist}}=0.1$ rather than the 2D values of 1.5 and 0.6, respectively. Together with the differences in maze construction and simulated movement described above, this means that the 2D, 3D, and 4D experiments do not constitute a controlled comparison of the isolated effect of dimensionality on ARMS performance. In contrast, the comparison between the original stress score and scaled failure-count refinement criterion was performed while holding all other parameters constant within each dimension-by-prior-knowledge condition.

A minor note on the code implementation: the released configuration files store the reciprocal $c_{\Delta \tilde{q}}^{-1}$ rather than $c_{\Delta \tilde{q}}$. This is an implementation detail rather than a distinct scientific parameter.

**Table A2:** Main ARMS parameters used in the reported experiments. Values in the MountainCar column are those used in Figure 9, with swept values shown in parentheses.

Parameter	2D Mazes	3D & 4D Mazes	MountainCar	Role and Qualitative Effect
Initial field scale $\sigma_{0}$	1.5	0.3	0.5	Sets the initial representational resolution of the segraph. Larger values produce an initially coarser representation, smaller values produce an initially finer representation. Figure 7D additionally tests {0.3,1.5,7.5} in a 2D maze.
Refinement sensitivity $c_{\Delta \tilde{q}}$	0.455	0.455	2.0 (0.455,2.0)	Scales the stress score used to trigger refinement. Smaller values cause refinement to require more accumulated evidence.
Goal persistence $p_{\mathrm{persist}}$	0.6	0.1	1.0 (0.6,1.0)	Probability of persisting in trying to reach the current goal following a failed edge traversal.
$\Lambda_{0}$ values $\left(\alpha_{0}^{\prime },\Delta_{0}^{+},\Delta_{0}^{-},\beta_{r}\right)$	(0.5,0.01,0.5,0.1)	(0.5,0.01,0.5,0.1)	(0.5,0.01,0.5,0.1)	The initial affordance threshold and its adaptive upward and downward step sizes.
Refinement and extension scale	$\sqrt{2}$	$\sqrt{2}$	$\sqrt{2}$	Determines the change in representational resolution produced by refinement and extension.
Segraph vertex capacity $N_{\max}$	2000	2000	2000	Maximum number of vertices in the agent’s segraph. Adding a vertex after the capacity has been reached causes other vertices to start being deleted.

#### Appendix B.9.2. ARMS MountainCar Hyperparameter Selection

For MountainCar, the Cartesian product of $c_{\Delta \tilde{q}}\in \left\{0.455,2.0\right\}$ and $p_{\mathrm{persist}}\in \left\{0.6,1.0\right\}$ was evaluated separately in each of the MountainCar environments (0 through 3). Each of the four configurations was evaluated in 16 independent 200,000-timestep trials. This corresponds to 64 runs and 12.8 million environmental timesteps per environment, or 256 runs and 51.2 million timesteps across the four environments.

A single shared configuration, $c_{\Delta \tilde{q}}=2.0$ and $p_{\mathrm{persist}}=1.0$, was selected by qualitative inspection of its overall performance across the four environments relative to the best-performing PPO+ configuration in each environment. Because every ARMS grid condition had already been evaluated with 16 trials, the selected condition was not rerun; instead, its 16 grid search trials are the trials reported in Figure 9. Thus, the four MountainCar environments were not held out from ARMS parameter selection.

Figure A1 reports the reward and reward event rate for all four ARMS configurations. Although their learning trajectories and reward rates differ (especially early on), successful task completion is observed throughout the tested grid. This constitutes a two-parameter sensitivity analysis, but does not establish robustness to parameters outside this small grid or to interactions with other ARMS parameters.

#### Appendix B.9.3. PPO+ Hyperparameter Selection

PPO+ was tuned independently in each environment using an exhaustive grid search over the following hyperparameter values.

**Hyperparameter**	**Values**
learning rate	$\left\{10^{-4},10^{-3},10^{-2},10^{-1}\right\}$
rollout length	{64,128,256}
discount factor	{0.99,0.999}
number of minibatches	{8,16,32}
entropy coefficient	$\left\{10^{-5},10^{-4},10^{-3},10^{-2}\right\}$
intrinsic-reward coefficient	$\left\{10^{-7},10^{-5},10^{-3}\right\}$
RND learning rate	$\left\{10^{-5},10^{-4},10^{-3},10^{-2}\right\}$

The Cartesian product of these different values results in 3456 configurations for each environment; each configuration was evaluated over five 200,000-timestep trials in each environment, resulting in 17,280 experiments per environment, 69,120 experiments overall, and a total of 13.824 billion timesteps. For each environment, we selected the configuration that produced the greatest aggregate number of environment reward events across the five trials per configuration. The selected configurations were subsequently evaluated in a separate set of trials not used for hyperparameter selection. These final trials were used for comparison against ARMS.

## Appendix C. Statistical Validation and Runtime Benchmarks

Final-time maze comparisons are reported in Table A3 and Table A4; controlled refinement-criterion comparisons in Table A5; and MountainCar comparisons in Table A6.

Time-resolved maze comparisons are shown in Figure A2 and Figure A3; controlled refinement-criterion comparisons in Figure A4; and MountainCar comparisons in Figure A5.

Mean trajectories with standard deviations and confidence intervals are shown for the mazes in Figure A6 and Figure A7, respectively, and for MountainCar in Figure A8 and Figure A9, respectively. Maze and MountainCar runtimes are shown in Figure A10 and Figure A11, respectively.

Because simulation seeds were independently generated and were not matched across conditions, all statistical tests treat trials as independent. All tests were two-sided with α=0.05. For Figure 7, final R(G) values at 200,000 timesteps were compared using a Kruskal–Wallis test within each panel, with Holm correction across the eight omnibus tests. Pairwise follow-up comparisons used Mann–Whitney *U* tests, with Holm correction both across the three comparisons within each panel and across all 24 pairwise comparisons in the figure. Epsilon-squared correlations are reported as omnibus effect sizes, while rank-biserial correlations are reported as pairwise effect sizes. For Figure 9, PPO+ and ARMS were compared using Mann–Whitney *U* tests for the final common point of the smoothed reward trace and the final reward event rate bin in each environment, with Holm correction across all eight environment-by-metric comparisons. Only completed trials were included.

For the time-resolved Figure 7 analysis, Kruskal–Wallis tests and epsilon-squared effect sizes were calculated at each recorded time after t=0. The analytically imposed initial condition R(G)=0 was displayed but was not tested. Family-wise error was controlled using 20,000 whole-trajectory permutations of the maximum *H* statistic, both within each panel across time and across all panels and times. Pairwise Mann–Whitney tests were additionally Holm-corrected within each panel across pairs and time and across the complete figure. For Figure 9, Mann–Whitney tests and rank-biserial effect sizes were calculated at 201 points along each smoothed reward trajectory and across the 49 reward-event-rate bins. Panel-wide and figure-wide family-wise error were controlled using 20,000 whole-trajectory permutations of the maximum absolute tie-corrected *U* statistic.

Means, sample standard deviations, and pointwise percentile-bootstrap 95% confidence intervals for the mean were calculated from the same trial-level observations using 10,000 bootstrap resamples. These descriptive summaries complement the median and interquartile-range summaries in the main figures; the confidence intervals are pointwise rather than simultaneous confidence bands.

Wall-clock benchmarks were calculated from the recorded duration of each completed 200,000-timestep trial. Runtime summaries include the mean, sample standard deviation, median, quartiles, interquartile range, range, and a percentile-bootstrap 95% confidence interval for the mean. Figure 7 runtime comparisons used Kruskal–Wallis and pairwise Mann–Whitney tests with the same panel and figure-level Holm families described above. Figure 9 runtime comparisons used Mann–Whitney tests with Holm correction across the four environments. The available logs do not permit separate wall-clock attribution to pathfinding or R(G) measurement, but their operation-level computational costs are characterized in Appendix D.

For the controlled refinement criterion comparison, the original stress score and scaled failure-count conditions were compared using two-sided Mann–Whitney *U* tests within each dimension-by-prior-knowledge combination, with Holm correction across the six final-time comparisons. Rank-biserial effects are oriented original score minus failure count. Time-resolved comparisons were performed at every empirical measurement after t=0, with panel-wide and figure-wide family-wise error controlled using 20,000 whole-trajectory permutations of the maximum absolute tie-corrected *U z* statistic. The analytically imposed t=0 value was displayed but not tested.

**Table A3:** Final-time Kruskal–Wallis comparisons for Figure 7. Sample sizes are ordered according to the three conditions shown in each panel. $p_{\mathrm{Holm}}$ is corrected across the eight omnibus tests.

Panel	*n*	*H*	$\epsilon^{2}$	*p*	$p_{\mathrm{Holm}}$
5 × 5(1) Maze	8/8/8	0.323	0.000	0.8508	1.000
5 × 5(2) Maze	8/8/8	3.715	0.0817	0.1561	0.6244
Triangle Maze	8/8/8	4.174	0.1035	0.1241	0.6203
Pentagon Maze	8/8/8	0.000	0.000	1.000	1.000
Maze Swap	8/8/8	5.185	0.1516	0.07485	0.4491
System Robustness	8/8/8	1.049	0.000	0.5919	1.000
3D Mazes	16/16/16	15.159	0.2924	0.0005109	0.003576
4D Mazes	16/16/16	21.714	0.4381	$1.93\times 10^{-5}$	0.0001542

**Table A4:** Pairwise follow-up comparisons for the significant 3D and 4D final-time omnibus results. Positive $r_{\mathrm{rb}}$ indicates that the first condition had higher R(G). $p_{\mathrm{Holm}}$ is corrected across all 24 pairwise comparisons in Figure 7.

Panel	Comparison	$n_{1}/n_{2}$	*U*	$r_{\mathrm{rb}}$	*p*	$p_{\mathrm{Holm}}$
3D	Naive-Astute	16/16	231	0.805	0.000112	0.00257
3D	Naive-Misled	16/16	202	0.578	0.005603	0.1177
3D	Astute-Misled	16/16	126	−0.016	0.9549	1.000
4D	Naive-Astute	16/16	19	−0.852	$4.33\times 10^{-5}$	0.00104
4D	Naive-Misled	16/16	54	−0.578	0.005603	0.1177
4D	Astute-Misled	16/16	205	0.602	0.003937	0.08660

**Table A5:** Final-time controlled comparisons of the original stress score and scaled failure-count refinement criterion. Rank-biserial effects are oriented original stress score minus failure count, so positive values favor the original score. $p_{\mathrm{Holm}}$ is corrected across all six dimension-by-prior-knowledge comparisons.

Dimension	Condition	$n_{\mathrm{original}}/n_{n_{f}}$	*U*	$r_{\mathrm{rb}}$	*p*	$p_{\mathrm{Holm}}$
3D	Naive	16/16	202	0.578	0.005603	0.02241
3D	Astute	16/16	91	−0.289	0.1689	0.5068
3D	Misled	16/16	208	0.625	0.002733	0.01367
4D	Naive	16/16	41	−0.680	0.001114	0.006683
4D	Astute	16/13	98	−0.058	0.8094	0.9713
4D	Misled	16/16	109	−0.148	0.4856	0.9713

**Table A6:** Final-point comparisons between PPO+ and ARMS in Figure 9. All comparisons use n=16 trials per algorithm. Rank-biserial effects are oriented PPO+ minus ARMS, so positive values favor PPO+ and negative values favor ARMS. $p_{\mathrm{Holm}}$ is corrected across all eight environment-by-metric comparisons.

Environment	Outcome	Higher	*U*	$r_{\mathrm{rb}}$	*p*	$p_{\mathrm{Holm}}$
MountainCar-0	Reward	PPO+	242	0.891	$1.89\times 10^{-5}$	0.000113
MountainCar-0	Reward rate	PPO+	252	0.969	$2.64\times 10^{-6}$	$1.84\times 10^{-5}$
MountainCar-1	Reward	ARMS	65	−0.492	0.01849	0.02174
MountainCar-1	Reward rate	ARMS	56.5	−0.559	0.007247	0.02174
MountainCar-2	Reward	ARMS	57	−0.555	0.007882	0.02174
MountainCar-2	Reward rate	ARMS	54	−0.578	0.004941	0.01976
MountainCar-3	Reward	ARMS	0	−1.000	$1.54\times 10^{-6}$	$1.24\times 10^{-5}$
MountainCar-3	Reward rate	ARMS	15.5	−0.879	$2.23\times 10^{-5}$	0.000113

## Appendix D. ARMS Complexity Analysis

Since ARMS is an online search algorithm that is meant to perform a theoretically unbounded search across the set of rational behaviors, there is no well-defined finite worst-case bound on the time it will take to find a solution. Instead, we characterize the computational costs of its primary operations. Let n=|V| and m=|E| be the number of segraph vertices and segraph edges, respectively, and let $N_{\max}$ be the fixed maximum number of vertices in the segraph (a limitation introduced to bound the size of the code implementation’s memory matrices), meaning that $n\leq N_{\max}$ and $m\leq n^{2}$. Let *d* be the dimension of *S*, the agent’s state space. For this analysis, we treat environment simulation as external to ARMS and denote the separately incurred cost of each low-level controller update by $C_{K}$.

With a sparse graph representation, the calculation of various edge scores, affordance thresholding, and goal selection would require O(m) time, while locating the agent in the segraph by testing segraph vertex membership would take O(nd) time. However, the present implementation stores edge variables in fixed-size tensors of size $N_{\max}\times N_{\max}$, meaning that operations such as global scoring, inhibition, and goal selection require $\Theta (N_{\max}^{2})$ time. Updates to the counts associated with a given edge occur in O(1) time, even though the traversal of the associated edge could trigger more costly operations.

The most expensive operation is pathfinding with the $P_{\;⊚}$ algorithm. The $P_{\;⊚}$ algorithm propagates an activation wave over the active adjacency matrix and recursively decomposes the pathfinding problem through midpoint vertices on the segraph. Let L≤n be the length of the returned path. For a given active adjacency matrix (determined by affordance inhibition and manual inhibition or disinhibition), all of the shortest available paths will have length *L*, so the returned path will be one among the shortest available paths. In the current implementation using full tensors, each propagation will cost $\Theta (N_{\max}^{2})$. Assuming that the midpoint decomposition is approximately balanced, it will take O(Llog(L)) such propagations to find the full path, giving a full runtime complexity of

$$C_{P_{\;⊚}}=O\left(N_{\max}^{2}L\log\left(L\right)\right)\leq O\left(N_{\max}^{3}\log\left(N_{\max}\right)\right).$$

If *q* successive threshold lowerings are required to find a path in the event that no path is initially found due to an overly strict affordance threshold, then the planning cost is in general $qC_{P_{\;⊚}}$.

Extension and refinement will generally be less frequent compared to pathfinding, but will incur their own substantial computational costs due to the practical requirement that new vertices should not be redundant with existing vertices. The current implementation samples potential center points of new vertices, either inside the vertex (for refinement) or in a *d*-annulus around the vertex (for extension). Then, a redundancy criterion is checked for each potential new vertex against all existing vertices, with each passing vertex added to the segraph. Let $k_{r}$ and $k_{e}$ be the number of candidate points for new vertices created during refinement and extension, respectively. Screening each candidate vertex against the existing segraph costs $O(dk_{r}n)$ for refinement and $O(dk_{e}n)$ for extension, and checking the candidates against one another costs $O(dk_{r}^{2})$ for refinement and $O(dk_{e}^{2})$ for extension. Therefore, each refinement or extension takes approximately

$$\begin{array}{cc} C_{\mathrm{ext}} & =O\;\left(dk_{e}n+dk_{e}^{2}+k_{e}dN_{\max}^{2}+k_{e}^{2}\right),\\ C_{\mathrm{ref}} & =O\;\left(dk_{r}n+dk_{r}^{2}+k_{r}dN_{\max}^{2}+k_{r}^{2}\right). \end{array}$$

When the vertex capacity has been reached ($n=N_{\max}$), deletion requires checking a score for which computation depends on the ghost-score of each vertex, which itself requires checking each vertex’s edges, which amounts to a check of every edge, requiring O(m) time.

Over a duration of *T* simulation timesteps, let $P_{T}$, $X_{T}$, $R_{T}$, and $D_{T}$ be the numbers of pathfinding calls, extension events, refinement events, and capacity-triggered vertex deletion events, respectively, over *T* timesteps. Let $C_{K}$ be the cost of a single controller update. A very general description of the total runtime would then be

(A18) $$O\left(TC_{K}+Tnd+P_{T}C_{P_{\;⊚}}+X_{T}C_{\mathrm{ext}}+R_{T}C_{\mathrm{ref}}+D_{T}m\right).$$

For memory requirements, a sparse segraph would require O(nd+m) persistent memory; however, the current implementation uses a vectorized representation and stores edges and vertices of the segraph (and associated values) in constant-sized tensors, so the actual persistent memory footprint is $\Theta (N_{\max}^{2}+N_{\max}d)$.

## References

[1] Turing A.M. (1936). On computable numbers, with an application to the Entscheidungsproblem. Proc. Lond. Math. Soc..

[2] Peer M., Brunec I.K., Newcombe N.S., Epstein R.A. (2021). Structuring knowledge with cognitive maps and cognitive graphs. Trends Cogn. Sci..

[3] Schaul T., Horgan D., Gregor K., Silver D. Universal value function approximators. Proceedings of the International Conference on Machine Learning, PMLR.

[4] Andrychowicz M., Wolski F., Ray A., Schneider J., Fong R., Welinder P., McGrew B., Tobin J., Abbeel P., Zaremba W., Guyon I., von Luxburg U., Bengio S., Wallach H., Fergus R., Vishwanathan S., Garnett R. (2017). Hindsight experience replay. Advances in Neural Information Processing Systems.

[5] Ecoffet A., Huizinga J., Lehman J., Stanley K.O., Clune J. (2021). First return, then explore. Nature.

[6] Forestier S., Portelas R., Mollard Y., Oudeyer P.Y. (2022). Intrinsically motivated goal exploration processes with automatic curriculum learning. J. Mach. Learn. Res..

[7] Lehman J., Stanley K.O. (2011). Abandoning objectives: Evolution through the search for novelty alone. Evol. Comput..

[8] Pugh J.K., Soros L.B., Stanley K.O. (2016). Quality diversity: A new frontier for evolutionary computation. Front. Robot. AI.

[9] Kalman R.E. (1960). A new approach to linear filtering and prediction problems. J. Basic Eng..

[10] Vincent P., Larochelle H., Bengio Y., Manzagol P.A. Extracting and composing robust features with denoising autoencoders. Proceedings of the 25th International Conference on Machine Learning.

[11] Fan L., Zhang F., Fan H., Zhang C. (2019). Brief review of image denoising techniques. Vis. Comput. Ind. Biomed. Art.

[12] Takens F., Rand D., Young L.S. (1981). Detecting strange attractors in turbulence. Proceedings of the Dynamical Systems and Turbulence, Warwick 1980.

[13] Sutton R.S., Precup D., Singh S. (1999). Between MDPs and semi-MDPs: A framework for temporal abstraction in reinforcement learning. Artif. Intell..

[14] Gibson J.J., Shaw R., Bransford J. (1977). The theory of affordances. Perceiving, Acting, and Knowing: Toward an Ecological Psychology.

[15] LaValle S.M. (1998). Rapidly-Exploring Random Trees: A New Tool for Path Planning.

[16] Kavraki L.E., Svestka P., Latombe J.C., Overmars M.H. (1996). Probabilistic roadmaps for path planning in high-dimensional configuration spaces. IEEE Trans. Robot. Autom..

[17] Schrittwieser J., Antonoglou I., Hubert T., Simonyan K., Sifre L., Schmitt S., Guez A., Lock E., Hassabis D., Graepel T. (2020). Mastering atari, go, chess and shogi by planning with a learned model. Nature.

[18] Hubert T., Schrittwieser J., Antonoglou I., Barekatain M., Schmitt S., Silver D. Learning and planning in complex action spaces. Proceedings of the International Conference on Machine Learning, PMLR.

[19] Bellemare M., Srinivasan S., Ostrovski G., Schaul T., Saxton D., Munos R. (2016). Unifying count-based exploration and intrinsic motivation. Adv. Neural Inf. Process. Syst..

[20] Lloyd S. (1982). Least squares quantization in PCM. IEEE Trans. Inf. Theory.

